# Validation of FRET Assay for the Screening of Growth Inhibitors of *Escherichia coli* Reveals Elongasome Assembly Dynamics

**DOI:** 10.3390/ijms160817637

**Published:** 2015-07-31

**Authors:** René van der Ploeg, Spyridon Theodoros Goudelis, Tanneke den Blaauwen

**Affiliations:** Bacterial Cell Biology, Swammerdam Institute for Life Sciences, University of Amsterdam, Science Park 904, 1098 XH Amsterdam, The Netherland; E-Mails: renevanderploeg@gmail.com (R.P.); sthgoudelis@gmail.com (S.T.G.)

**Keywords:** antibiotics, A22, drug targets, FRET, Gram-negative, growth inhibitors, MreB, PBP2, RodA, RodZ

## Abstract

The increase in antibiotic resistant bacteria demands the development of new antibiotics against preferably new targets. The common approach is to test compounds for their ability to kill bacteria or to design molecules that inhibit essential protein activities *in vitro*. In the first case, the mode of action of the drug is unknown and in the second case, it is not known whether the compound will pass the impermeable barrier of the bacterial envelope. We developed an assay that detects the target of a compound, as well as its ability to pass the membrane(s) simultaneously. The *Escherichia coli* cytoskeletal protein MreB recruits protein complexes (elongasomes) that are essential for cell envelope growth. An in cell Förster Resonance Energy Transfer (FRET) assay was developed to detect the interaction between MreB molecules and between MreB and the elongasome proteins RodZ, RodA and PBP2. Inhibition of the polymerization of MreB by *S*-(3,4-dichlorobenzyl) isothiourea (A22) or of the activity of PBP2 by mecilinam resulted in loss or reduction of all measured interactions. This suggests that the interactions between the elongasome proteins are governed by a combination of weak affinities and substrate availability. This validated in cell FRET assay can be used to screen for cell envelope growth inhibitors.

## 1. Introduction

Multidrug resistance, increasingly, is the cause of failure to cure people from Gram-negative bacterial infections [1]. The era of easy drug development is long passed and the discovery of natural products that kill bacteria is researched extensively without yielding sufficient promising new drugs during the last 30 years of research [2]. Nevertheless, plenty of natural products are still available for antibacterial testing and computer assisted drug screening and design will help to diminish the number of drugs to be assayed *in vitro* and *in vivo*. Assays that screen simultaneously for the passage of the compounds through the very impermeable Gram-negative bacterial envelope, as well as identify the target of the drug immediately, are much more efficient and failure-proof. Our Förster Resonance Energy Transfer (FRET) assay aims to be of this last category.

The fluorescent protein revolution has provided us with fluorescent probes that can be expressed in living bacteria. These proteins can be genetically fused to proteins of interest in order to determine their temporal and spatial dynamics in the cell. Presently, fluorescent proteins come in all conceivable colors and therefore can also be used to measure the interaction of proteins in living cells [3]. To obtain FRET, the emission spectrum of the donor fluorophore should have a considerable overlap with the excitation spectrum of the acceptor fluorophore [4]. If this requirement is met, excitation of the donor fluorophore will result in direct transfer of energy from the donor fluorophore to the acceptor fluorophore, which will then emit fluorescence. This is only possible when the two fluorophores are very close to each other, as the FRET efficiency is decreasing with the distance between the fluorophores to the power of six [4]. Because the chromophores of the fluorescence proteins are located in the center of their beta-barrel structure, the minimal distance between the two chromophores will be 5 nm. Therefore, only proteins that are interacting directly or are localizing adjacent will yield a FRET signal. Once it is established that two proteins interact using a FRET assay, the loss of this interaction due to the presence of inhibitors can be easily monitored by the loss of the FRET signal. Consequently, the in cell FRET assay screens simultaneously for inhibitors that are able to pass the envelope as well as for a specific target to be inhibited.

Protein complexes that are termed elongasomes are involved in the growth of the *Escherichia coli* three-layered envelope (cytoplasmic membrane, peptidoglycan layer and outer membrane) [5,6]. The peptidoglycan is a continuous network of glycan strands that are interconnected by peptide side bridges and is essential for viability. Inhibition of the activity of proteins in the elongasomes results in spherical growth and ultimately cell death [7,8,9]. Penicillin Binding Proteins (PBPs) are part of these complexes and antibiotics against these proteins have saved the lives of innumerable people. An assay that would screen for the loss of protein interactions within these complexes would be able to find completely new classes of antibiotics that might be as effective in killing bacteria as the well-known Penicillins. To this purpose we have developed and validated an FRET assay that screens for the loss of protein interactions involved in cell envelope synthesis in *E. coli*. As proof of principle, the depolymerization of the actin homologue MreB, which is essential for length growth, was used.

MreB is present in the cell in high numbers (Table 1) and is capable of forming polymeric structures, *i.e.*, filaments [10,11]. Through its short amphipathic helix it is able to attach to the membrane [12]. The current view is that MreB polymers form filaments that can differ in length [13]. The localization and length of these filaments in the cell determines the position of the peptidoglycan synthesizing elongasomes and the rod-shape morphology of the cells [13,14]. Peptidoglycan building blocks are synthesized in the cytoplasm. After the last step in Lipid II peptidoglycan precursor synthesis by MurG in the cytoplasm, the integral membrane protein RodA is expected to translocate Lipid II across the membrane [15,16] to provide the substrate for PBP2 and PBP1A [17,18]. PBP1A is a bifunctional class A PBP with glycosyltransferase activity that polymerizes glycan strands from the precursor saccharide moiety lipid II and with transpeptidase activity that cross-links peptides between adjacent glycan strands. PBP2 is a class B PBP with transpeptidase activity that is essential for cell elongation. The bi-topic membrane protein MreC and the integral membrane protein MreD are also essential for length growth. The exact function of these proteins is not known but bacterial two-hybrid studies have shown that MreC interacts as well with MreB as with MreD [19]. Depletion of MreB, MreC, MreD, RodZ, RodA or inhibition of PBP2 give similar changes in shape from rod to sphere [8,19,20] and are therefore thought to be part of the elongasome. MreC seems to be required for the localization of PBP2 [21] and in some bacterial species they have been shown to interact [22]. RodZ is a conserved [23,24,25], bi-topic membrane protein with an 80 amino acids cytoplasmic domain that interacts with monomeric as well as with filamentous MreB [26]. RodZ has a 200 amino acids periplasmic domain that can be deleted without loss of viability but the cells are shorter and rounder under normal growth conditions [24,25,27]. Cells can survive without RodZ but mutations are needed to regain a rod-shape [28].

The small molecule *S*-(3,4-dichlorobenzyl) isothiourea (A22) [9,29] specifically disrupts the MreB polymerization and we used this capacity to determine whether our FRET assay was able to measure the depolymerization of MreB filaments *in vivo.* The ability to form polymers is crucial for the cellular function of MreB [8,30]. A model of a crystal structure of MreB with the inhibitor A22 docked in the ATP binding pocket suggests that it blocks the access of the β- and γ-phosphate of ATP [9]. Consequently, A22 likely competes with ATP for MreB binding and the A22 bound MreB molecules cannot participate in MreB polymerization. The interaction between MreB molecules was chosen as proof of principle for the assay, as it is well established by *in vitro* and *in vivo* studies that MreB forms polymers in the cell that are dissociated by A22 [8,10,31,32].

After establishing that the FRET method as such is not affected by changes in the morphology of the cells, functional fluorescent protein fusions to MreB [25,26] and other elongasome proteins were made and used to measure their interaction in the bacterial cell. Exposing the cells to A22 for one third of a mass doubling time led to a complete loss of FRET efficiency between the MreB proteins. This establishes that the FRET assay is suitable for the screening of drugs that inhibit protein interactions. Interestingly, we noticed that the inhibition of MreB polymerization by A22 or of the d,d-transpeptidase activity of PBP2 by mecillinam for two third of a mass doubling time, both caused the disassembly of MreB polymers and dissociation of the other elongasome proteins. PBP2 dependent peptidoglycan incorporation is apparently needed for stable elongasomes and intact MreB polymers. This extends the use of the MreB FRET assay to the screening of elongasome inhibitors in general.

## 2. Results and Discussion

### 2.1. Förster Resonance Energy Transfer (FRET) Couple Super Yellow Fluorescent Protein 2 (SYFP2) and mCherry

**Figure 1:**
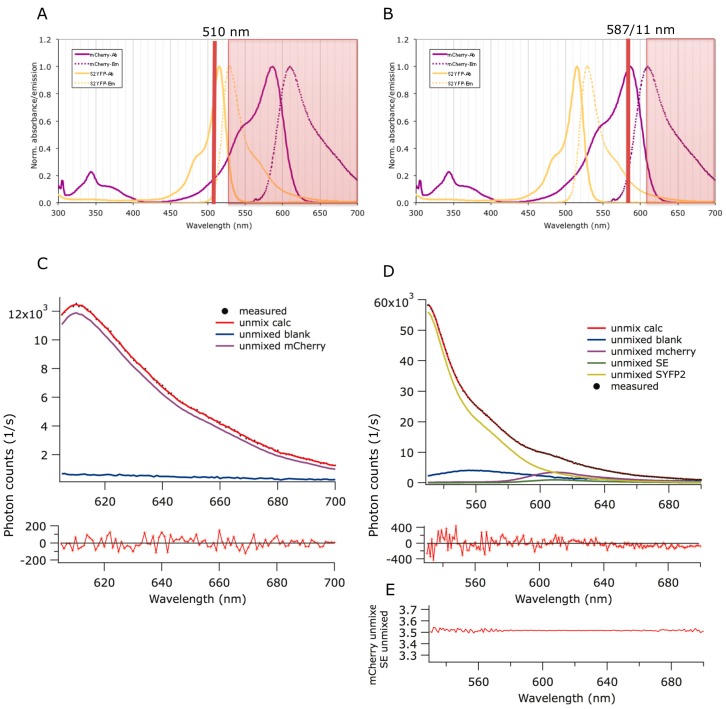
The principle of Förster Resonance Energy Transfer (FRET) analysis for the Super Yellow Fluorescent Protein 2 (SYFP2)-mCherry pair in cells measured in a cuvette. (**A**) SYFP2 and mCherry excitation at 510 nm; (**B**) SYFP2 and mCherry excitation at 587 nm; (**C**) The measured and unmixed mCherry acceptor spectrum; (**D**) The measured and unmixed SYPF2 and mCherry FRET pair sample spectra; (**E**) Ratio of the mCherry spectrum and the sensitized emission spectrum.

Transfer of excitation energy only happens when the two fluorescent fusion proteins are sufficiently close together, between 0 and 10 nm [33]. That property makes FRET a very useful tool to monitor dynamics of protein–protein interactions. A change in distance will give a corresponding change in energy transfer. To measure these immediate changes in distance, Super Yellow Fluorescent Protein 2 (SYFP2) and mCherry fluorescent proteins were selected to setup our FRET system. These fluorescent proteins have the characteristics to mature fast within 15 min and have a significant spectral overlap. Together with their other spectral properties they have a Förster radius (R_0_, the distance at which 50% FRET occurs) of 5.81 nm in water (Figure 1), which is relatively high given that the optimized Cyan and Yellow Fluorescent Protein (CFP and YFP) FRET pairs have a Förster radius range between 5 and 5.83 nm [33,34]. The high brightness of SYFP2 (excitation maximum: 510 nm; extinction coefficient: 105 × 10^3^; emission maximum: 527 nm; and the quantum yield: 0.68 [35]) makes it a good donor fluorescent protein for intensity based detection systems. mCherry (excitation maximum: 587 nm; extinction coefficient: 72 × 10^3^; emission maximum: 610 nm; and the quantum yield: 0.22 [36]) is useful because it has an emission spectrum in the red spectral area where little auto-fluorescence is created by the bacteria. Therefore, this fluorescent protein combination was used to study the interaction of proteins in the bacterial cell by determining changes in acceptor intensity (for an explanation of the principle of FRET analysis for this pair, see Figure 1).

To avoid overexpression of the fluorescent protein fusions, they were expressed from low copy number plasmids with a weak promoter (*trc* down). A tandem fusion of SYFP2 and mCherry was used as positive control. The bitopic cytoplasmic membrane proteins PBP1A and PBP3 that do not interact were used as negative control in each experiment. The FRET pair plasmids were transformed to wild type strain MC4100. In case of the tandem fusion, a so-called empty second plasmid that does not express any protein was co-transformed. Background fluorescence was derived from MC4100 that was transformed with two empty plasmids. The strains were grown to steady state in minimal medium at 28 °C with a mass doubling time of approximately 180 min and subsequently induced with 15 μM β-d-1-thiogalactopyranoside (IPTG) for 6 h. The optical density was kept below 0.3 in all experiments. The cells were fixed while shaking in the water bath, washed and kept in Phosphate Buffered Saline (PBS) during the measurements. Of each sample, first the amount of mCherry was determined by exciting the sample at 587 nm. Next, the FRET signal was measured by exiting SYFP2 at 510 nm (Figure 1). By spectral unmixing of the spectra [37] the sensitized mCherry emission and the FRET efficiency were calculated (Figure 1).

Fluorescence or Förster Resonance Energy Transfer (FRET) can only occur when a considerable overlap exists between the emission spectrum of the donor fluorescent protein and the acceptor protein. Based on this overlap and assuming a random orientation of the donor and acceptor dipoles (κ^2^ is 2/3), the distance R_0_ between the fluorophores that will result in 50% FRET can be calculated. For the used pair SYFP2 and mCherry this is 5.81 nm. In Figure 1A the normalized excitation (solid) and emission (dashed) spectra for SYPF2 and mCherry are shown in yellow and purple, respectively. Upon inspection of the excitation spectra of both proteins, it is clear that it is impossible to excite SYFP2 without exciting mCherry as well. This cross-excitation contributed to the mCherry fluorescence in the FRET experiment. To be able to subtract the direct excitation contribution from the measured FRET signal it is vital to know exactly how much mCherry is present in the cells. Therefore, the sample is first excited at 578 nm (Figure 1B), which is the maximum of the mCherry excitation spectrum. The cellular background fluorescence is determined by measuring the same spectrum in a sample in which the two plasmids without fluorescent fusion proteins are present (so-called empty vectors). This information is used to dissect the measured spectrum (black dots) in its two components; the cellular autofluorescence (blue) and the mCherry spectrum (purple) revealing the exact amount of mCherry in the cells (Figure 1C). Subsequently, the cells are excited at 510 nm (Figure 1D), which is the maximum of the SYFP2 excitation spectrum. When the two fluorescent proteins are close enough because of the direct interaction between the proteins they are fused to, the SYFP2 will transfer its energy to mCherry. The cells with the empty vectors give again the contribution of the cellular autofluorescence at 510 nm and the expected amount of direct mCherry excitation has been determined by exciting the sample at 587 nm. Because the shapes of the SYFP2 and mCherry emission spectra are known, the measured spectrum (Figure 1D, black dots) can be dissected in its individual components; the cellular autofluorescence (blue), SYFP2 fluorescence (yellow), the direct excitation of mCherry (purple) and the sensitized emission of mCherry or the FRET signal (green). The residuals of the fit to the measured spectra by the calculated spectra based on the sum of the unmixed individual contributions is shown below the graphs in Figure 1C,D of the measured spectra for the excitation at 587 and 510 nm, respectively. As an extra quality control, the graph in Figure 1E shows that the ratio between the mCherry spectrum and that of the sensitized emission is constant, which is to be expected when the sensitized emission is a mCherry spectrum.

### 2.2. Spectral FRET Is Insensitive to Shape Changes

The FRET assay used is based on the measurement of spectra from a concentrated sample of bacteria in a cuvette. Because the wavelength used to excite the fluorescent protein in the bacteria is about one quarter of the size of the bacteria, the photons will collide with the bacteria and will be scattered in all directions. This light contributes to the detected fluorescence signal that is measured at an angle of 90° form the incident light. The bacteria change from rod-shape to spherical or filamentous upon inhibition of length growth or of cell division, respectively. This change in shape could affect the contribution by the light scattering to the measured fluorescence signal during the experiment. By using filters the excitation light is blocked from being detected. However, this never excluded 100% of the light. Therefore, we first determined whether the FRET signal was sensitive to shape changes. The class B PBP2 is essential for cell elongation [38], forms a dimer and its interaction can easily be measured with spectral FRET [39]. PBP2 is a bitopic membrane protein with its N-terminus in the cytoplasm and its active domain in the periplasm. Because its dimerization state is expected not to change during shape changes of the cells, the dimer was used to investigate whether scattering due to shape changes could prevent the measurement of reliable FRET data. Wild-type *E. coli* MC4100 cells were transformed with monomeric Kusabira Orange (mKO)-PBP2 and mCherry-PBP2 (both fused to the N-terminus of PBP2 causing the fluorophores to be in cytoplasm) and the FRET efficiency of this pair was measured in exponentially growing rod-shaped cells, spherical cells and filamenting cells. The combination of mKO and mCherry has been extensively described [37] and the experimental procedure apart from the spectrophotometer filter setting is very similar to that with SYFP2, which has been used for all other measurements presented in this study since mKO, in contrast to SYFP2, cannot be used in unfixed cells. Spherical cells were obtained by incubation with the MreB inhibitor A22 [29] or by the PBP2 inhibitor mecillinam [40] and cells were filamented by inhibiting PBP3 with its specific inhibitor aztreonam [41]. PBP3 is a class B PBP that is essential for cell division in *E. coli* [38,42]. The FRET efficiency of the PBP2 dimer is about 10% in normal rod-shaped cells and this value did not change significantly in the spherical or filamentous cells (Table S1). It can be concluded that the FRET method is not sensitive to shape changes and can be used to screen for drugs that affect the morphology of the cells.

### 2.3. MreB Interactions Are Lost in the Presence of S-(3,4-Dichlorobenzyl) Isothiourea (A22)

**Figure 2:**
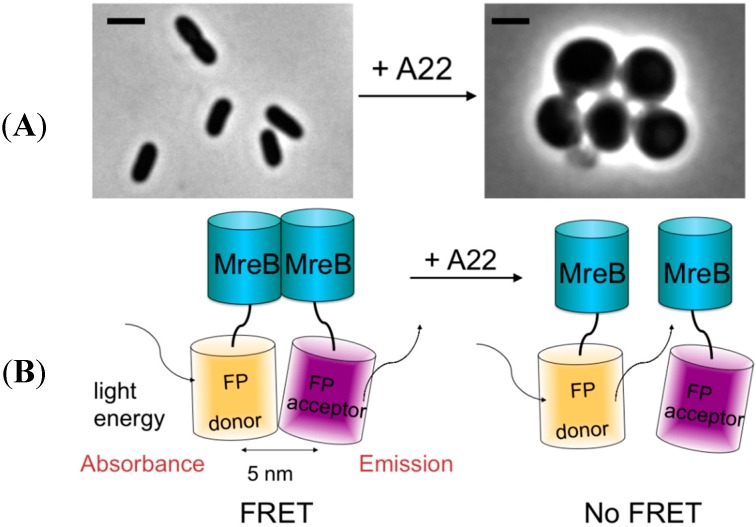
The principle of the in cell FRET antibiotic-screening assay. The interaction between MreB molecules is essential for normal rod-shape growth in wild type cells. Inhibition of this interaction by *S*-(3,4-dichlorobenzyl) isothiourea (A22) results in spherical growth and eventually cell death (**A**). The scale bar equals 2 μm; (**B**) A donor fluorescent-protein SYFP2 fused to MreB and an acceptor fluorescent-protein mCherry fused to MreB are expressed in the cells. Excitation of the donor fluorophore (crooked arrow) will result in direct transfer of its energy to the acceptor fluorophore, which will then emit fluorescence (crooked arrow). Because the fluorescent proteins consist of a β barrel of 5 nm in width with their chemophores embedded in the center, the minimal FRET distance between the two fluorophores will always be 5 nm. FRET will therefore only be measured if the proteins are between 5 and 10 nm distance from each other. This requirement is met when the MreB proteins polymerize and form a filament underneath the cytoplasmic membrane that supports the assembly of the elongasome. Addition of an inhibitor (A22) that prevents MreB polymerization will result in the loss of acceptor fluorescence or FRET. The assay provides therefore immediate information on whether a compound is able to pass the envelope of the cells and on the nature of its target.

To investigate whether the FRET assay can be used to effectively measure the dissociation of morphogenetic protein complexes, the well-characterized inhibitor, A22, of MreB was used as a test case (Figure 2). A fully functional fluorescent protein (FP) MreB protein fusion was described [25] in which the FP was inserted between helix 6 and 7 of MreB. Therefore, we made two MreB sandwich fusions replacing the original FP described by Bendezu and coworkers by SYFP2 (MreB^SYFP2^) and mCherry (MreB^mCherry^). Each fusion was expressed from a different low copy plasmid (Table S3).

**Figure 3:**
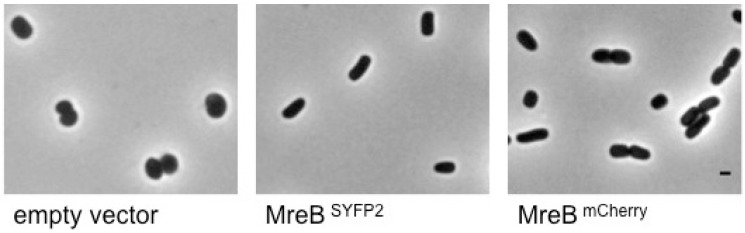
The MreB^mCherry^ and MreB^SYFP2^ sandwich fusions are able to restore the rod-shape of the MreB deficient strain. The phase contrast images are from living cells grown overnight in trypton yeast (TY) with chloramphenicol at 28 °C, supplemented with 60 μM β-d-1-thiogalactopyranoside (IPTG). In each image the strain PA340-678 with a deleted *mreBCD* operon and transformed by the plasmid pMEW1 that expressed MreCD from their endogenous promoter is shown. The difference between the images is the presence of the pSAV057 plasmid with an IPTG inducible promoter. This plasmid contains either (from left to right) no gene insert, the MreB^SYFP2^ or the MreB^mCherry^ sandwich fusion encoding genes. The scale bar equals 1 μm.

To test the functionality of the sandwich fusions they were expressed in an *mreBCD* deletion strain that harbored a plasmid with the *mreCD* genes under the control of their native promoter. The spherical shape of the mutant strain was restored to wild-type rod-shape after overnight growth in trypton yeast (TY) medium at 28 °C with 60 μM IPTG induction (Figure 3). In the strain MC4100 used for the FRET experiments wild-type localization of the proteins was observed with epi-fluorescence microscopy (Figure S1). In addition, diffuse cytosolic localization was confirmed after addition of 10 μg/mL of A22 (Figure S1).

The MreB fusion proteins were expressed in the wild-type laboratory strain MC4100 grown in minimal glucose medium at 28 °C. Protein expression was induced with 15 μM IPTG for about 6 h. After 0, 5, 20, 60 and 120 min of growth in the presence of A22 cells were fixed and subsequently washed and kept in PBS during the measurements. Of each sample, first the amount of mCherry was determined by exciting the sample at 587 nm. Next, the FRET signal was measured by exiting SYFP2 at 510 nm (Figure S1). By spectral unmixing of the data the sensitized mCherry emission and the FRET efficiency were calculated. As a control mecillinam was used that specifically inhibits the transpeptidase activity of PBP2 [40] and which was not expected to have an effect on the polymerization of MreB. A22 and mecillinam convert rod-shaped cells to spherical cells at the same rate [8]. After 60 min (*i.e.*, approximately 1/3 of the mass doubling time) in the presence of A22 or mecillinam, the cells did not show a significant shape change, whereas after 120 min both cell types were somewhat more spherical than the untreated rod-shaped cells (Figure S1).

**Figure 4:**
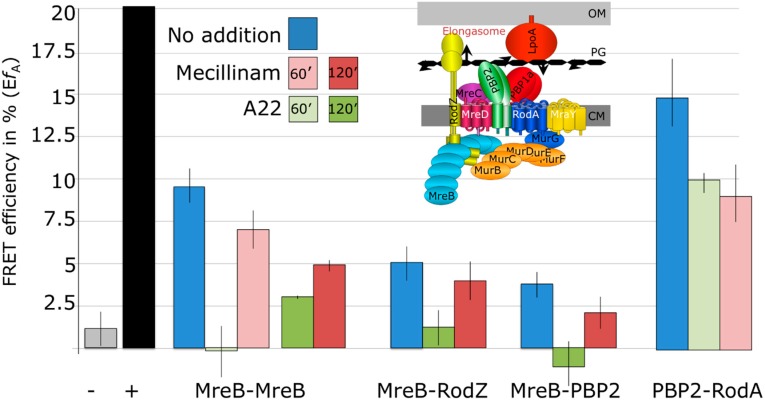
Interactions within the elongasome are affected by the MreB inhibitor A22 and by the PBP2 inhibitor mecillinam. MC4100 cells transformed with two plasmids that expressed the SYFP2 donor and the mCherry acceptor, respectively, fused to the indicated elongasome proteins were grown to steady state at 28 °C. At an optical density at 450 nm of 0.2 the cells were fixed and washed as described in the materials and methods and concentrated to an OD_450_ of 1.0. Spectra excited at 587 and 590 nm were collected and unmixed in contribution of the background, SYFP2 donor, direct excitation of the mCherry acceptor and the mCherry sensitized emission (Figure 1). Sensitized emission was used to calculate the apparent efficiency of energy transfer *Ef_A_* plotted on the vertical axes (with the standard error of the mean). Blue is the FRET efficiency in the absence of inhibitors, green in the presence of A22 for 60 min (light green) or for 120 min (dark green) and red in the presence of mecillinam for 60 min (light red) or for 120 min (dark red). The black bar shows the FRET efficiency of a direct fusion between SYFP2 and mCherry as a positive control and the grey bar shows the FRET efficiency between fusions to PBP3 and PBP1A that do not interact (see Table S2 for the exact data and the number of independent repeats for each sample and the supplementary dataset for all unmixed spectra). For convenience a schematic representation of proteins that are involved in length growth in *E. coli* is inserted.

In the absence of A22 a clear interaction between MreB molecules could be measured with a FRET efficiency of about 9% (Figure 4 and Table S2). Inhibition of MreB with A22 after 5 and 20 min did not result in a decrease in FRET efficiency. However after 60 min of growth in the presence of A22, the FRET efficiency was zero indicating that the interaction was completely lost (Figure 4, Table S2, and supplementary dataset). Interestingly, growth in the presence of mecilllinam for 60 min reduced the FRET efficiency between MreB molecules to 7%. No effect on protein expression was observed for the MreB fusion proteins in the presences of the inhibitors (Figure S2). Inhibition of protein synthesis by 50 μg/mL spectinomycin for 60 min did not affect the interaction between MreB molecules (Table S2). It can be concluded that the FRET assay can be used to measure the action of antibiotics that inhibit protein–protein interactions.

The observation that the depolymerization of MreB by A22 is not instantaneous might be explained as follows. The ATP pool in the cells is about 3 mM [43], whereas the extracellular A22 concentration is only 36 μM. The reason for this low A22 concentration is that it interferes with other processes in the cell at higher concentrations [8]. Assuming the same K_d_ of 1.5 μM for A22 as found for *Thermotoga maritima* MreB [9] and an approximately 10-fold higher K_d_ for ATP, the interaction of MreB with ATP would be inhibited for about 50% under the given conditions. Therefore, it is not surprising that the depolymerization process is relatively slow and that only after 60 min in the presence of A22 in the medium MreB appears to become fully cytosolic. In the microscopy images in Figure S2 the number of clear MreB foci appear to be already reduced after 5–20 min of growth in the presence of A22. Since, during this period no reduction in the FRET signal is measured, one could speculate that A22 causes first a release off the membrane of MreB and then a depolymerization.

### 2.4. MreB Polymerization and PBP2 Activity Are Essential to Maintain Elongasome Protein Interactions

After 120 min of growth in the presence of mecillinam, the FRET efficiency of the MreB interaction was reduced to about 5%. This suggests that the inactivity of PBP2 does affect the polymerization of MreB. To investigate the effect of the inhibition of MreB polymerization and of PBP2 activity on the interactions these proteins have with other proteins in the elongasome, fluorescent protein fusions were constructed for RodZ and RodA. The interaction of these proteins with MreB and PBP2 was measured in the absence or presence of A22 or mecillinam for 120 min, which is approximately two third of the mass doubling time. A FRET efficiency of 5% was found for the interaction between MreB and RodZ. This interaction was lost after 120 min of growth in the presence of A22 and somewhat but not significantly reduced when PBP2 was inhibited by mecillinam. The loss of interaction between MreB and RodZ in the presence of A22 as measured by FRET, strongly indicates that polymerization of MreB is a requirement for the stable interaction of MreB and RodZ *in vivo*.

For the interaction between PBP2 and MreB a FRET efficiency of 5% was determined. This interaction was lost after growth for 120 min in the presence of A22 and by 50% reduced in the presence of mecillinam. Indicating that MreB dislocates from the membrane when its polymerization is inhibited but possibly also when PBP2 is inactive for a longer time period.

The low FRET efficiency between MreB and RodZ or between MreB and PBP2 is not surprising given the huge imbalance in the number of molecules of the FRET partners of the endogenous proteins in the cells (Table 1). Many of the fluorescent fusion proteins will not contribute to the FRET signal for these pairs.

**Table 1:** Reported protein copy numbers per cell in different growth media.

Protein	Minimal ^a^	Rich ^b^	Reference
PBP1A	135, 116	220, 554	[44,45]
PBP2	58, 76	120, 324	[25,45]
MreB	9.000, 2.393	15.000, 11.304	[8,45]
MurG	168	518	[45]
RodZ	150, 576	650, 1309	[25,45]

^a^ GB1, M9, MOPS minimal are minimal glucose media; ^b^ LB, TY, MOPS complete are rich media (see for details the description in the references).

A strong FRET signal of 14% was found for the interaction between RodA and PBP2, which was significantly reduced to 10% and 8.5% in the presence of A22 and mecillinam for 60 min, respectively. Conditional mutants in *mrd*B and *mrd*A, the genes encoding for RodA and PBP2, respectively, produce the same spherical cells [46] and are both needed for peptidoglycan synthesis when all other PBPs are inhibited [47]. An interaction between PBP2 and RodA was therefore to be expected, but to our knowledge this is the first experimental evidence for such an interaction.

Interestingly, inhibition of the polymerization of MreB by A22 as well as inhibition of the transpeptidase activity of PBP2 by mecillinam affected the FRET efficiency between the assayed elongasome proteins. For instance after 120 min in the presence of A22, the interaction between MreB and PBP2 was completely lost as expected and in the presence of mecillinam this interaction was reduced with 50%. Within 60 min of growth in the presence of A22, where the interaction between MreB molecules was completely lost, the interaction between RodA and PBP2 was reduced with 30% and in the presence of mecillinam a reduction of 40% was found for this FRET pair. These data clearly indicate that the polymerization of MreB affects the assembly dynamics of the elongasome and that an inactive elongasome affects the polymerization state of MreB. Schirner and coworkers [48] showed recently that the availability of Lipid II affects the membrane localization and polymerization of MreB in *Bacillus subtilis*. The topography of insertion of new peptidoglycan in the cylindrical wall of the cells is therefore determined by the availability of substrate as well as the affinity between the various proteins involved in the process. Experimental evidence suggests that substrate availability determines the localization of at least some of morphogenetic proteins [49,50]. Consequently, the affinity between these proteins in the elongasome will likely be in the μM range. This might facilitate the development of interaction-inhibitors as they do not necessarily have to be in the very low nM range to be able to compete with the natural binding partner. The developed MreB FRET assay will not be able to discriminate between depolymerization of MreB and inhibition of elongasomes unless the depolymerization is instantaneous. For screening of antibiotics this does not devaluate the assay because it broadens it application to different types of inhibitors. Those that have an immediate effect on the polymerization of MreB and those that inhibit the activity of the elongasome and cause depolymerization of MreB on a longer time scale.

## 3. Experimental Section

### 3.1. Bacterial Strains and Growth Conditions

All *E. coil* strains and plasmids used in this work are listed in Table S3. For the FRET experiments cells were grown to steady state in glucose minimal medium (GB1) containing 6.33 g of K_2_HPO_4_·3H_2_O, 2.95 g of KH_2_PO_4_, 1.05 g of (NH_4_)_2_SO_4_, 0.10 g of MgSO_4_·7H_2_O, 0.28 mg of FeSO_4_·7H_2_O, 7.1 mg of Ca(NO_3_)_2_·4H_2_O, 4 mg of thiamine, 4 g of glucose and 50 mg of required amino acids, per liter, pH 7.0 at 28 °C as described [51]. Growth inhibitor A22 (Calbiochem, San Diego, CA, USA) was used at a final concentration of 10 mg/L. Mecillinam (Selexid, Leo Laboratories Limited, Hurley, Berkshire, UK) was added to an end concentration of 2 mg/L in all experiments except for the PBP2–PBP2 interactions where 4 mg/L was used. The division inhibitor aztreonam (Sigma-Aldrich, St. Louis, MO, USA) was administered to a total amount of 2 mg/L. For the complementation and localization experiments cells were grown in GB1 or in TY (10 g trypton, 5 g yeast extract, 5 g NaCl per litre, pH 7.0) as specified. Absorbance was measured at 450 nm (GB1) or 600 nm (TY) with a Biochrom Libra S70 spectrophotometer (Harvard Biosciences, Holliston, MA, USA).

### 3.2. Construction of the Plasmids

The plasmid pRP012 was constructed by performing a PCR on the *SYFP2* gene using the primers SYFP2_NcoI_F and SYFP2_no_link_stop_R (see Table S4 for a complete list of primers and their sequences). The forward primer extends the gene at the 3′ end to improve expression; 14 codons are added. At the 5′ end the gene is truncated, the reverse primers last annealing point is 10 codons upstream from the stop codon. This creates a truncated SYFP2 variant that lacks 10 unnecessary amino acids at the downstream end of the gene [52]. The subsequent PCR product was purified using the standard protocol of the PCR purification kit supplied by Roche. The gene was ligated in the *Hind*II site of the high copy plasmid pUC18. After receiving the correct SYFP2 sequence, the gene was cut from the plasmid using *Nco*I and *Bcl*I and ligated with pTHV037 that was opened with *Nco*I and *BamH*I. To allow the expression of both SYFP2 and mCherry in the cytoplasm, SYFP2 was also placed in the pSAV057 plasmid, creating pRP002. This plasmid has the stop codon more downstream adding 24 C-terminal codons.

For the expression of a mCherry-RodZ fusion protein, the plasmid pSAV047 and a RodZ PCR product were cut with *EcoR*I and *Hind*III. The RodZ gene (*yfgA*) was multiplied by PCR with primers RodZ-EcoRI-F and RodZ-HindIII-stop-R. The restricted PCR product was ligated with the linearized pSAV047 plasmid. mCherry-RodA (pRP081) was created by the introduction of RodA in pSAV047 in the same way as for RodZ. This placed the *rodA* gene (*mrdB*) in frame downstream directly behind the *mCherry*, which left a linker of two codons that coded for the *EcoR*I site.

To construct a functional MreB sandwich fusion, we adopted the method of [25], but modified it by using other restriction sites. A short linker, present in primers MreB_Fix_F and MreB_Fix_R, was introduced in the gene directly after codon 231, which placed the linker between helices 6 and 7. The two *mreB* gene fragments were fused via the *EcoR*I site (amino acids glutamic acid and phenyl aline (EF)) present in the linker SGHVEFSGSPGD, resulting in the split *mreB* gene *Nco*I-fragment1-*Psc*I-*EcoR*I-*Bam*HI-fragment2-*Hind*III. The *SYFP2* or *mCherry* genes were digested using restriction sites *Nco*I and *BamH*I and ligated in the split *mreB* gene that was restricted with *Psc*I and *BamH*I, which removed the *EcoR*I site. The hybrids MreB^SYFP2^ and MreB^mCherry^ are expressed from the plasmids pRP057 and pRP059, respectively.

### 3.3. Microscopy and Image Analysis

For imaging the cells were immobilized on 1% agarose [53], and photographed with a CoolSnap *fx* (Photometrics) CCD camera mounted on an Olympus BX-60 fluorescence microscope through an UPLANFl 100x/1.3 oil objective (Tokyo, Japan). Images were acquired with Micro-Manager (www.micro-manager.org/) with direct output of the desired hyperstack structure for ObjectJ (http://simon.bio.uva.nl/objectj), which is an imageJ (http://imagej.nih.gov/ij) plug in, which supports graphical vector objects that non-destructively mark images on a transparent layer [39]. In all experiments the cells were first photographed in phase contrast mode, then with the mCherry filter (FF01-560/14-25 Semrock) and when required with a SYFP2 filter (Yellow GFP, ex. 490–510 nm, Olympus, Tokyo, Japan).

### 3.4. FRET Experiment and Data Analysis

For the FRET experiments we used two fluorescent protein pairs. The mCherry protein [36] as the acceptor combined with the donor protein mKO [54] or with the donor protein SYFP2 [35]. *Escherichia coli* MC4100 was co-transformed with 2 appropriate vectors. When a single protein was to be expressed a none-coding second plasmid was co-transformed. Each experiment included the tandem fusion mKO-mCherry or SYFP2-mCherry as a positive control, SYFP2 and mCherry expressed as separate proteins in the cell, and mCherry-PBP3 expressed with mKO-PBP1A as negative control. Transformed bacteria were grown and prepared for FRET measurements as described [37]. FRET efficiency was measured as described [37] apart from the use of different filters. The following filters were used to obtain mCherry spectra: excitation filter 587/11 nm (587/11 nm BrightLine single-band bandpass filter, Semrock, New York, NY, USA) in combination with a 600 long pass emission filter and for mKO spectra a 541/12 nm excitation filter with a 550 long pass filter and finally for SYFP2 spectra a 510/4 nm excitation combined with a 510 long pass emission filter (Chroma Technology Corp., Bellows Falls, VT, USA). For each experiment reference spectra were recorded from cells expressing only SYFP2, mKO or mCherry proteins, and reference background spectra were recorded from cells bearing two none-coding plasmids. The reference spectra were used to calculate contributions of donor, acceptor and background to the total spectrum of the experimental samples measured at λ_D_ using least square fitting (see Figure 1 for an explanation). Sensitized emission and apparent efficiency of energy transfer *Ef_A_* were derived as described [33,37,55,56]. The FRET efficiency values have been estimated by the method of spectral unmixing as described in [37]. From a set of multiple experiments a mean and standard error of the mean were calculated (Tables S1 and S2 and supplementary data set).

## 4. Conclusions

A FRET assay has been developed and validated that enables the in cell measurement of the loss of interaction of proteins involved in cell envelope growth. The FRET assay is not sensitive to changes in cell shape. The elongasome is a dynamic protein complex of which the protein–protein interactions are affected by the activity of its constituents. The interaction between the elongasomes and the MreB polymers is affected by the inhibition of the transpeptidase activity of PBP2 and by the polymerization activity of MreB.

## Supplementary Materials

Supplementary materials can be found at http://www.mdpi.com/1422-0067/16/08/17637/s1.
