# Supplementary material for: Validation of FRET Assay for the Screening of Growth Inhibitors of Escherichia coli Reveals Elongasome Assembly Dynamics
Source: Int J Mol Sci. 2015 Jul 31;16(8):17637–54. doi: 10.3390/ijms160817637 (PMC4581212; doi:10.3390/ijms160817637)
Supplement: Supplementary file 1 [file ijms-16-17637-s001.zip › ijms-82313-Supplementary Information/ijms-82313-Supplementary 1-for publish.pdf]

Supplementary Information

Table S1. Apparent efficiency of energy transfer Ef_A in percentage between PBP2 proteins in the presence of protein inhibitors that affect the shape of the cells.

240 min	PBP2	PBP2 *A22*	PBP2 *mecillinam*	PBP2 *aztreonam*
PBP2	9.6 ± 0.5 (5)	9.5 ± 1.3 (2)	7.3 ± 1.3 (2)	12.0 ± 1.1 (2)

The functionality of the proteins was inhibited for two mass doublings during growth of MC4100 cells in GB1 minimal medium at a temperature of 28 °C. No significant difference in apparent FRET efficiency between PBP2 molecules of the uninhibited samples and the inhibited samples are observed. The average FRET efficiencies are presented with the variance being the standard error of the mean (SEM) with the number of replicates between brackets. The FRET pair used was mKO-PBP2 and mCherry-PBP2 [37,39].

Table S2. Apparent efficiency of energy transfer Ef_A in percentage of the measured interactions between elongasome proteins.

**60 min**	**MreB**	**MreB A22**	**MreB Mecillinam**	**120 min**	**MreB A22**	**MreB Mecillinam**
MreB	8.9 ± 0.7 (9)	0.3 ± 1.6 (7)	7.0 ± 1.2 (6)	MreB	3.0 ± 0.1 (9)	4.8 ± 0.3 (4)
**120 min**	**PBP2**	**PBP2 A22**	**PBP2 Mecillinam**	**RodZ**	**RodZ A22**	**RodZ Mecillinam**
MreB	3.8 ± 0.8 (5)	−1.2 ± 1.5 (3)	2.0 ± 0.9 (5)	5.0 ± 0.9 (5)	1.2 ± 1.1 (3)	3.9 ± 1.2 (5)
**60 min**	**RodZ**	**RodZ A22**	**RodZ Mecillinam**	**RodA**	**RodA A22**	**RodA Mecillinam**
PBP2	3.1 ± 2.1 (3)	1.4 ± 0.7 (4)	4.0 ± 0.1 (4)	14.0 ± 1.0 (3)	10.1 ± 0.3 (4)	8.4 ± 0.8 (4)

The direct fusion between SYFP2 and mCherry gave a FRET efficiency of 40% ± 2.1% and the separately expressed SYFP2 and mCherry gave a FRET efficiency of 1.0% ± 0.9%. Bystander FRET (*i.e.*, the FRET efficiency between two non-interacting membrane proteins PBP1A and PBP3) was 1.2% ± 0.6% (n = 11). Inhibition of protein synthesis by 50 µg/mL spectrinomycin for 60 min did not affect the interaction between MreB molecules (Ef_A was 6.2 ± 0.1 and 5.7 ± 0.7 without and with spectinomycin, respectively, n = 3). The variant in the FRET efficiency is the standard error of the mean (SEM) with the number of replicates between brackets. mCherry was fused in between helix 6 and 7 of MreB as described in the main text and to the amino terminus of PBP2 in the first column. SYPF2 was fused to the N-terminus of the proteins in the other columns. The protein in first column forms a FRET pair with the proteins in the other columns. The antibiotic incubation time is indicated.

Table S3. Bacterial strains and plasmids.

E. coli Strains	Relevant Characteristics	Reference
DH5α	supE44 ΔlacU169 (Φ80lacZΔM15) hsdR17 recA1 endA1 gyrA96 thi-1 relA1	[57]
MC4100 (LMC500)	F^-, araD139, D(argF-lac)U169deoC1, flbB5301, ptsF25, rbsR, relA1, rpslL150, lysA1	[58]
PA340-678	PA340 $gltB^+$ Δmre-678	[59]
**Plasmids**	**Relevant Properties**	**Reference**
pMEW1	pMW218 with *Kpn*I–*Hind*III fragment from pMEG1 with the *mreC* and *mreD* genes; Km^R	[8]
pTHV037	pTRC99A with a weakened *trc* promoter and downstream a multiple cloning site, pBR322 ori; 4.2 kb; Ap^R	[7]
pRP012	pTHV037-derivative containing the *SYFP2* gene, extended at the 3′ end with 14 codons to improve expression and truncated at the 5′ end with ten codon, ending with a *BamHI* site just before the stop codon	This work

Table S3. *Cont.*

Plasmids	Relevant Properties	Reference
pSAV047	pTHV037-derivative containing the *mCherry* gene	[37]
pWA003	pTHV037-derivative containing the *mCherry* and *mrdA* gene linked by five codons	[39]
pRP038	pTHV037-derivative containing the *mCherry* and *rodZ* gene linked by two codons	This work
pRP059	pTHV037-derivative containing a hybrid gene of *mreB* and *mCherry*. The *mCherry* gene was introduced after codon four of the linker that was placed downstream codon 228 of *mreB*	This work
pRP081	pTHV037-derivative containing the *mCherry* and *mrdB* gene linked by two codons	This work
pSAV057	pTRC99A with a weakened *trc* promoter and downstream a multiple cloning site, p15A ori; 4.4 kb	[37]
pSAV058	pSAV057-derivative containing the *mKO* gene	[37]
pSAV050	pSAV057-derivative containing the *mCherry* and *mKO* hybrid gene linked by five codons	[37]
pWA004	pSAV057-derivative containing the *mKO* and *mrdA* hybrid gene linked by five codons	[37]
pBB004	pSAV057-derivative containing the *mKO* and *mrcA* hybrid gene linked by five codons	[57]
pSA091	pSAV057-derivative containing the *mCherry* and *SYFP2* hybrid gene linked by nine codons	[37]
pRP002	pSAV057-derivative containing the *SYFP2* gene with an 3′ addition of fourteen codons and an extension of twenty four codons at the 5′ end	This work
pRP057	pSAV057-derivative containing a hybrid gene of *mreB* and *SYFP2*. The *SYFP2* gene was introduced after codon four of the linker that was placed downstream codon 228 of *mreB*	This work

Table S4. List of primers that were used.

Primer Name	Sequence	Underlined
SYFP2_NcoI_F	CCCCCCATGGAATTCGAGCTCGGTACCCGGGGGTCCTCTA	*Nco*I
SYFP2_no_link_stop_R *	CCCCCTGATCATTAGGATCCCCCGGCGGCGGTCACG	*Bcl*I, *BamH*I
FP_NcoI_F	GGATCCACCATGGTGAGC	*Nco*I
FP_EcoRI_R	CCCGAATCCCTTGTACAGCTCGTCCATGC	*EcoR*I
MreB_NcoI_F	CGACCCATGGTGAAAAAATTTCGTGG	*Nco*I
MreB_Fix_R *	CGGACTTCATCGCCCGGGGATCCAGAGAATTCGACATGTGTCCAGAACCCGGATAAGC	*BamH*I, *EcoR*I, *Psc*I
MreB_Fix_F *	GCTTATCCGGGTTCTGGACACATGTCGAATTCTCTGGATCCCCGGGCGATGAAGTCCG	*EcoR*I, *BamH*I, *Psc*I
MreB_HindIII_R	GACCTGTTCAGCGAAGAGTAAGCTTTTGC	*Hind*III
RodZ-EcoRI-F	GGGGGGAATTCATGAATACTGAAGCC	*EcoR*I
RodZ-HindIII-stop-R	CCCCCAAGCTTTTACTGCGCCGGTGATTG	*Hind*III

* The restriction sites in the respective order mentioned.

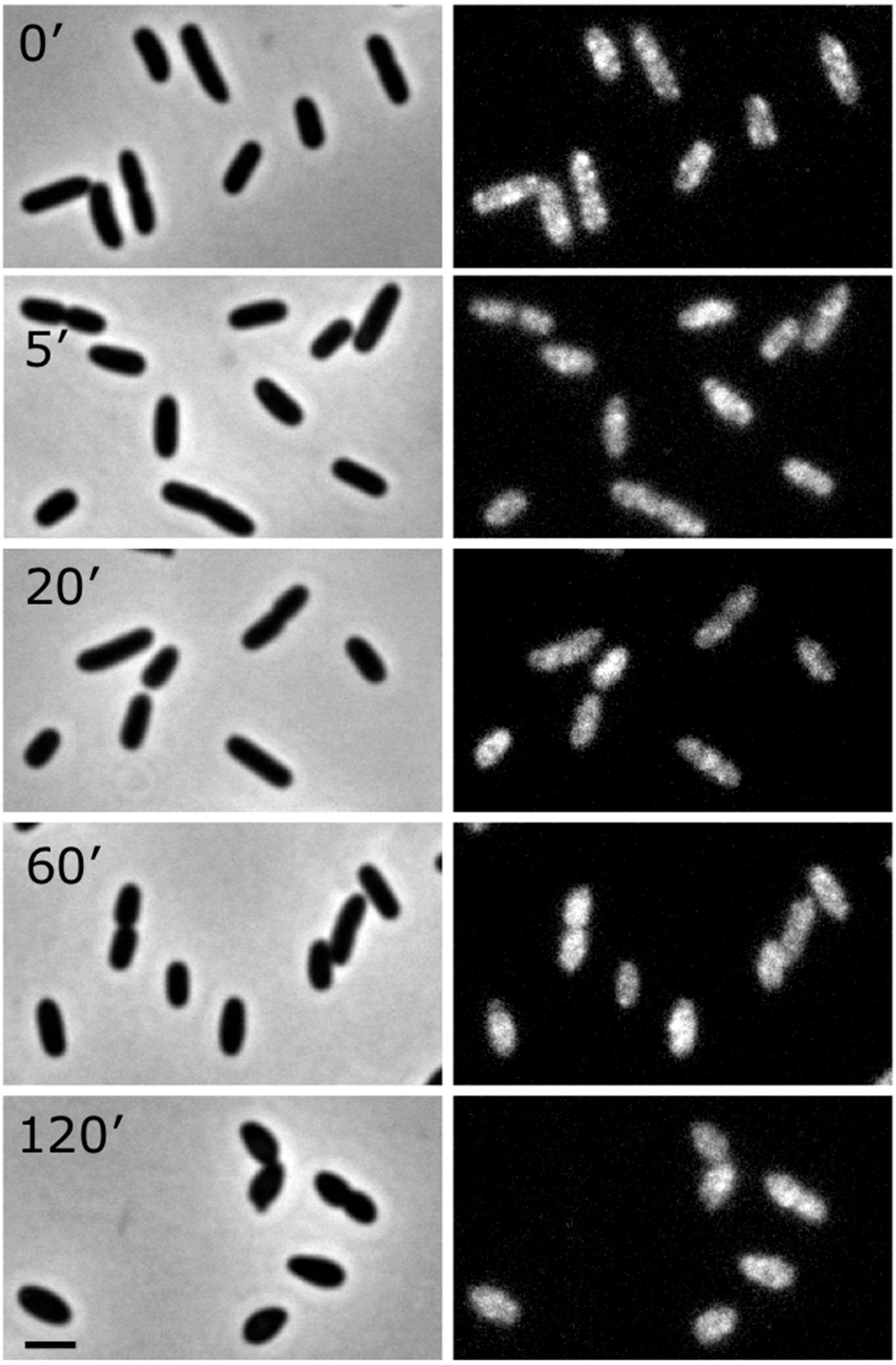

Figure S1. The MreB sandwich fusion rapidly delocalizes in the presence of 10 μg/mL of A22. The MC4100 cells were grown to a steady state followed with induction of protein expression for ±6 h using 15 μM IPTG at a temperature of 28 °C. The inhibitor is administered the indicated time before fixation of the cells. The cells were fixed at an OD_{450} of 0.2 while rotating in a water bath, harvested after 15 min of fixation and subsequently washed in PBS. Partial delocalization of MreB is visible already after 5 min and after 60 min discrete foci are not visible. The morphology of the cells starts to become more spherical after 120 min. On the left phase contract images and on the right their corresponding mCherry images are shown. Only the mCherry channel is shown for convenience, the $MreB^{SYFP2}$ localized similarly. The scale bar equals 2 μm.

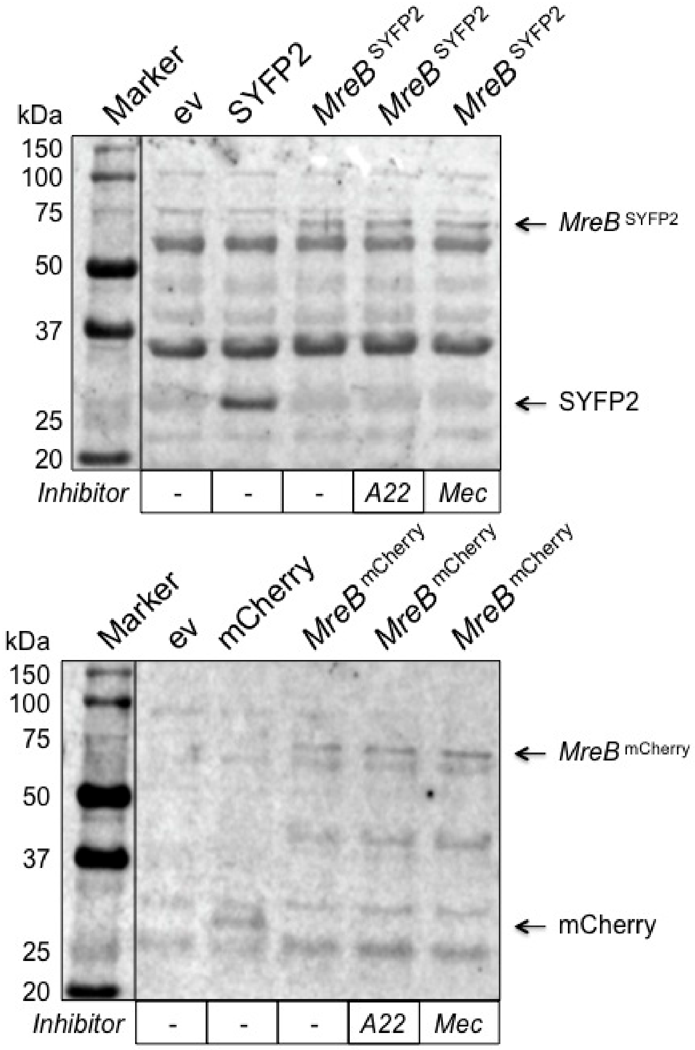

Figure S2. Degradation of the sandwich fusions is not increasing when the inhibitor is present. The MC4100 cells were grown to a steady state, expression was induced for ±6 h with 15 µM IPTG. Cells were collected by centrifugation and were prepared for SDS-PAGE and Western blotting with specific antibodies being αGFP for the top panel and αDsRed for lower panel.
