# Supplementary material for: Validation of FRET Assay for the Screening of Growth Inhibitors of Escherichia coli Reveals Elongasome Assembly Dynamics
Source: Int J Mol Sci. 2015 Jul 31;16(8):17637–54. doi: 10.3390/ijms160817637 (PMC4581212; doi:10.3390/ijms160817637)
Supplement: Supplementary file 1 [file ijms-16-17637-s001.zip › ijms-82313-Supplementary Information/ijms-82313-Supplementary 2-for publish.pptx]

## Slide 1
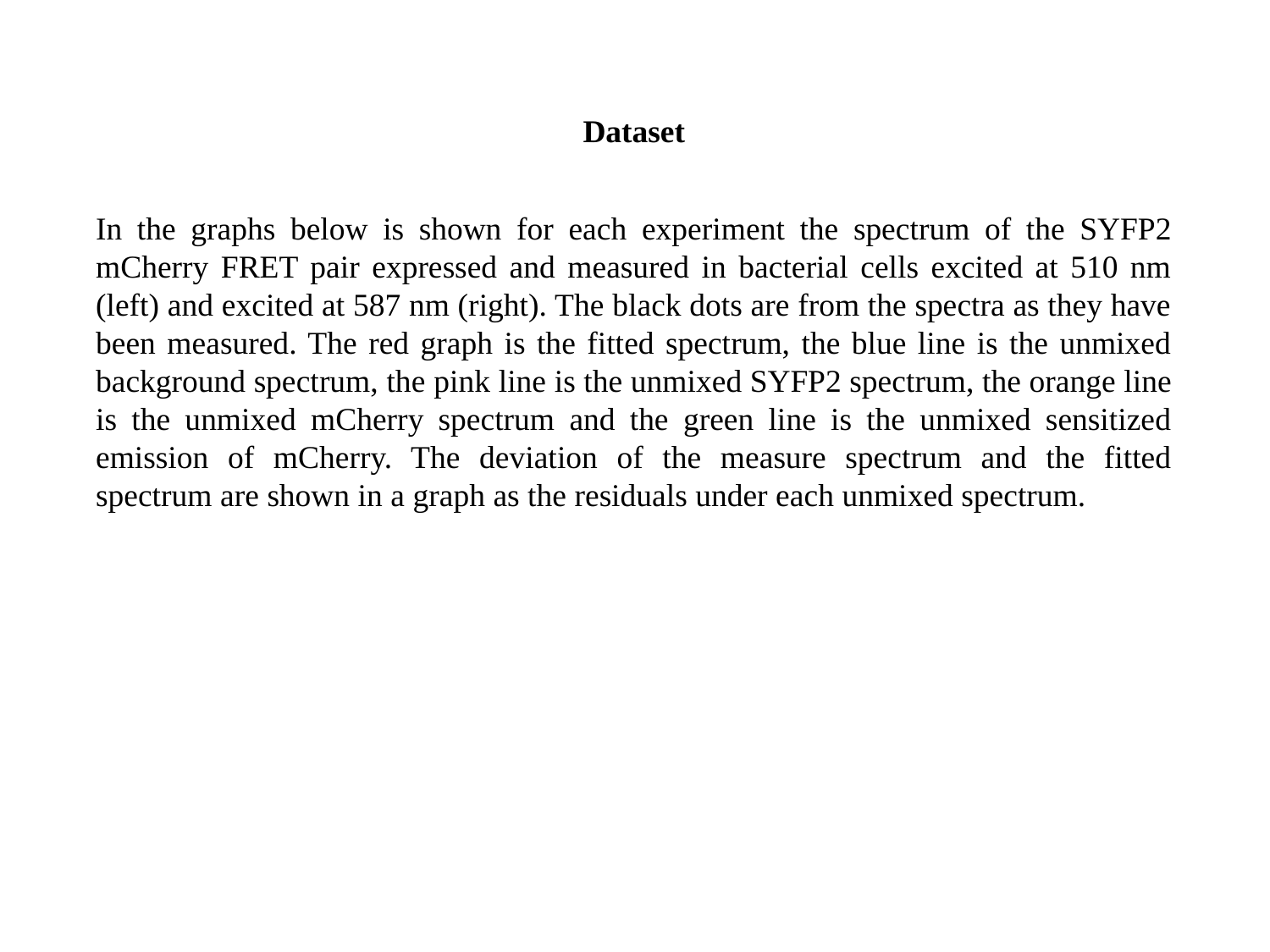

Dataset
In the graphs below is shown for each experiment the spectrum of the SYFP2 mCherry FRET pair expressed and measured in bacterial cells excited at 510 nm (left) and excited at 587 nm (right). The black dots are from the spectra as they have been measured. The red graph is the fitted spectrum, the blue line is the unmixed background spectrum, the pink line is the unmixed SYFP2 spectrum, the orange line is the unmixed mCherry spectrum and the green line is the unmixed sensitized emission of mCherry. The deviation of the measure spectrum and the fitted spectrum are shown in a graph as the residuals under each unmixed spectrum.

## Slide 2
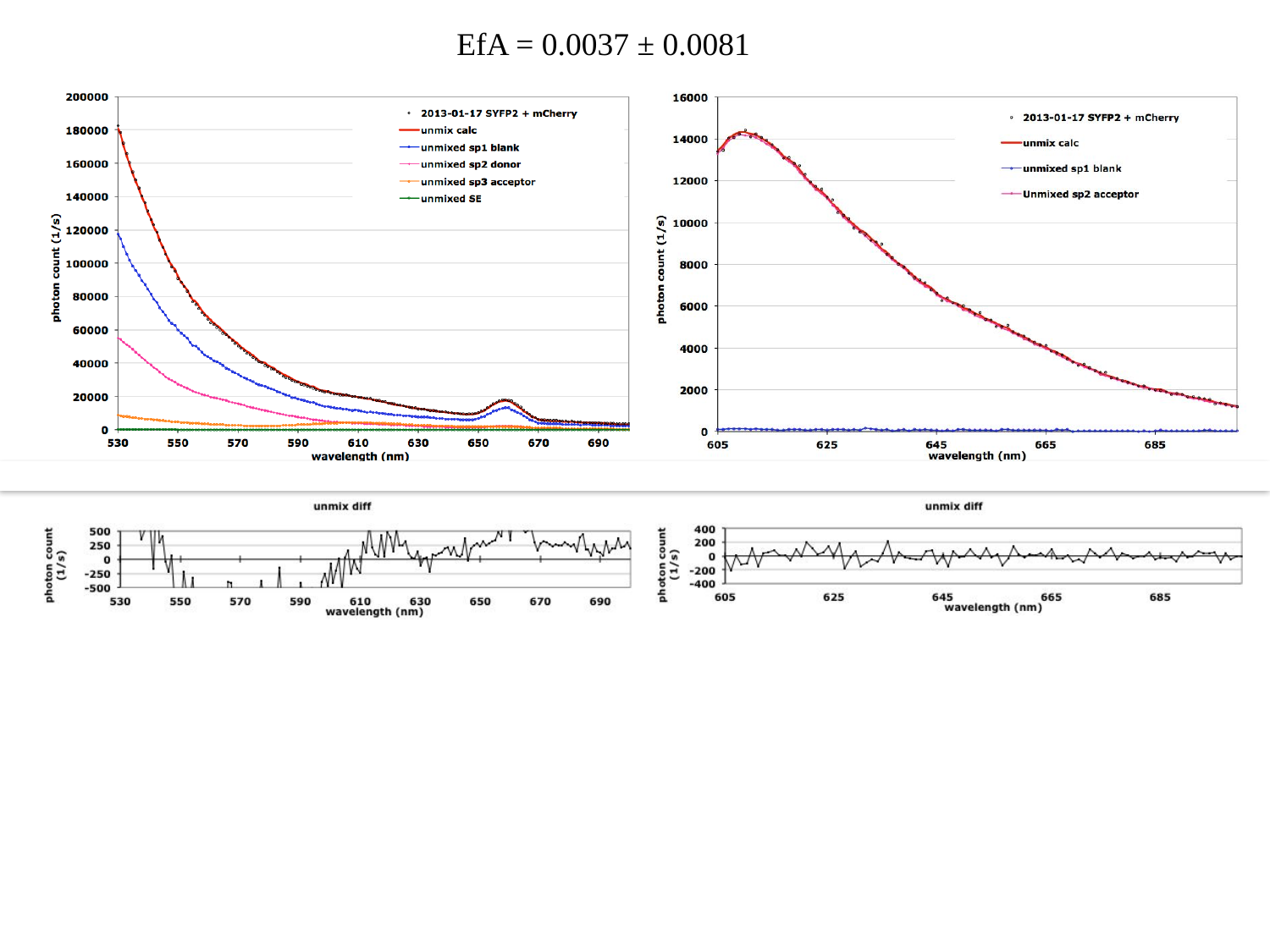

EfA = 0.0037 ± 0.0081

## Slide 3
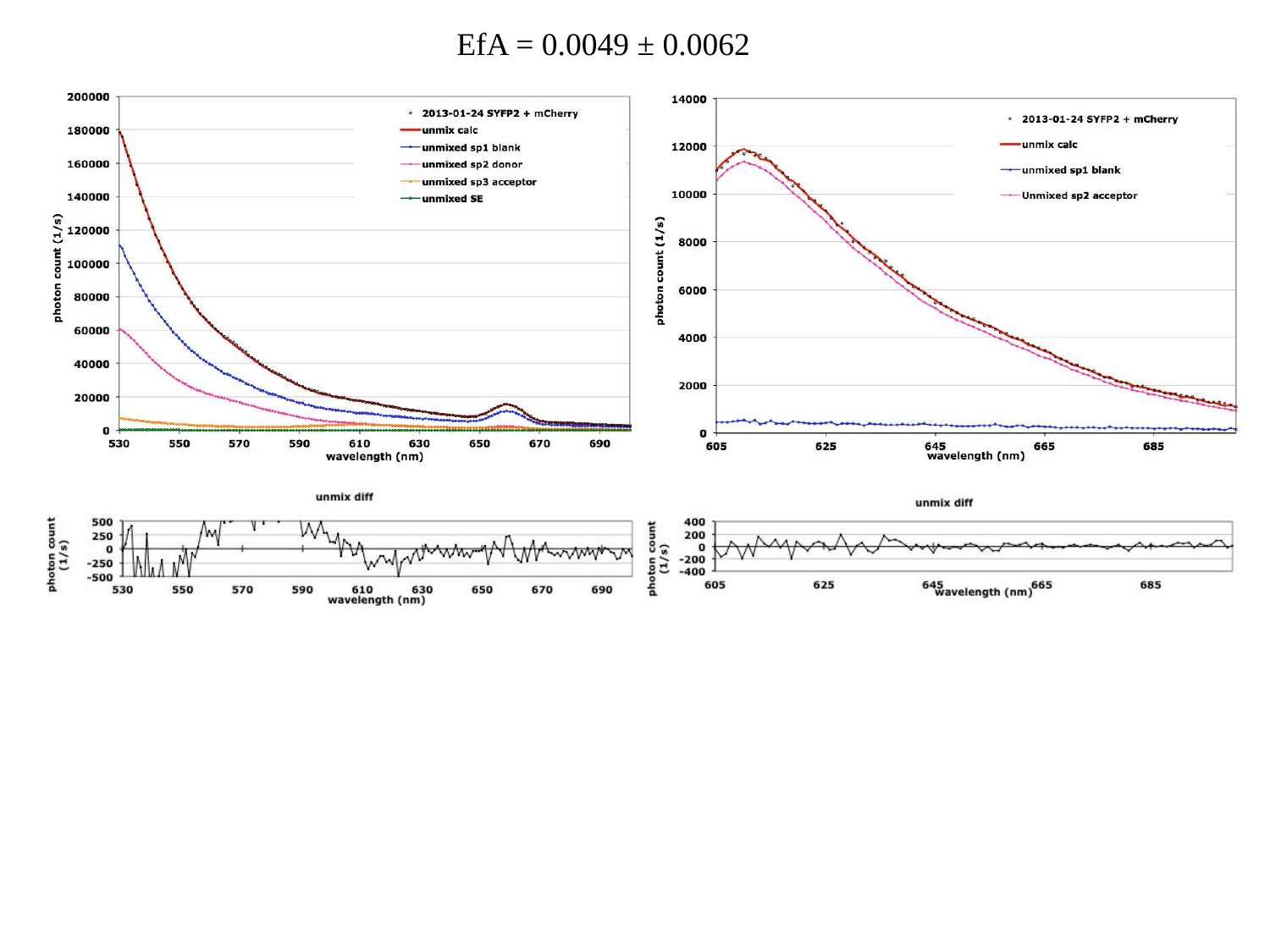

EfA = 0.0049 ± 0.0062

## Slide 4
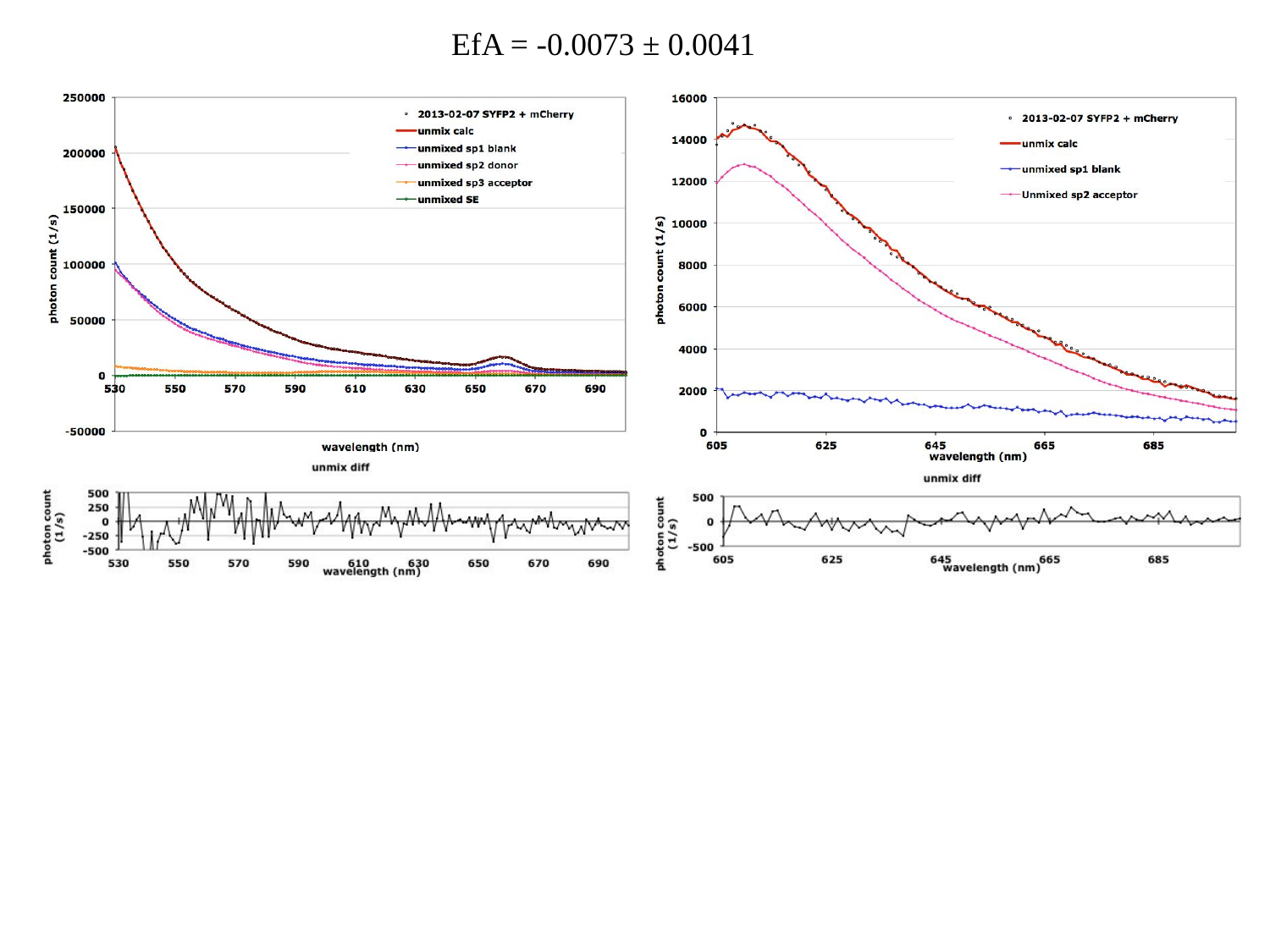

EfA = -0.0073 ± 0.0041

## Slide 5
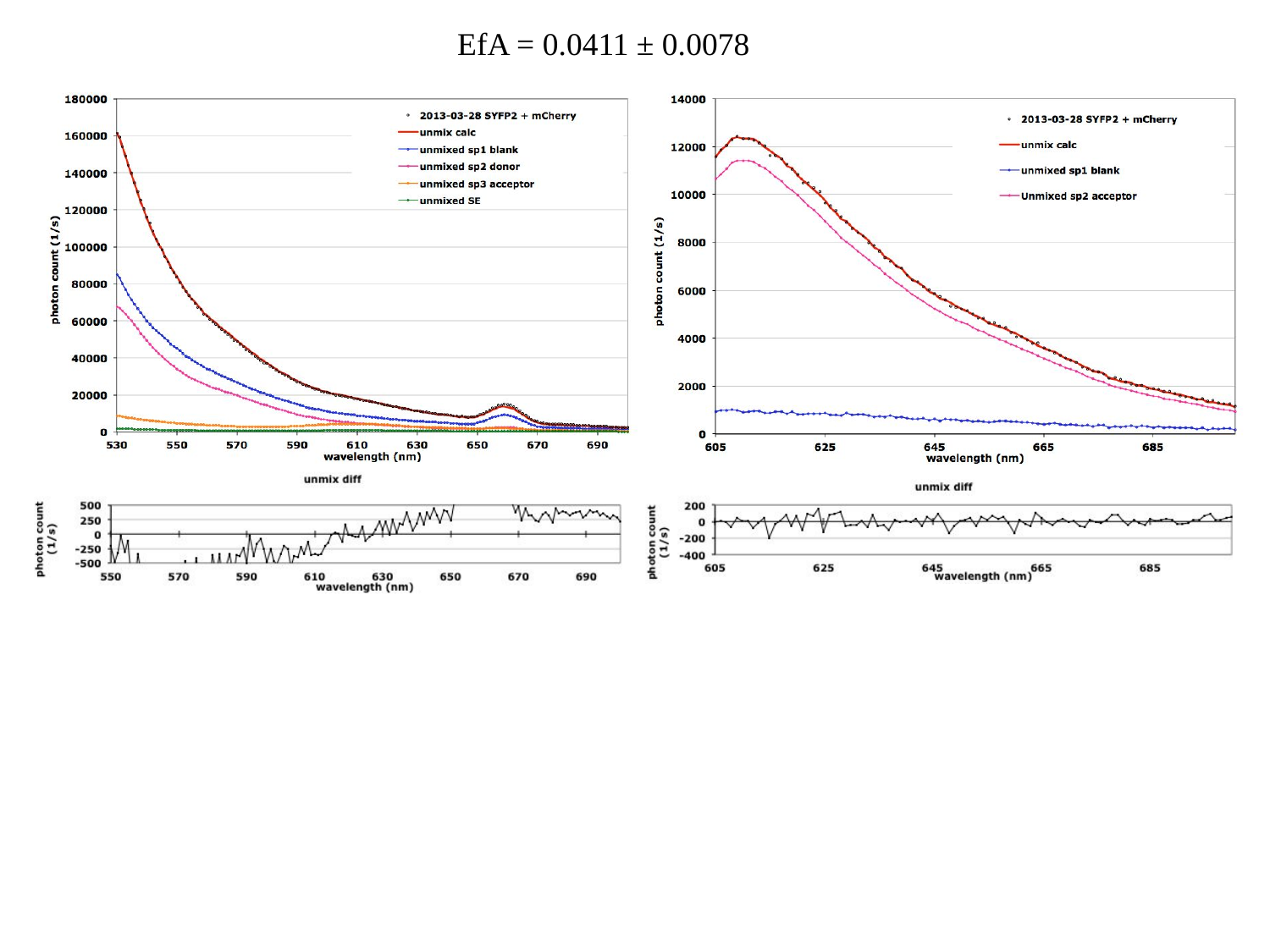

EfA = 0.0411 ± 0.0078

## Slide 6
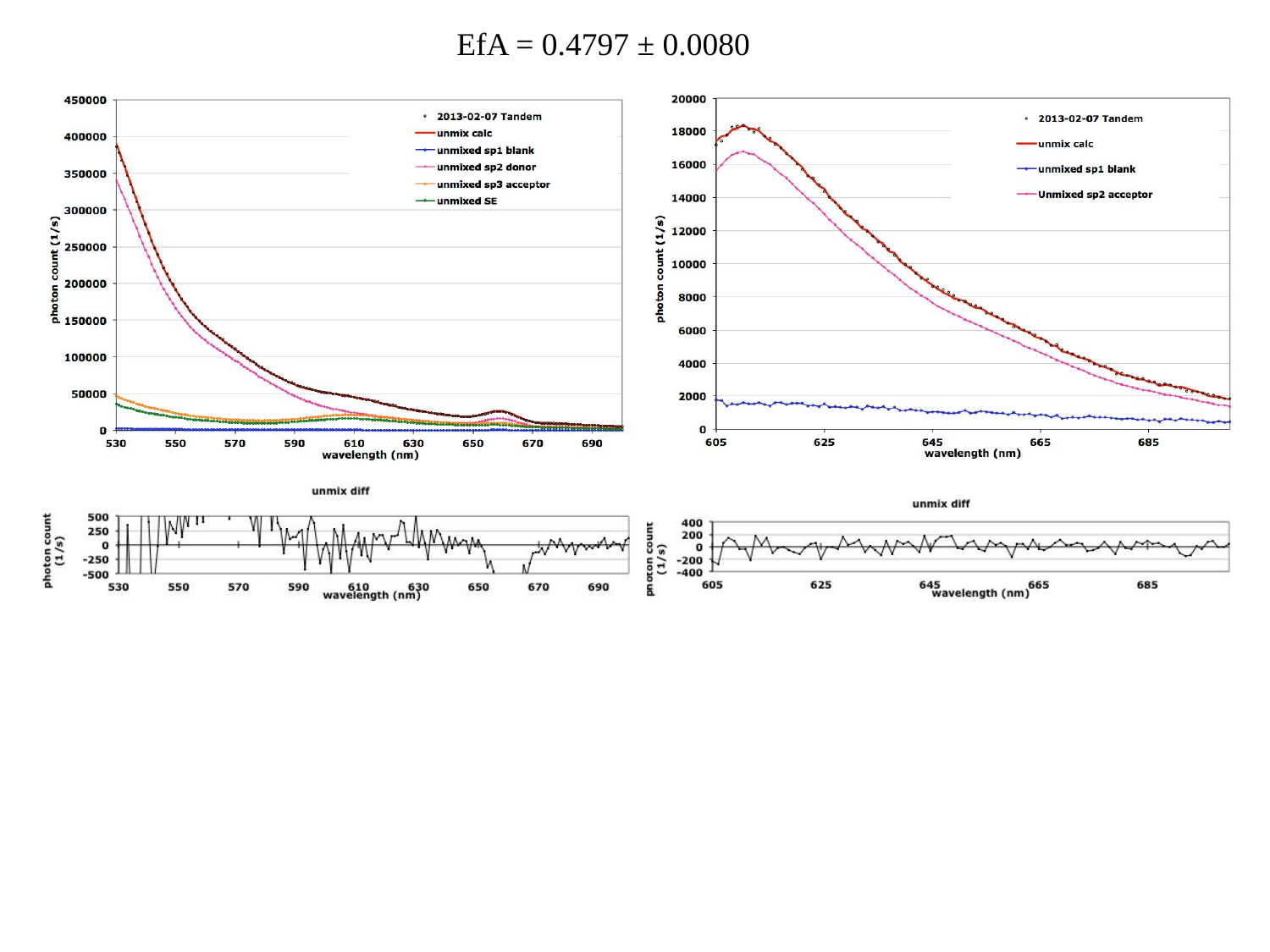

EfA = 0.4797 ± 0.0080

## Slide 7
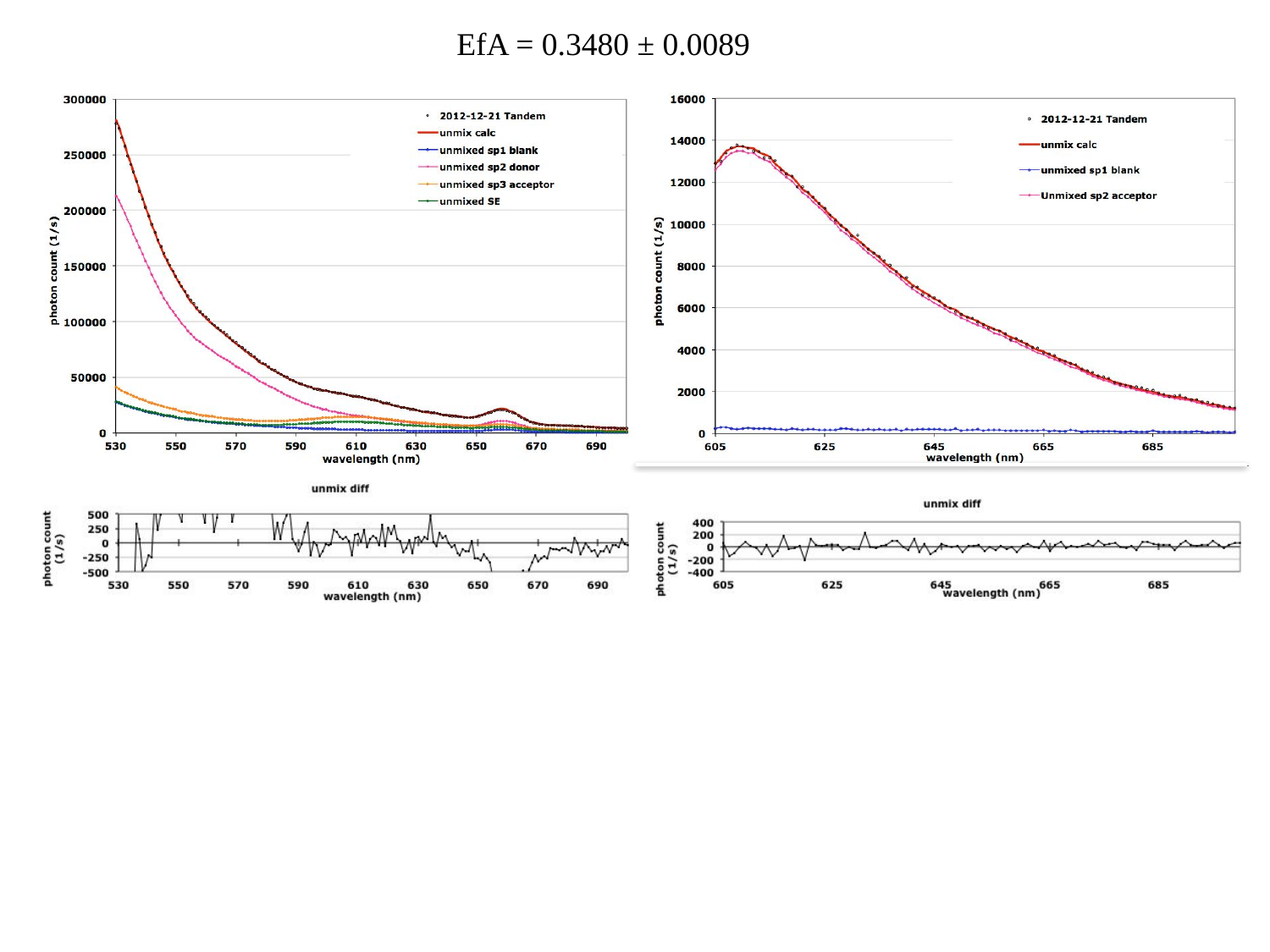

EfA = 0.3480 ± 0.0089

## Slide 8
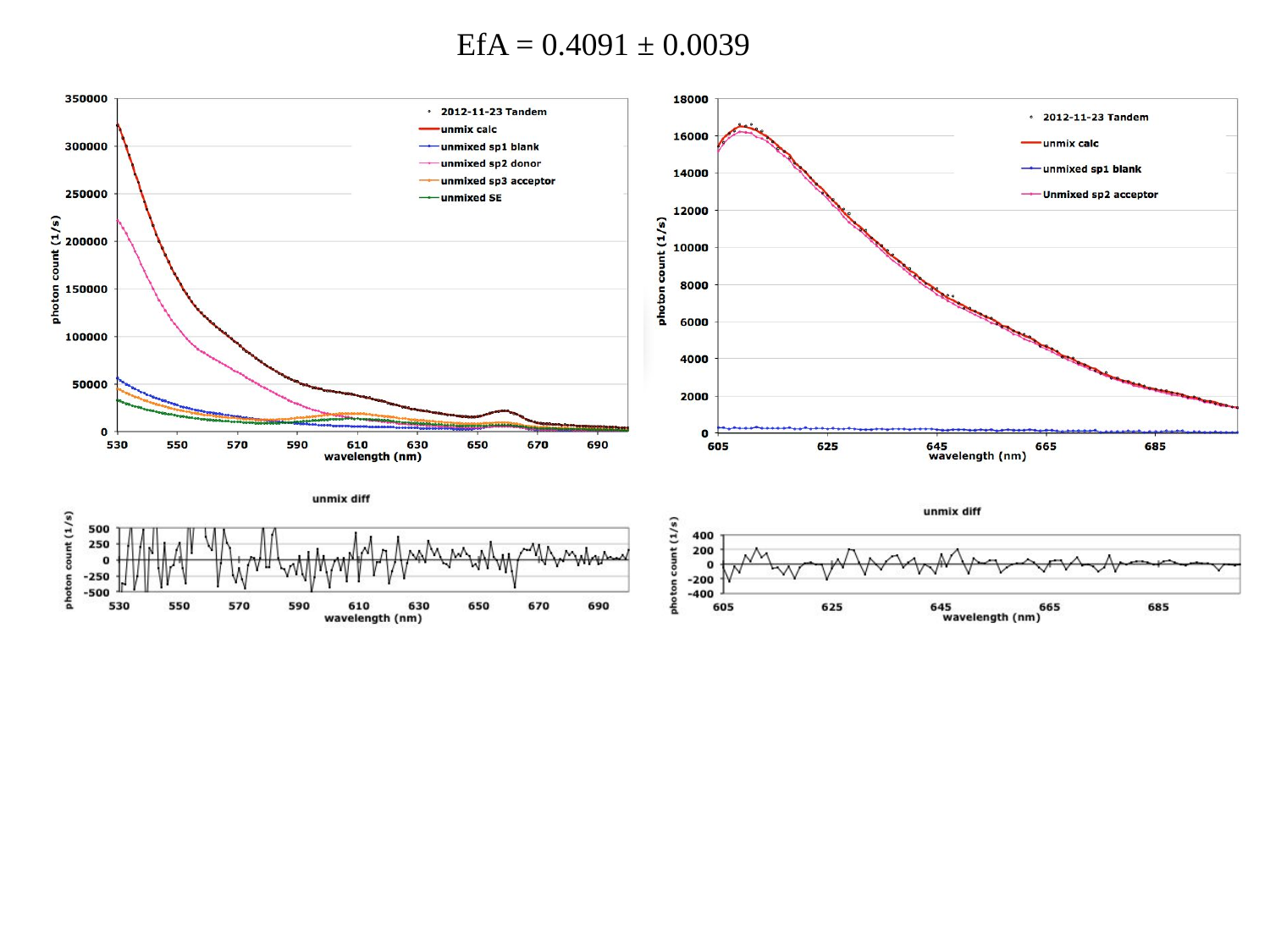

EfA = 0.4091 ± 0.0039

## Slide 9
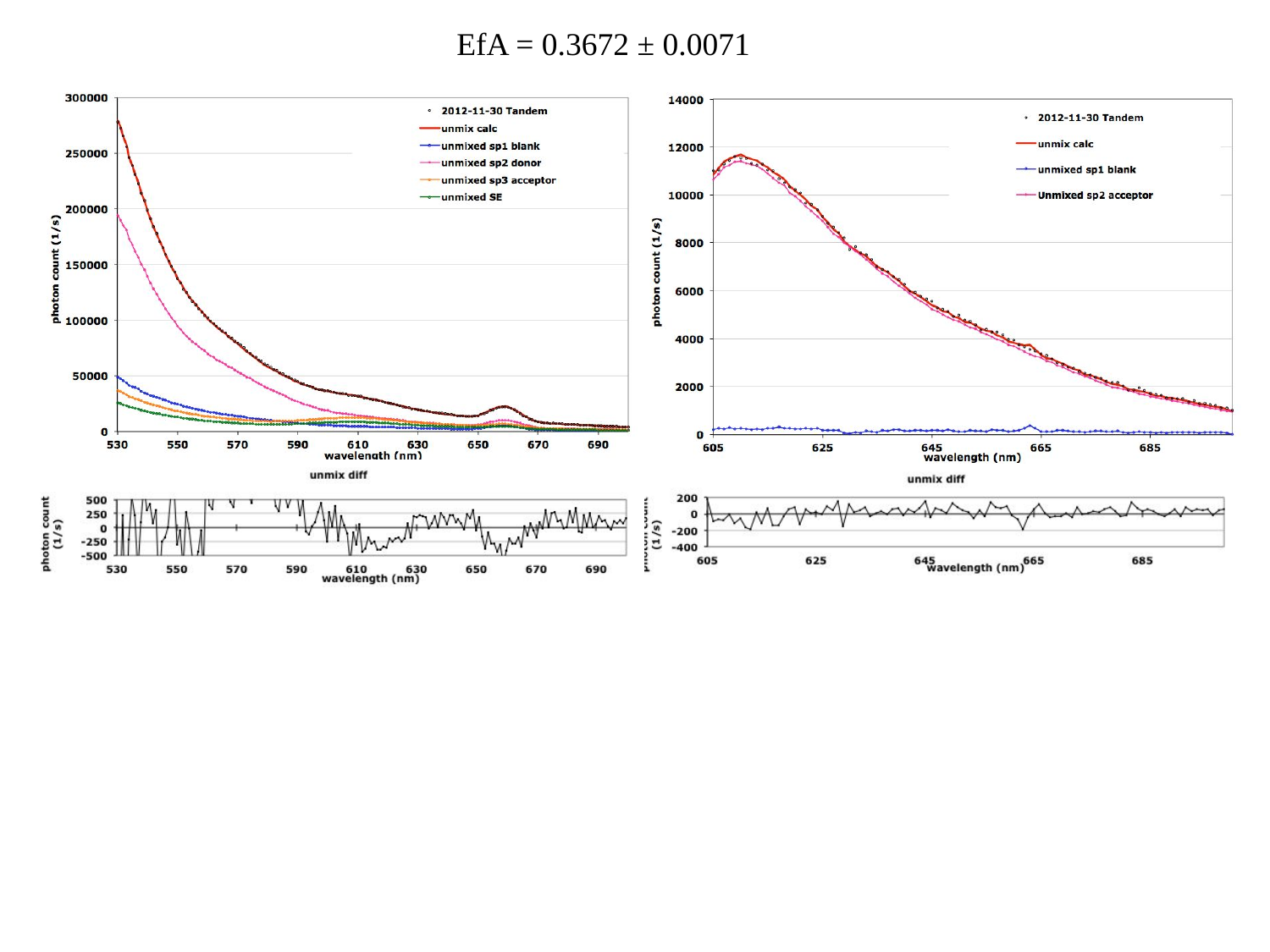

EfA = 0.3672 ± 0.0071

## Slide 10
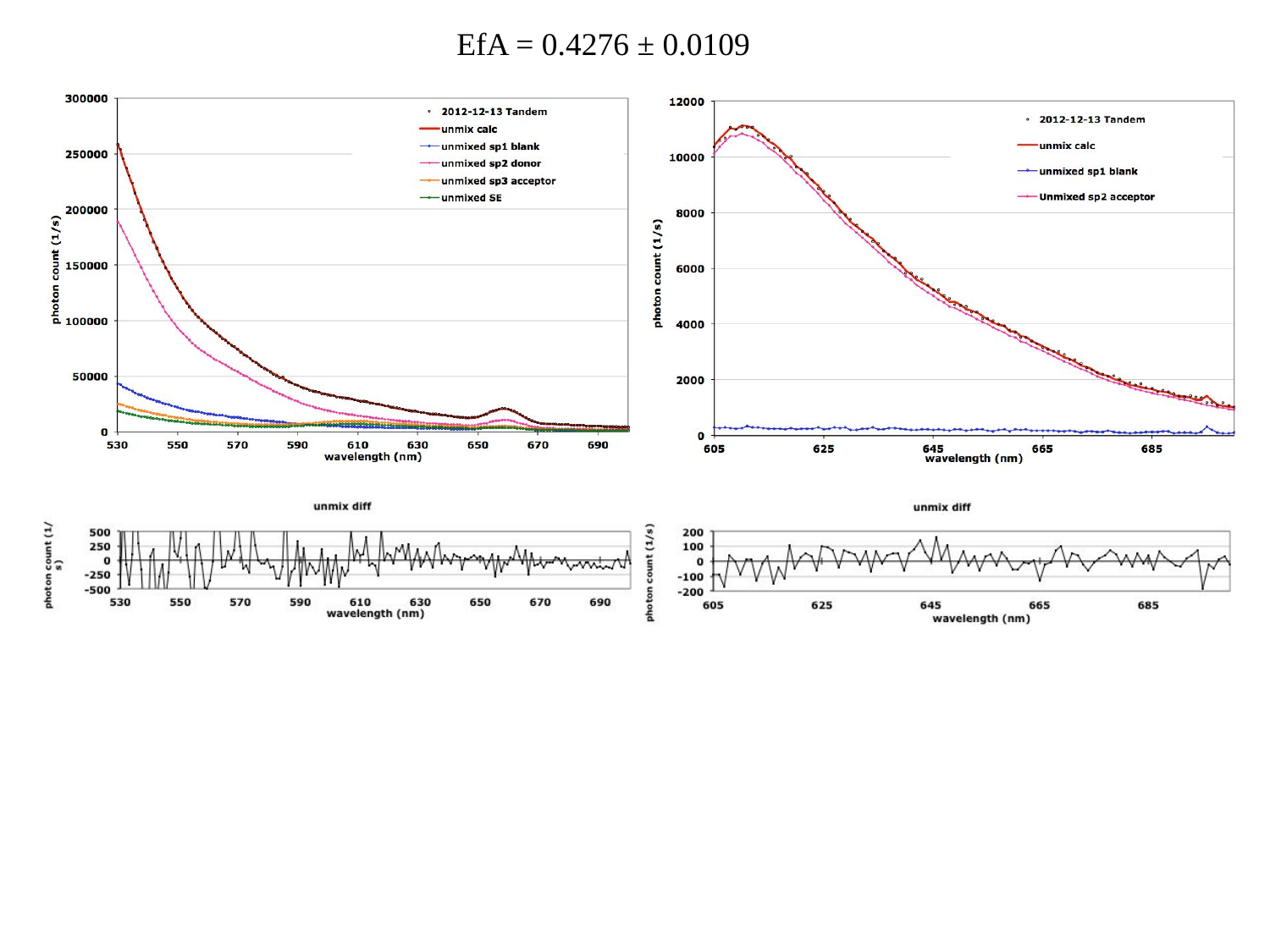

EfA = 0.4276 ± 0.0109

## Slide 11
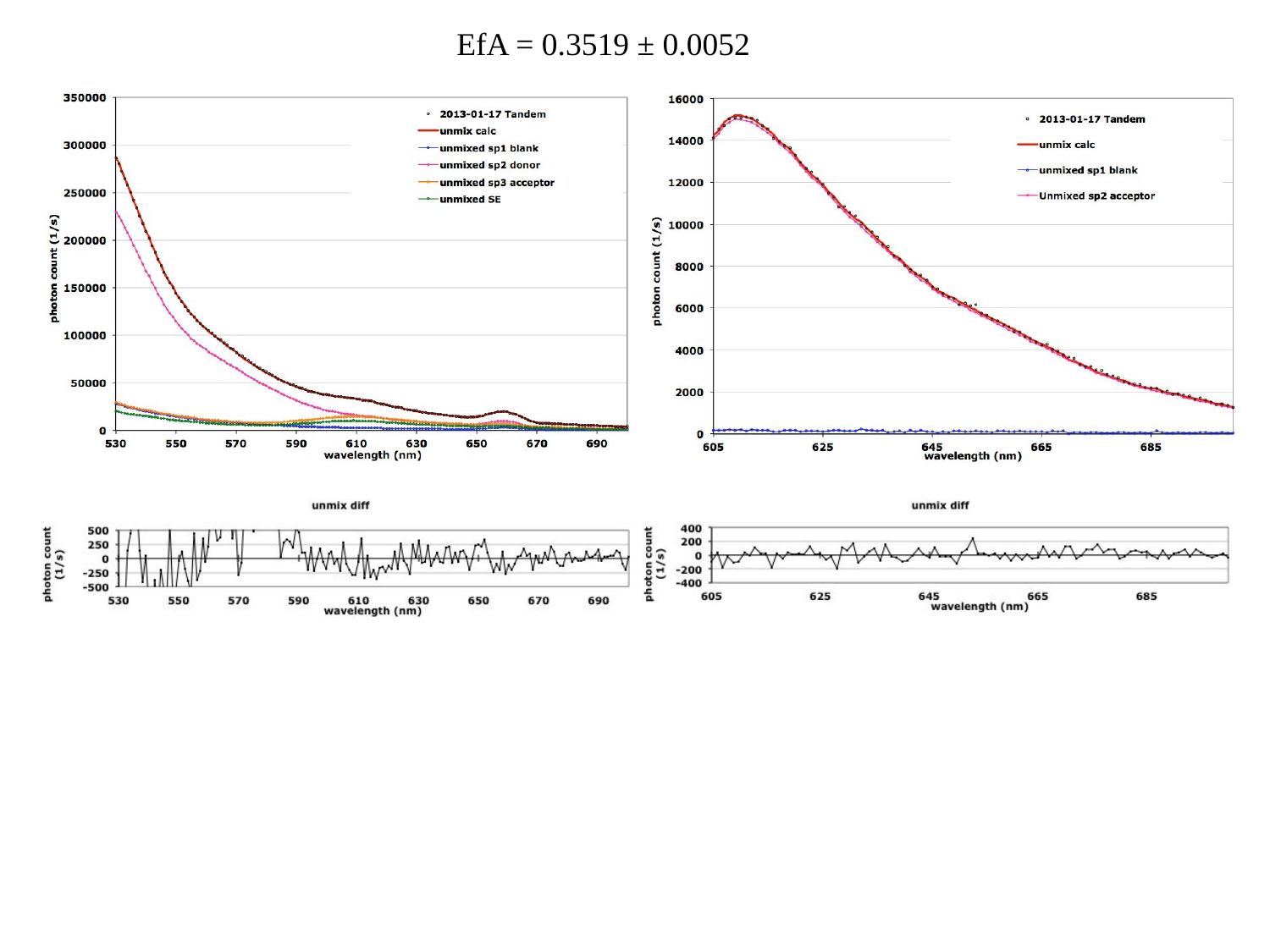

EfA = 0.3519 ± 0.0052

## Slide 12
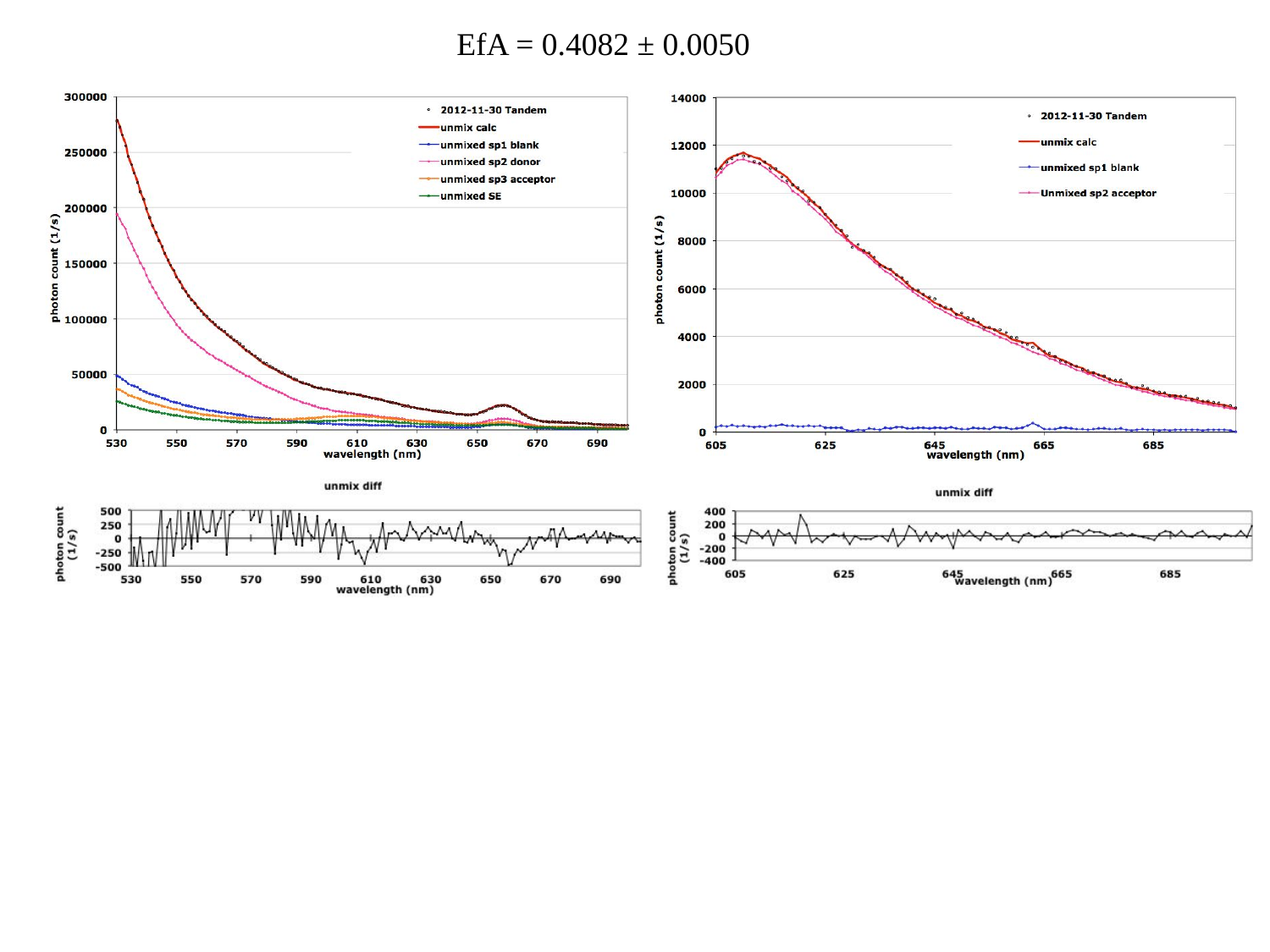

EfA = 0.4082 ± 0.0050

## Slide 13
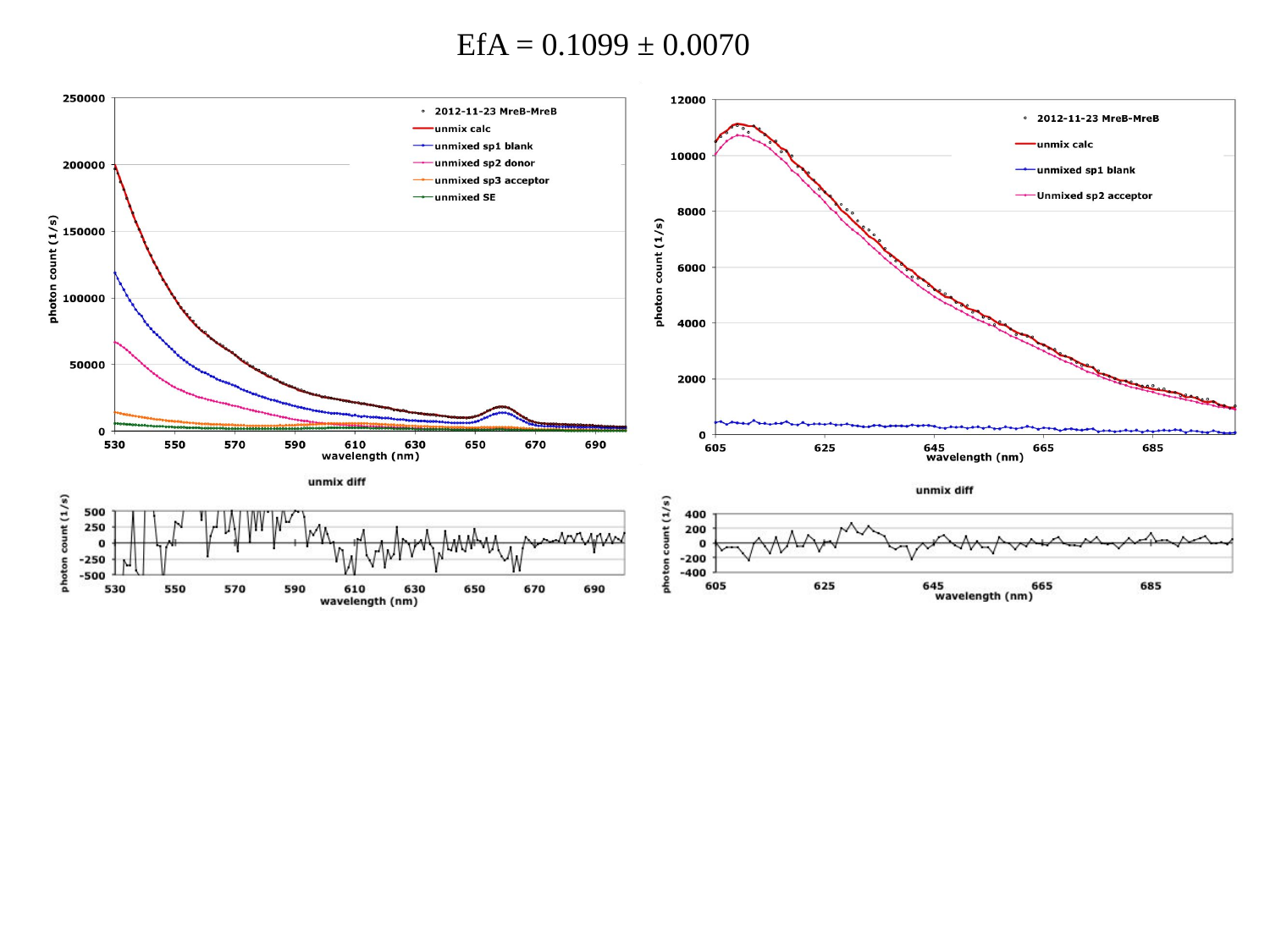

EfA = 0.1099 ± 0.0070

## Slide 14
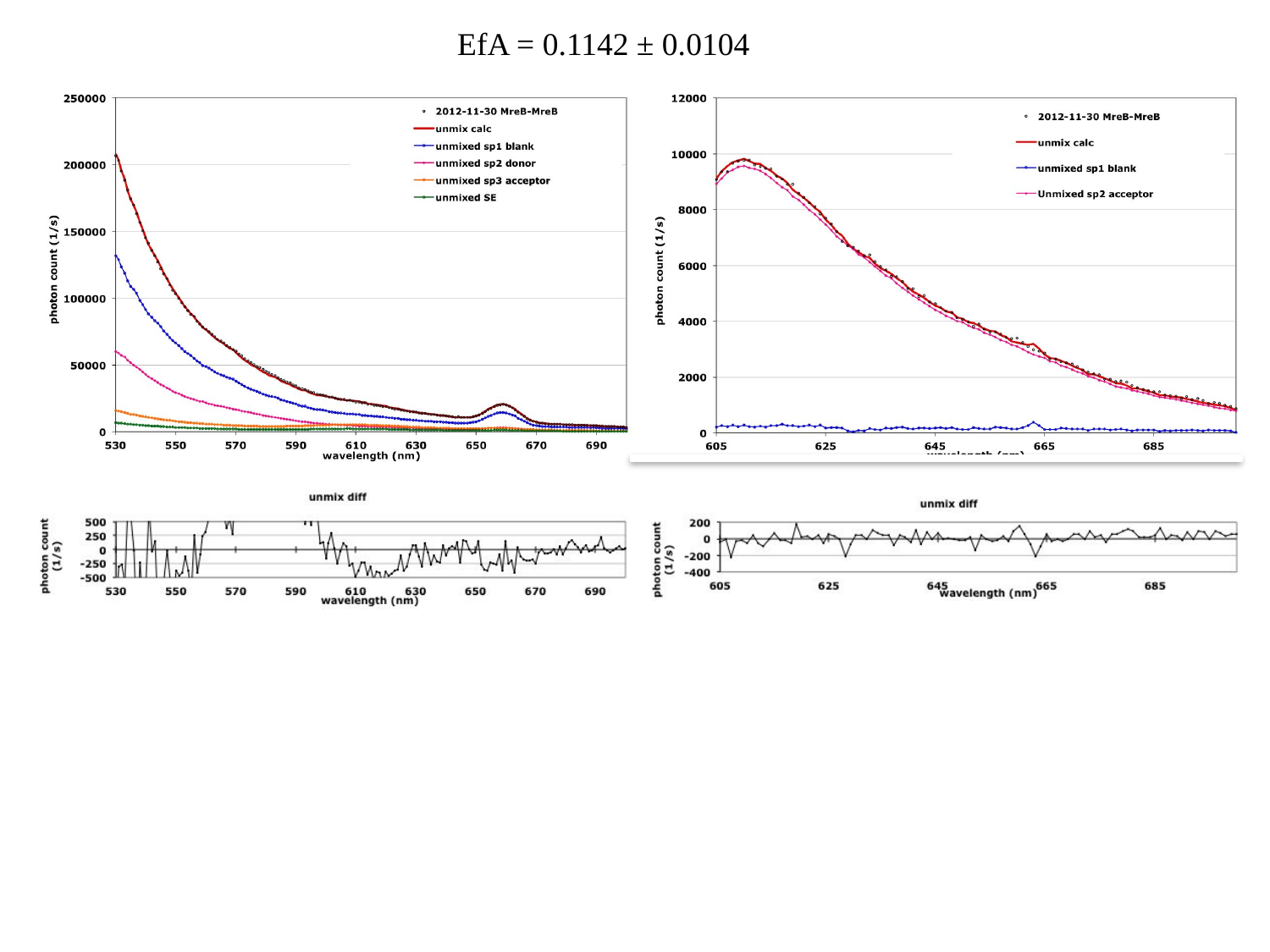

EfA = 0.1142 ± 0.0104

## Slide 15
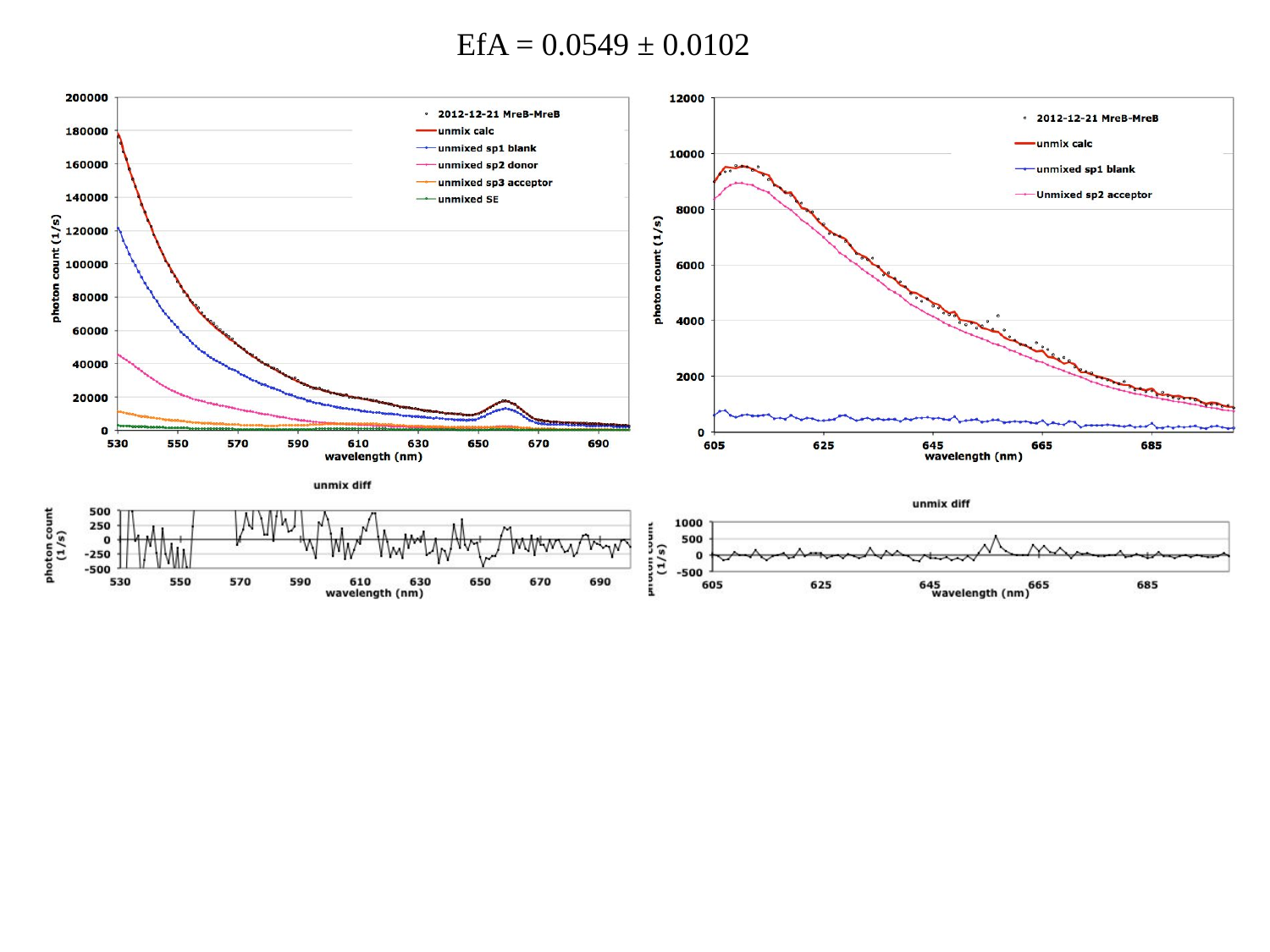

EfA = 0.0549 ± 0.0102

## Slide 16
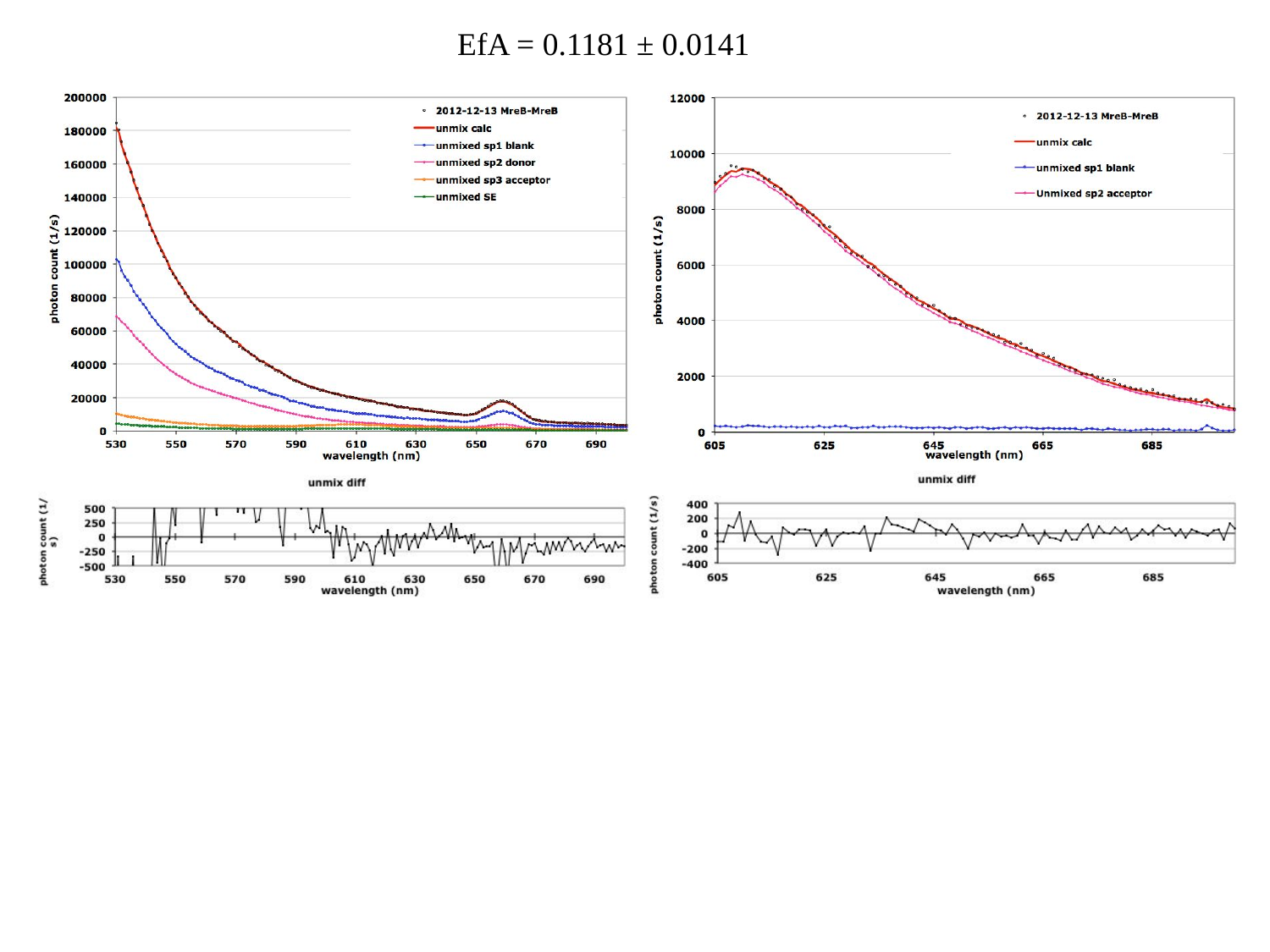

EfA = 0.1181 ± 0.0141

## Slide 17
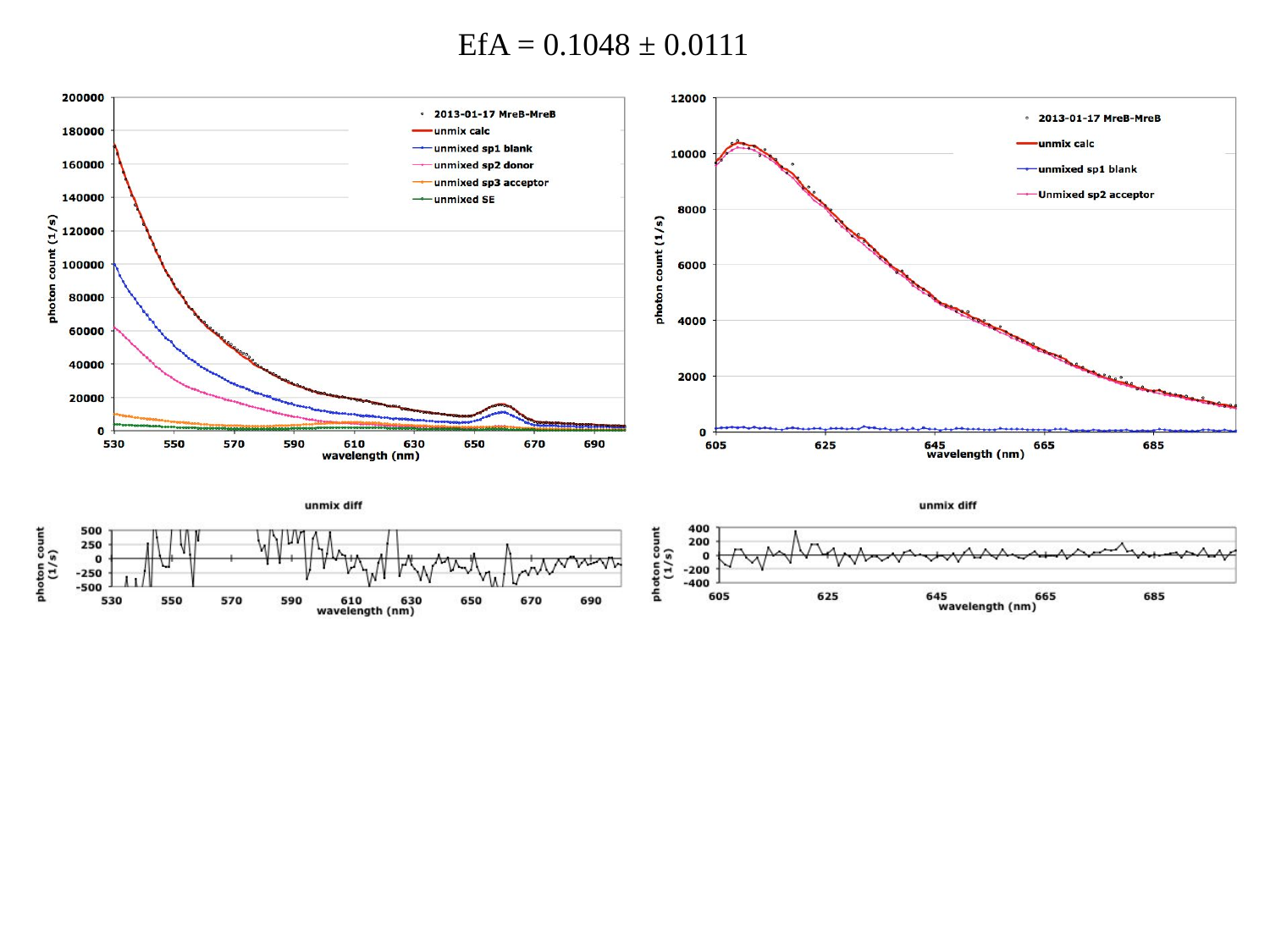

EfA = 0.1048 ± 0.0111

## Slide 18
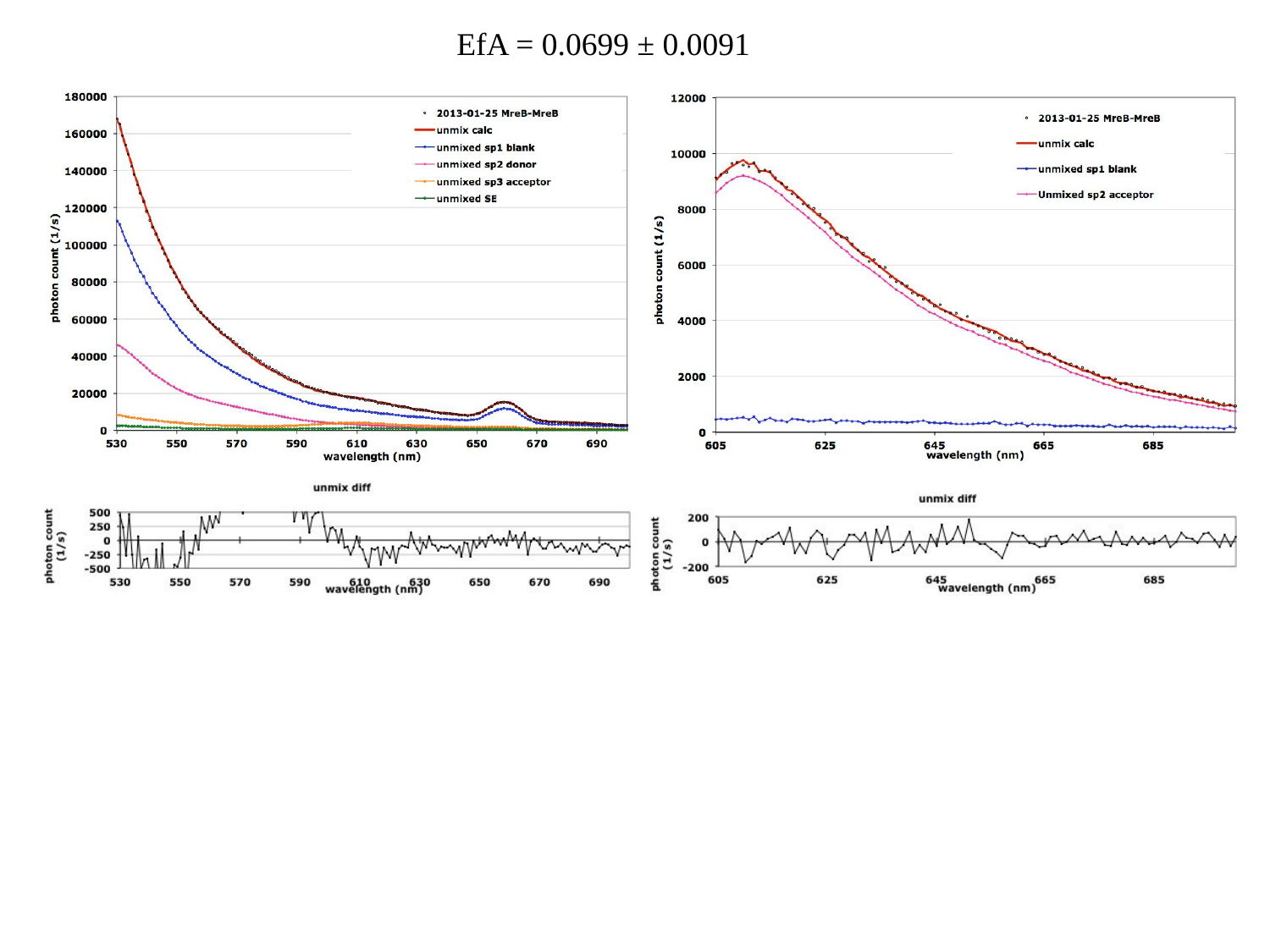

EfA = 0.0699 ± 0.0091

## Slide 19
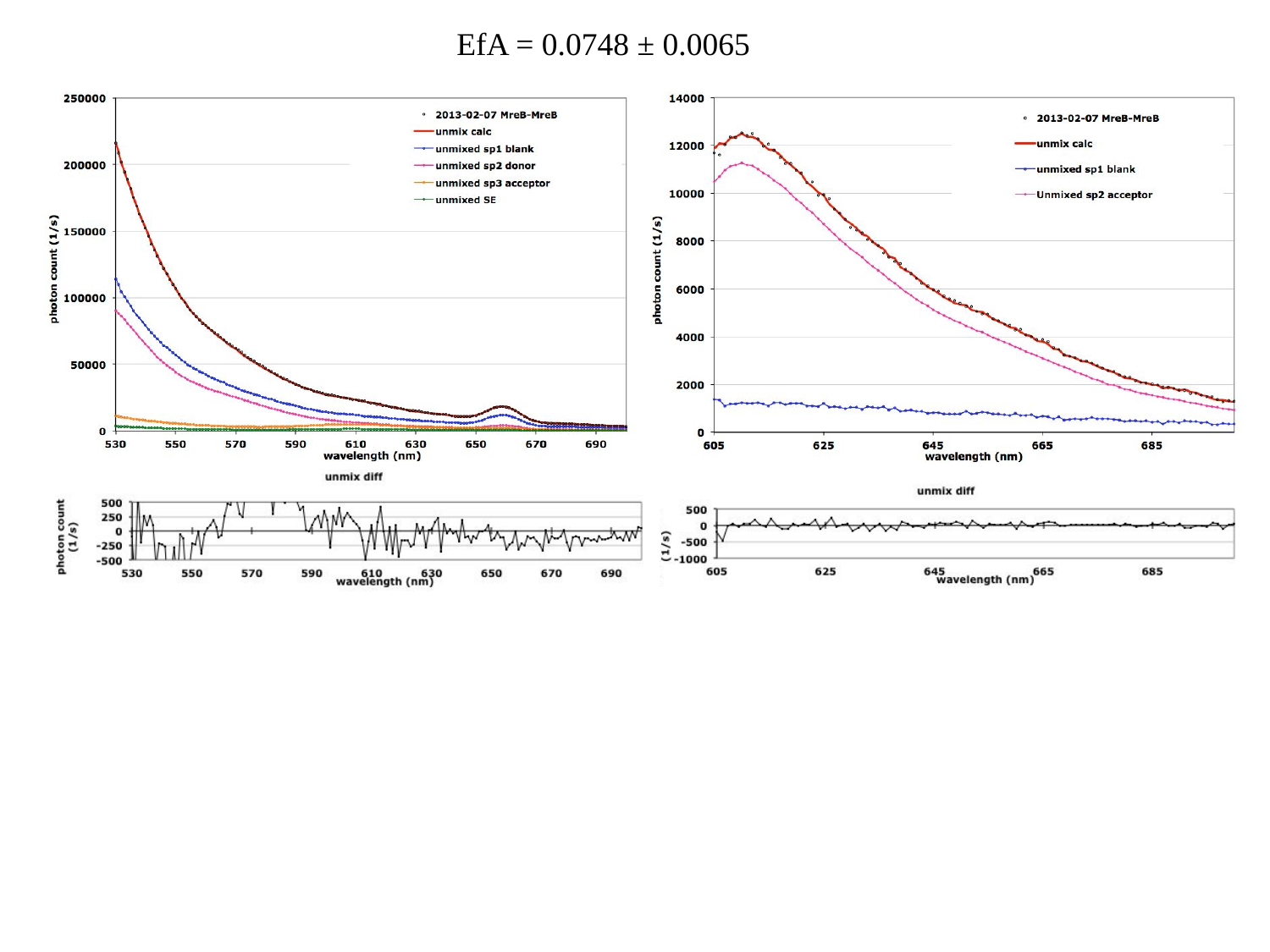

EfA = 0.0748 ± 0.0065

## Slide 20
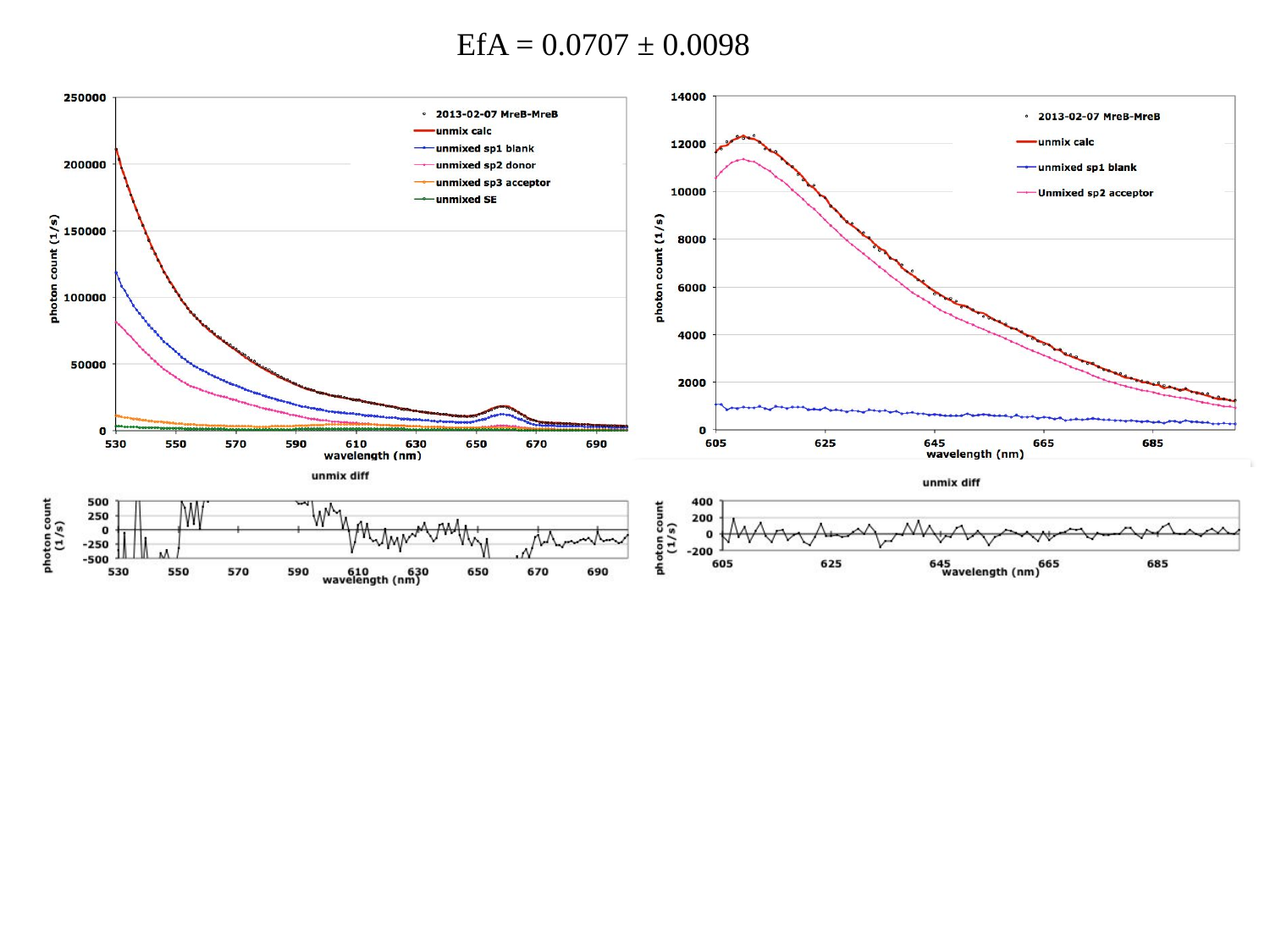

EfA = 0.0707 ± 0.0098

## Slide 21
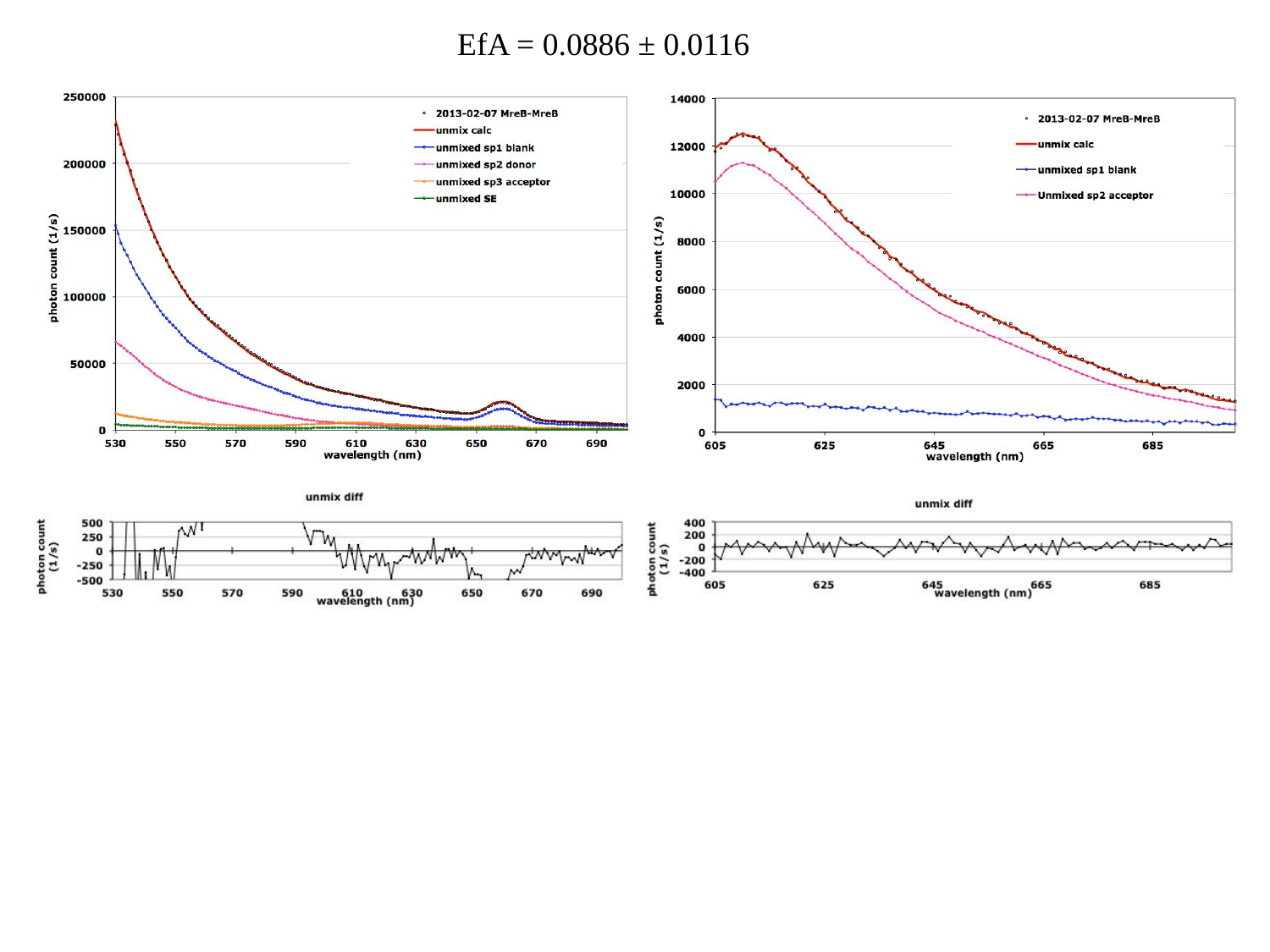

EfA = 0.0886 ± 0.0116

## Slide 22
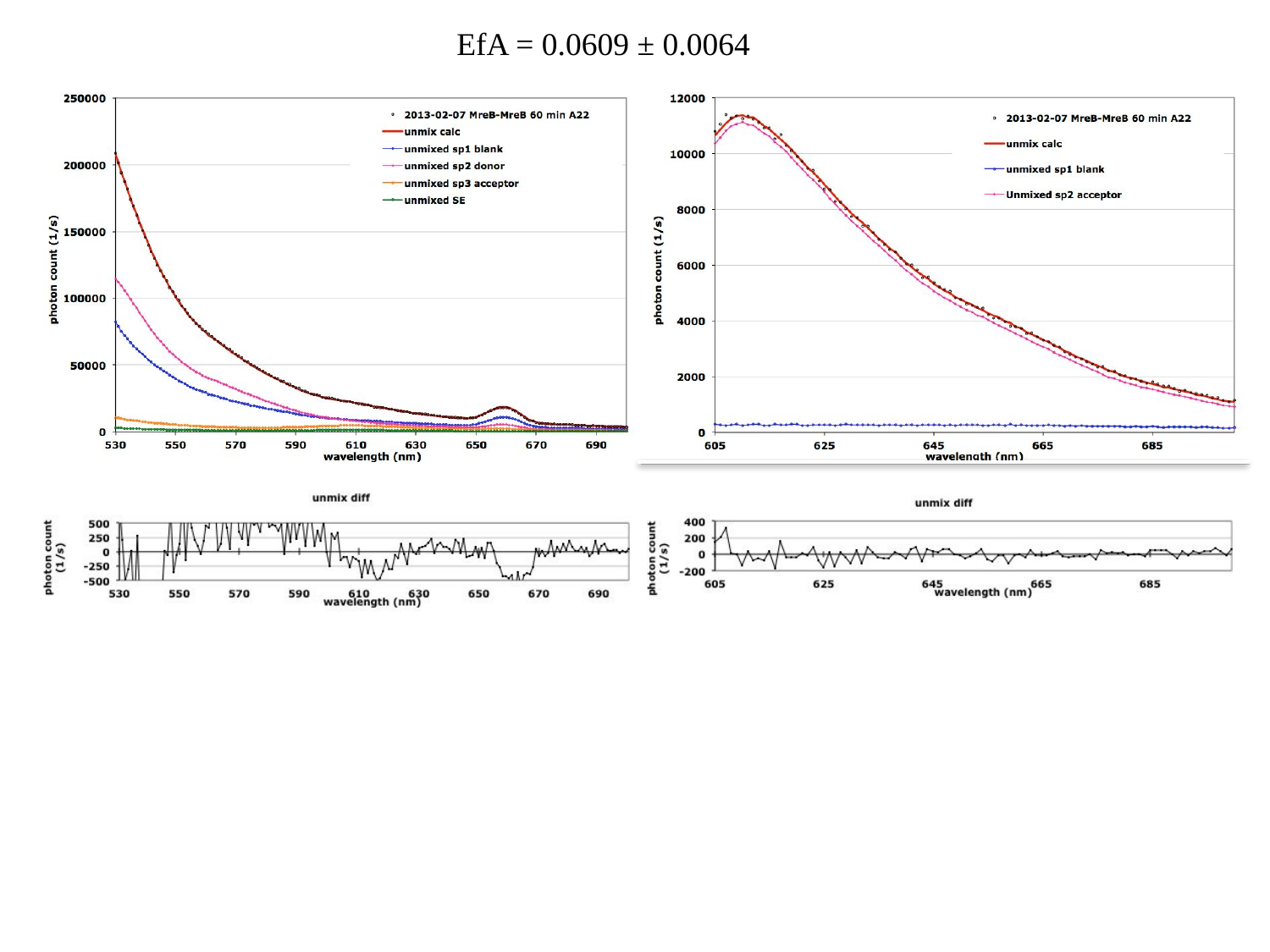

EfA = 0.0609 ± 0.0064

## Slide 23
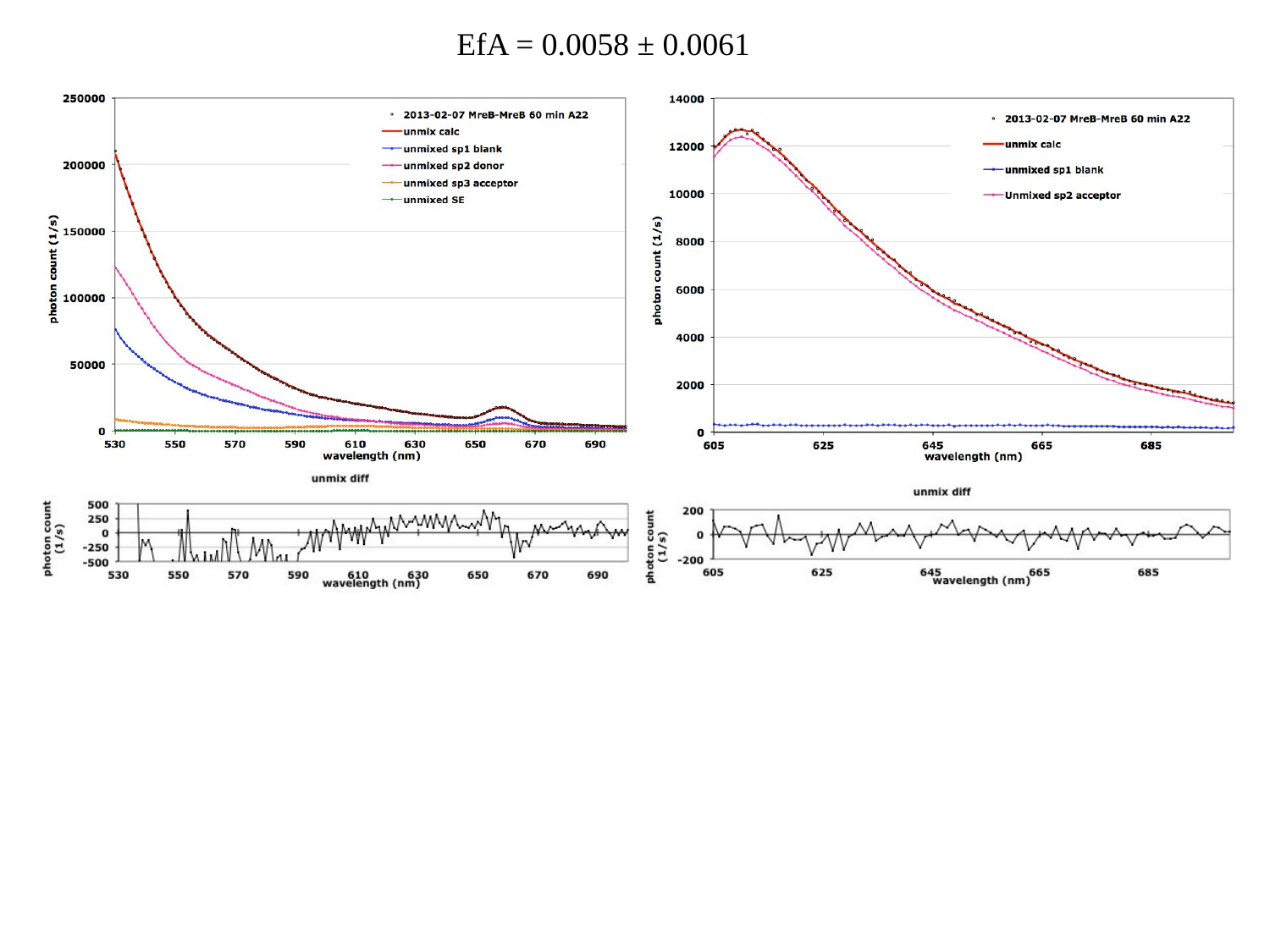

EfA = 0.0058 ± 0.0061

## Slide 24
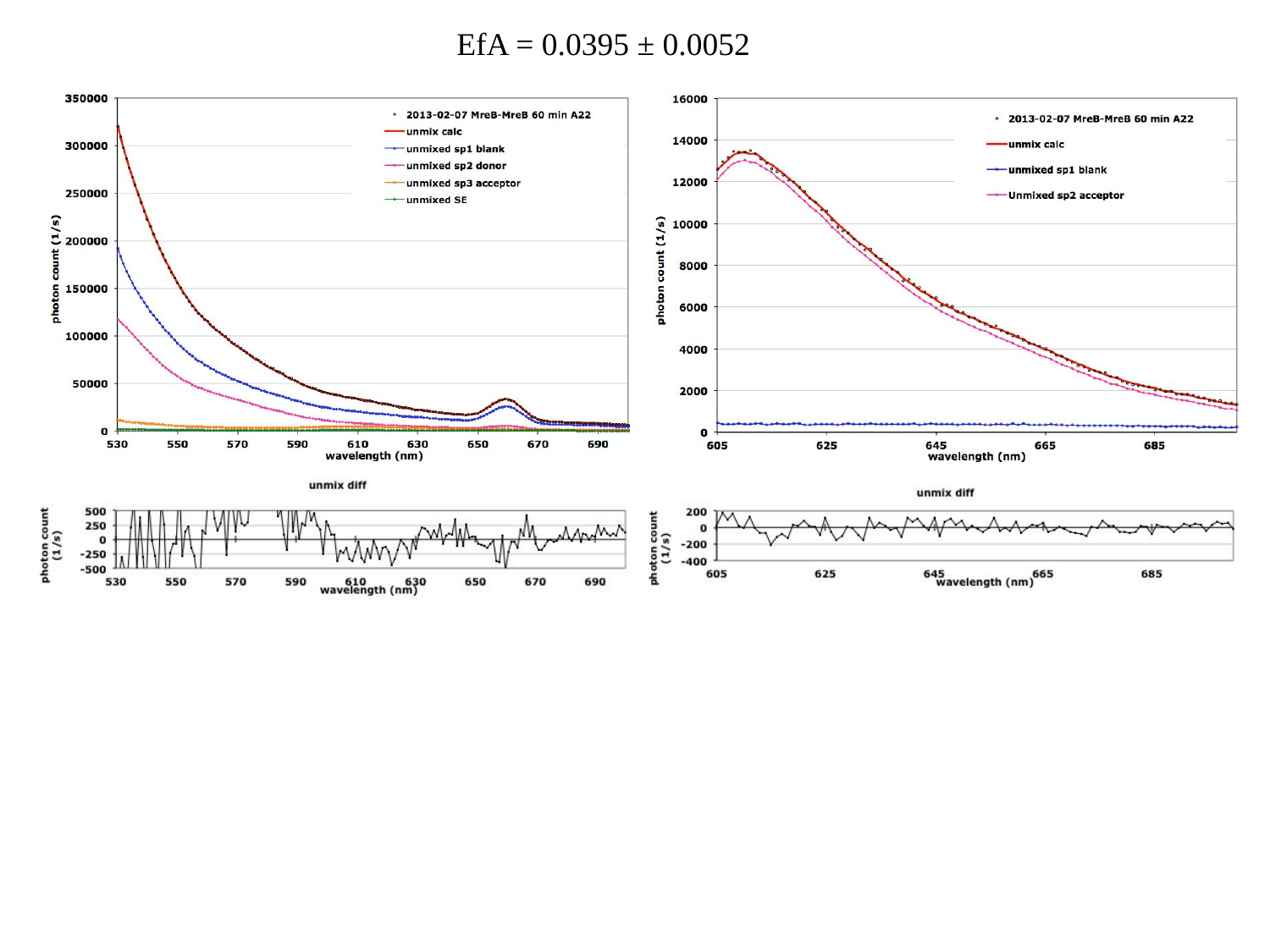

EfA = 0.0395 ± 0.0052

## Slide 25
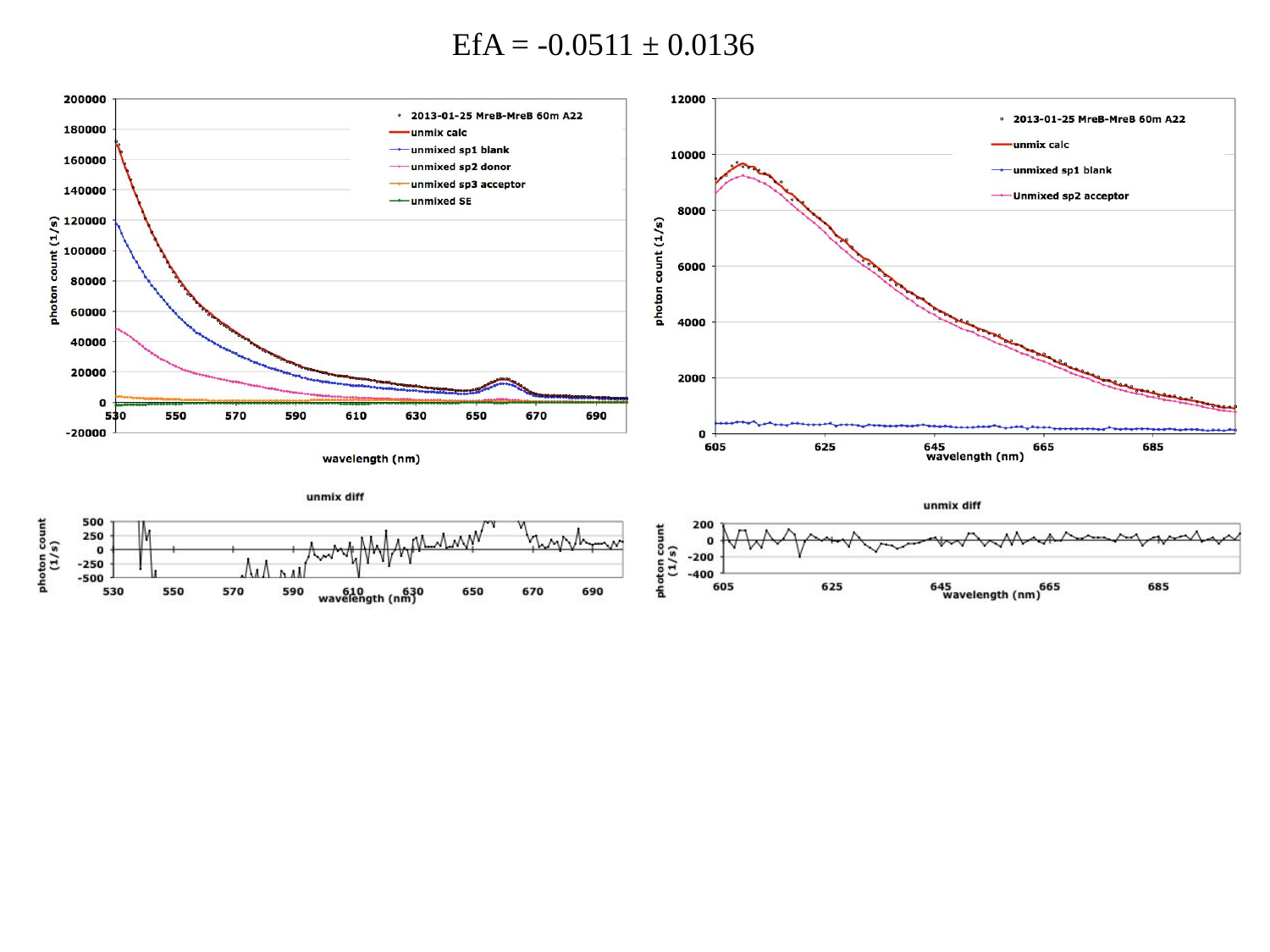

EfA = -0.0511 ± 0.0136

## Slide 26
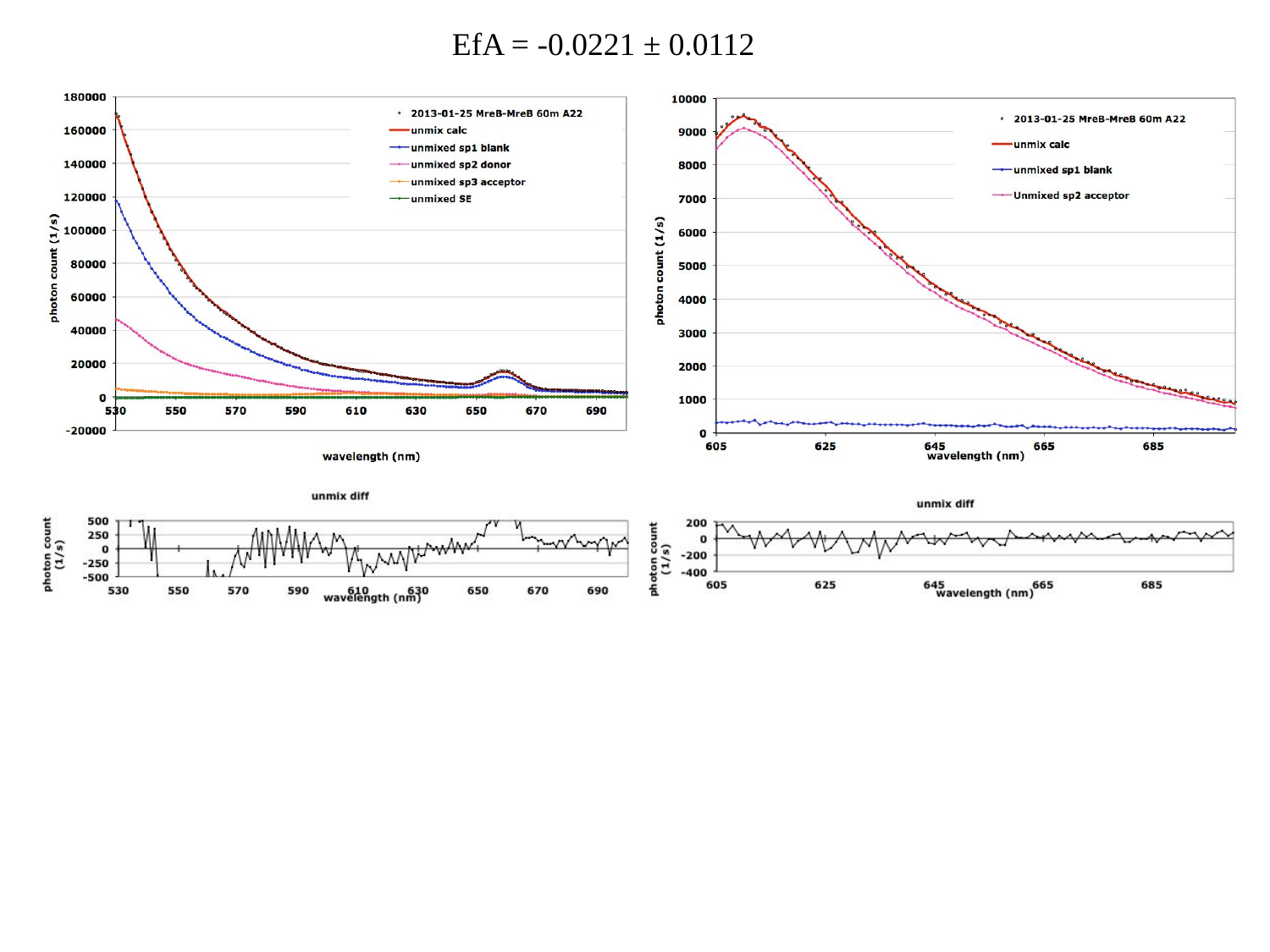

EfA = -0.0221 ± 0.0112

## Slide 27
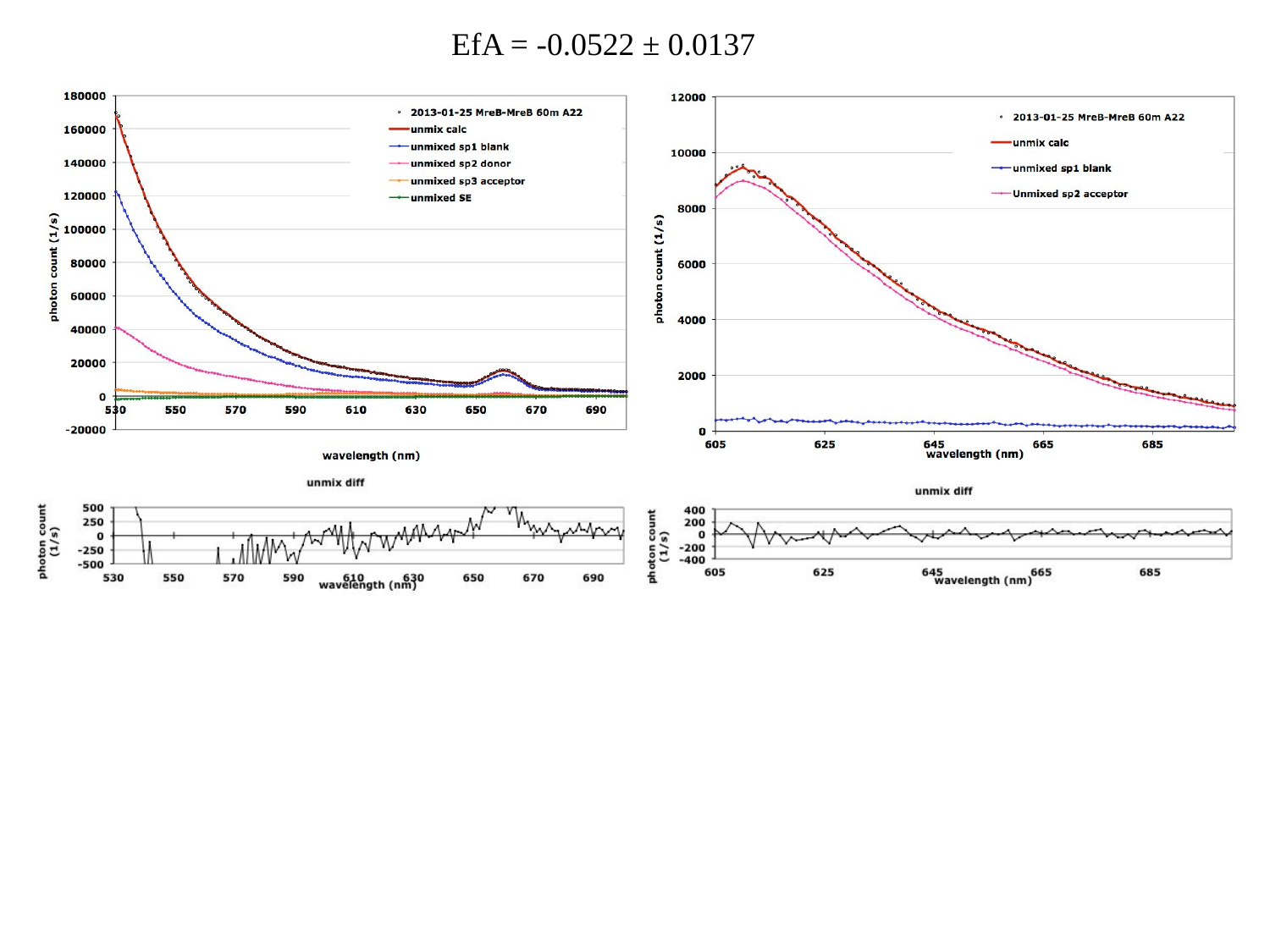

EfA = -0.0522 ± 0.0137

## Slide 28
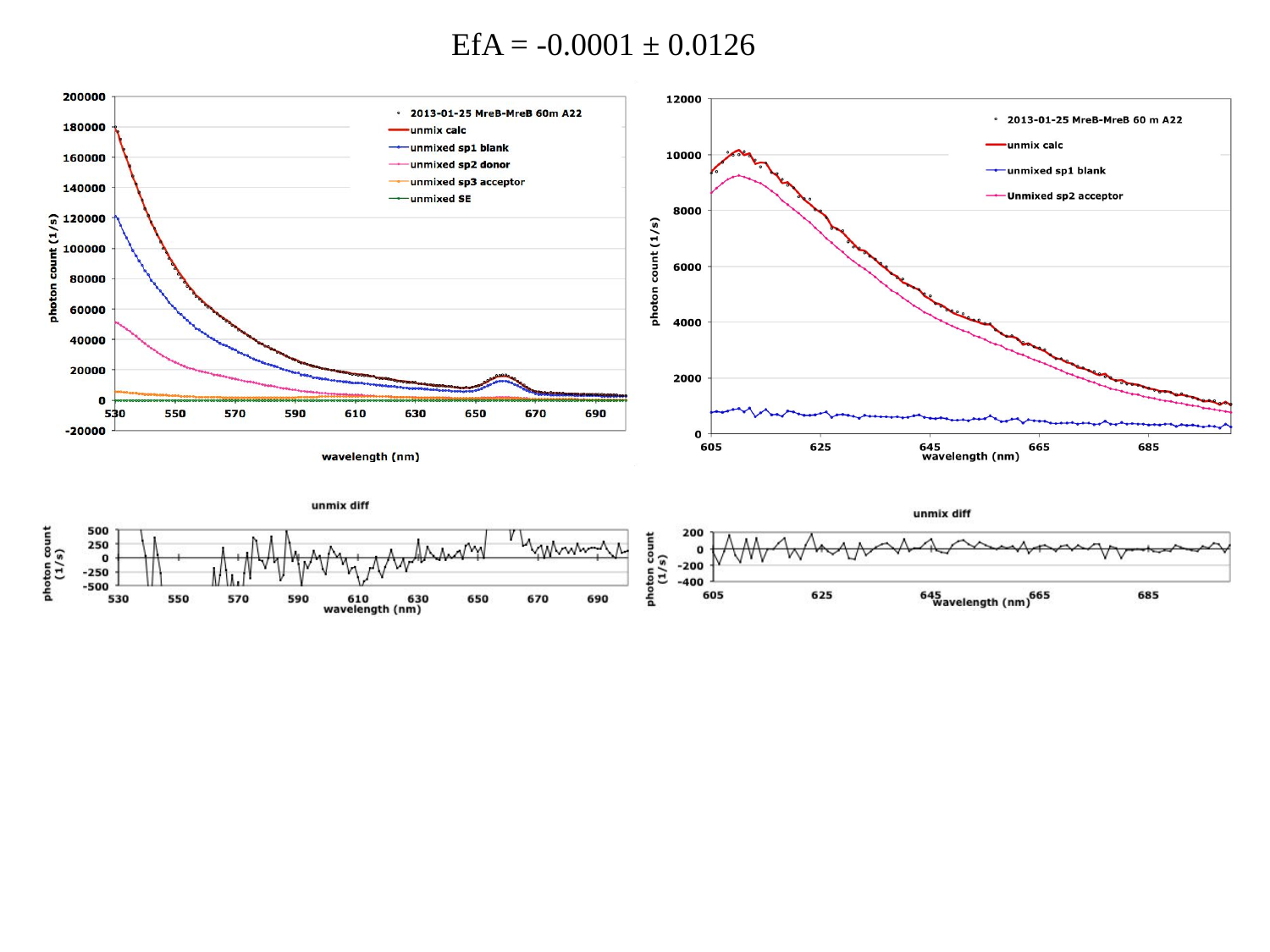

EfA = -0.0001 ± 0.0126

## Slide 29
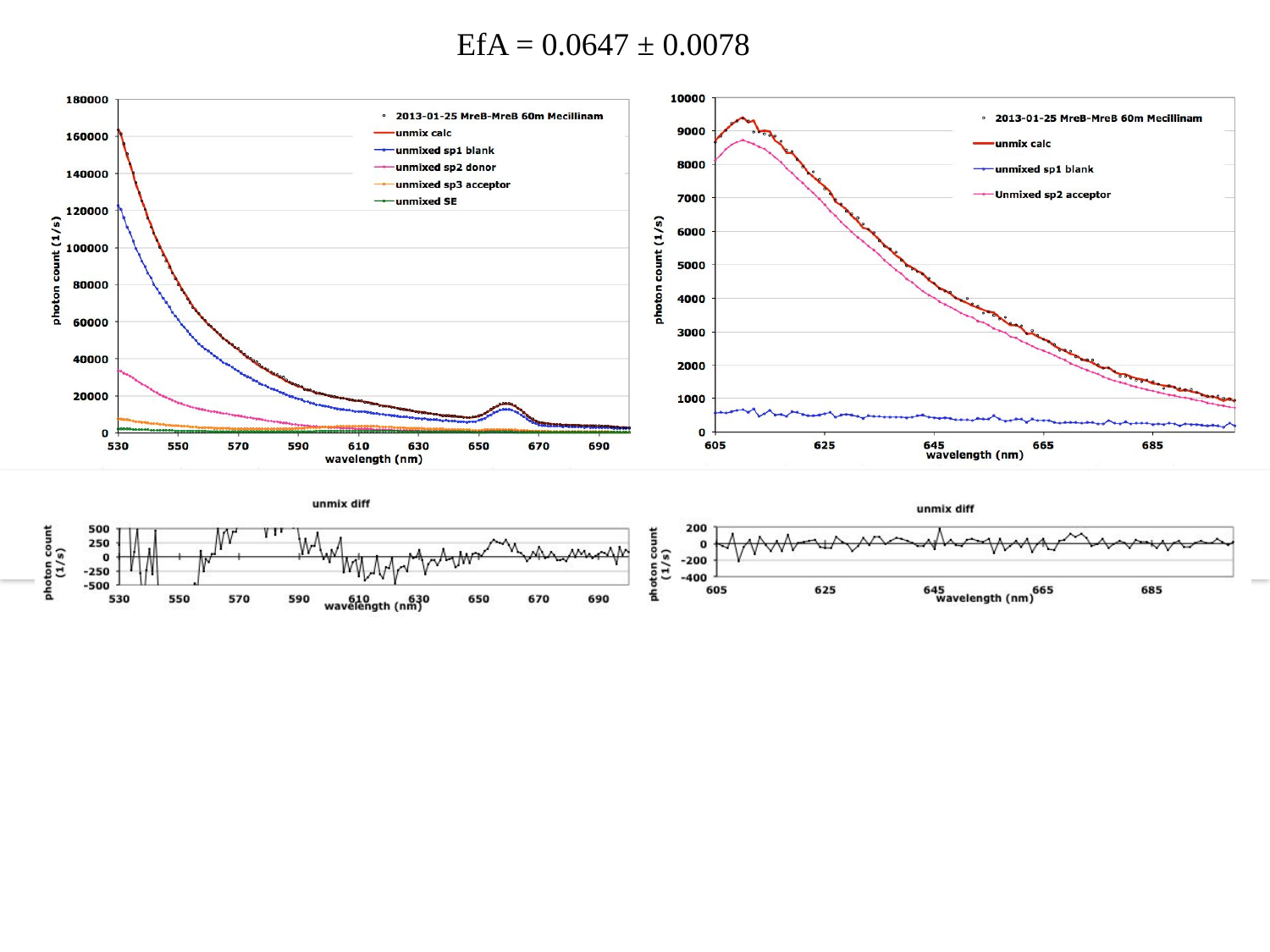

EfA = 0.0647 ± 0.0078

## Slide 30
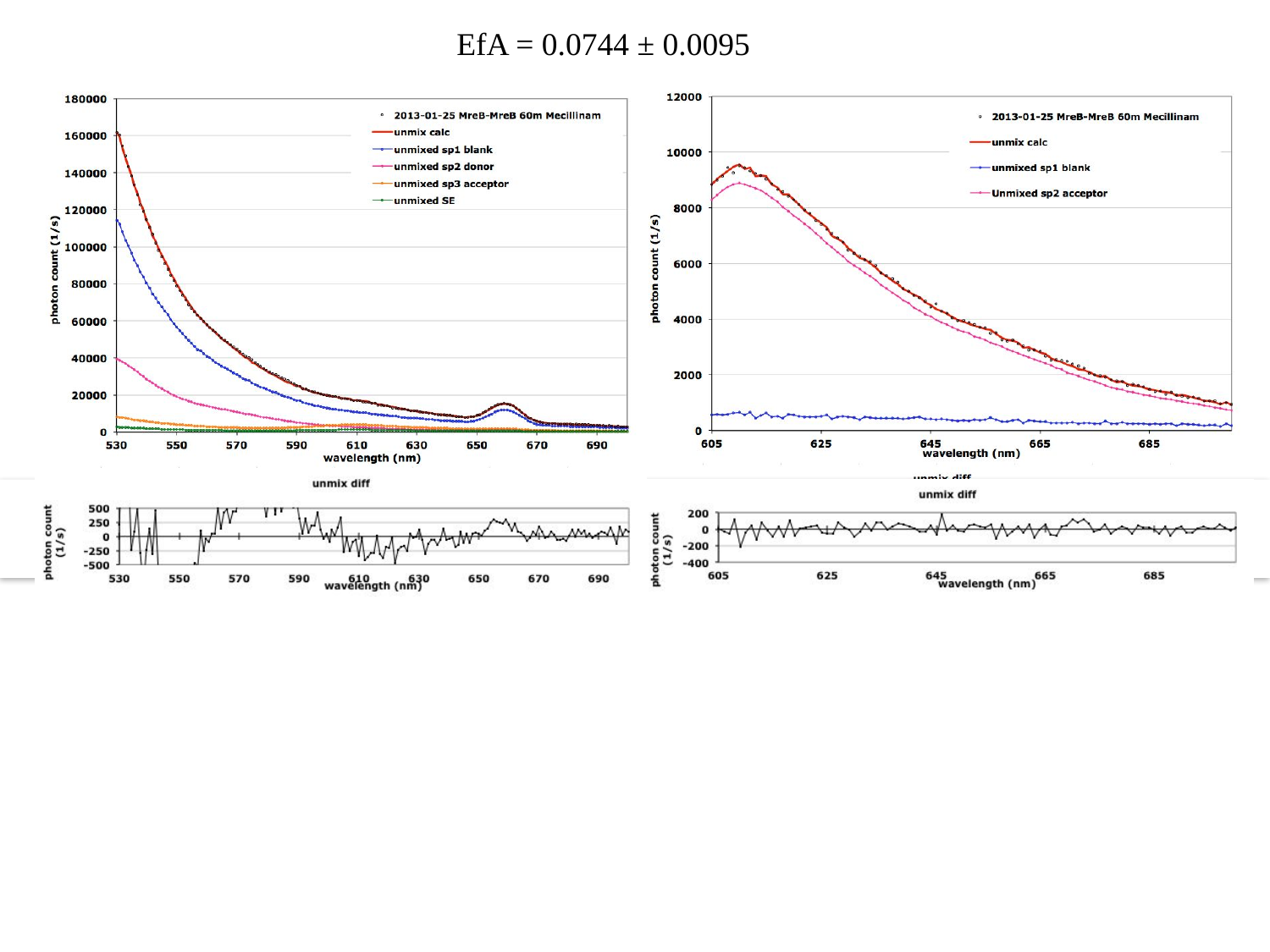

EfA = 0.0744 ± 0.0095

## Slide 31
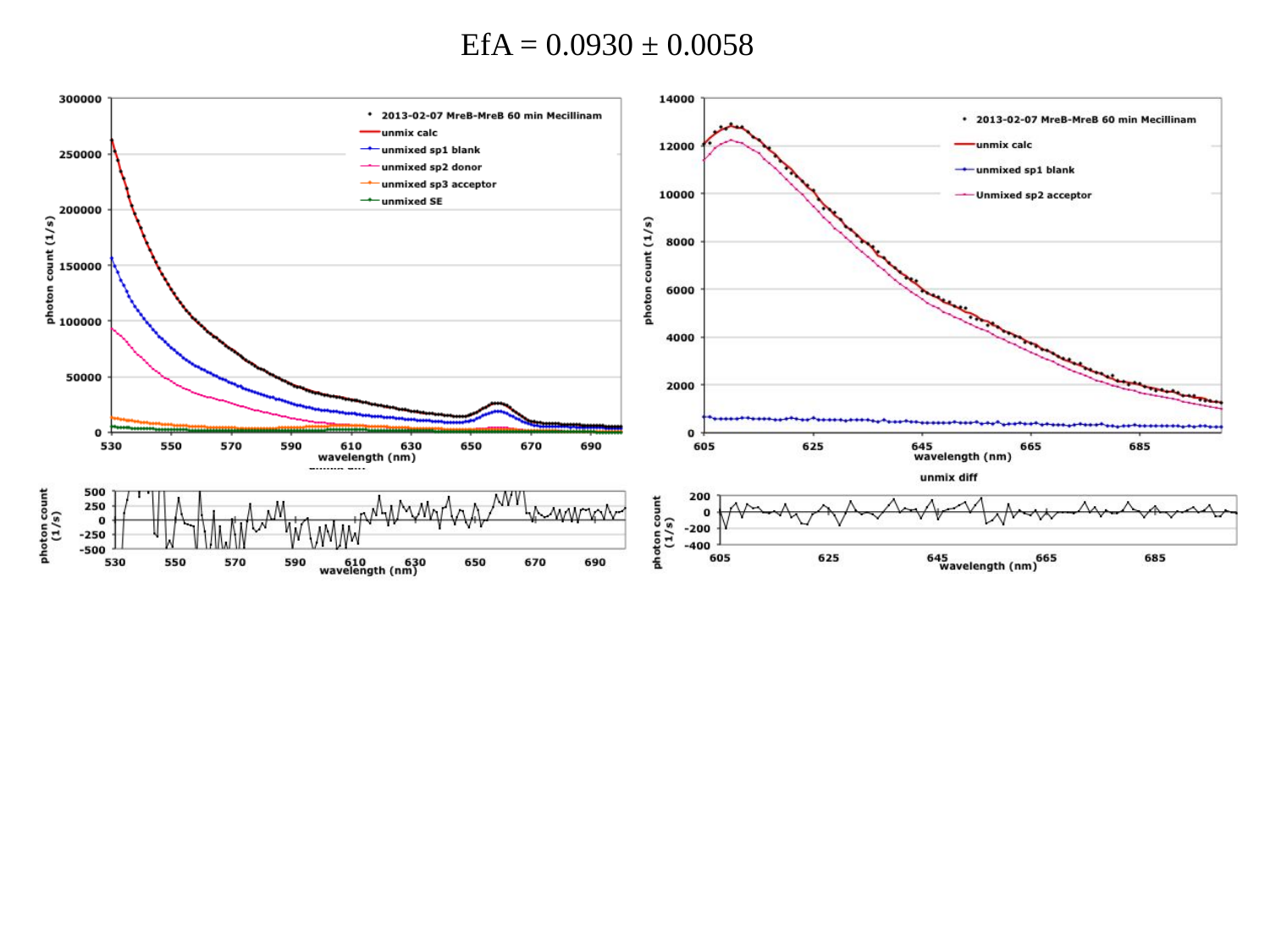

EfA = 0.0930 ± 0.0058

## Slide 32
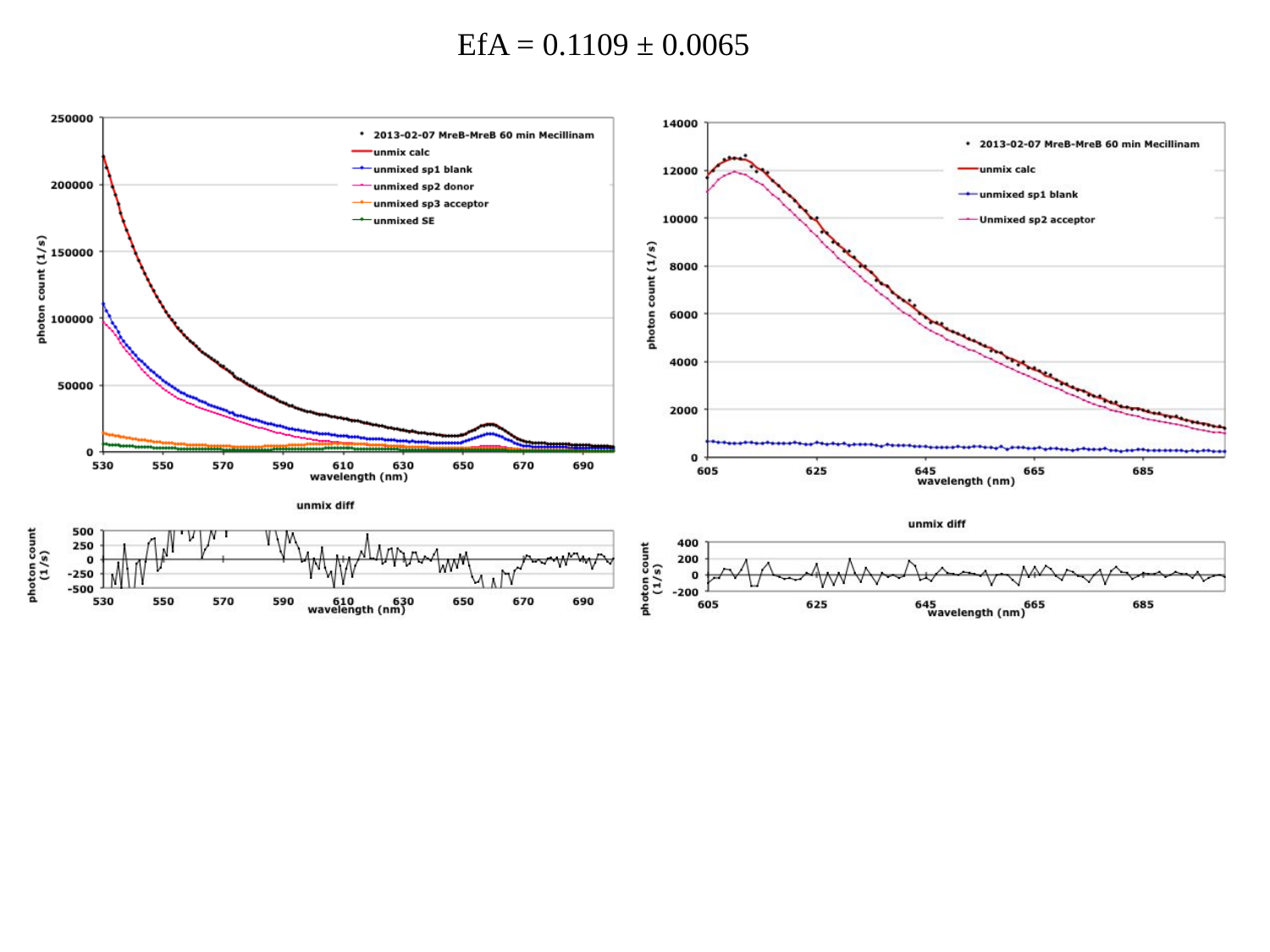

EfA = 0.1109 ± 0.0065

## Slide 33
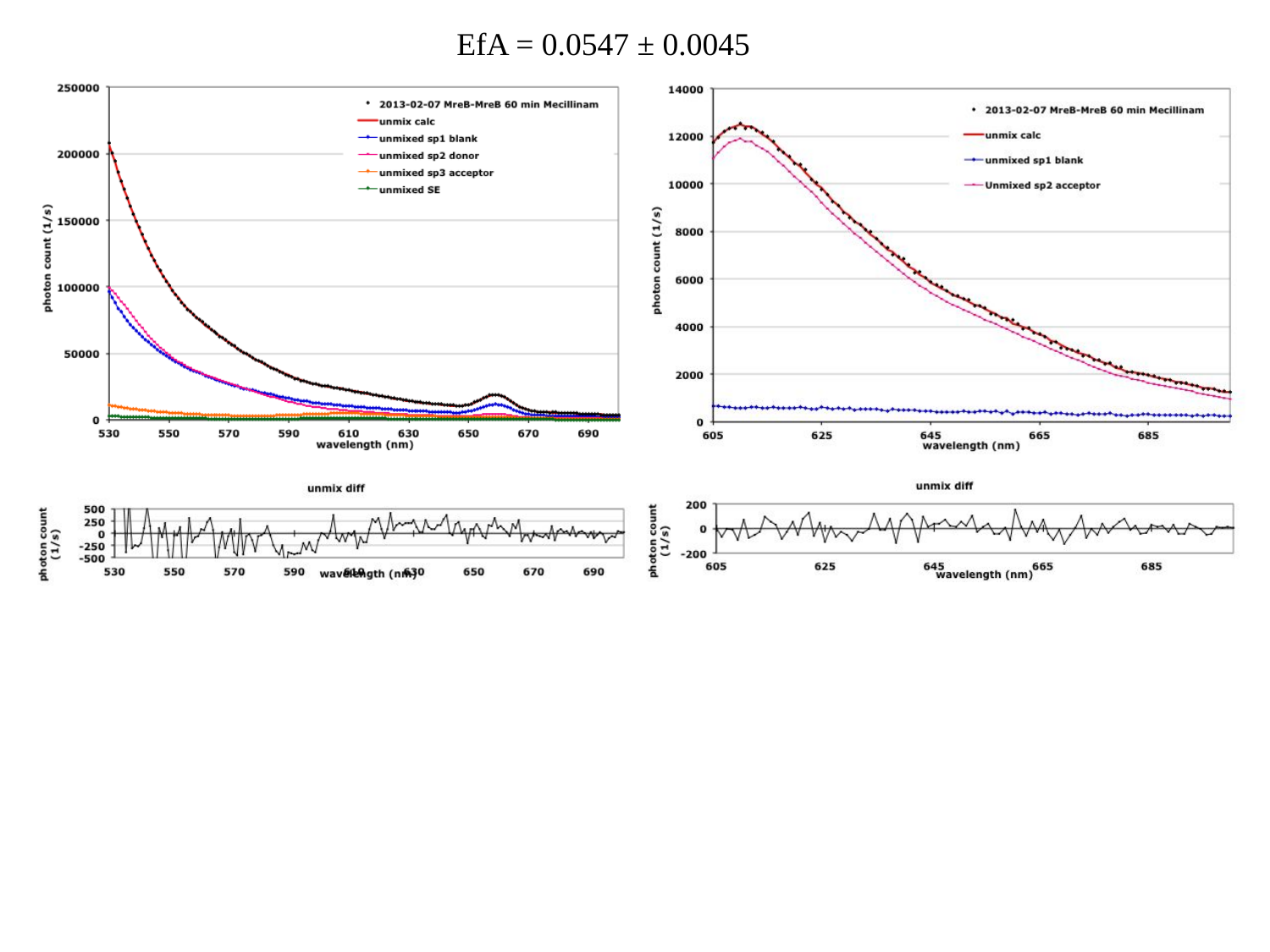

EfA = 0.0547 ± 0.0045

## Slide 34
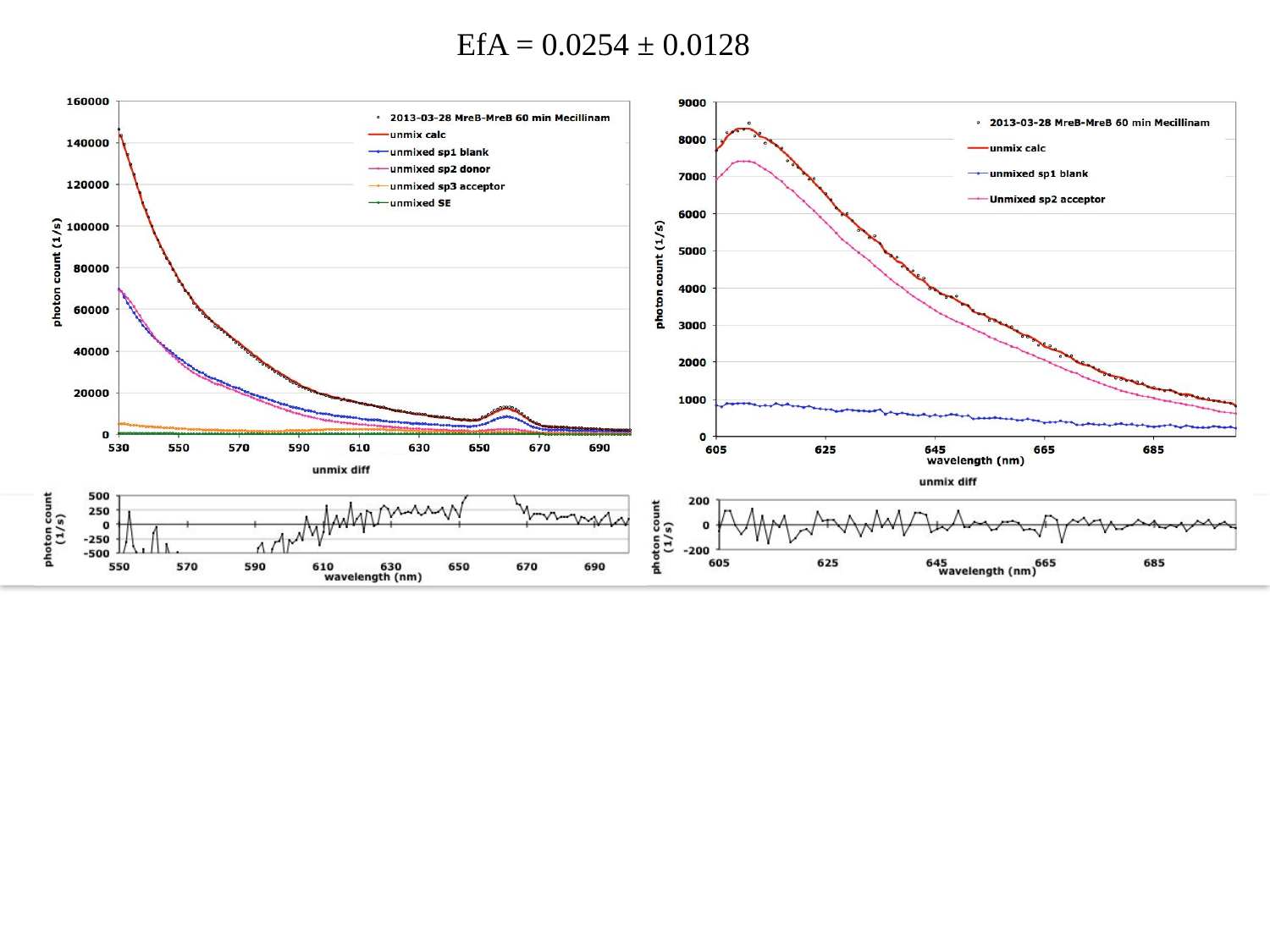

EfA = 0.0254 ± 0.0128

## Slide 35
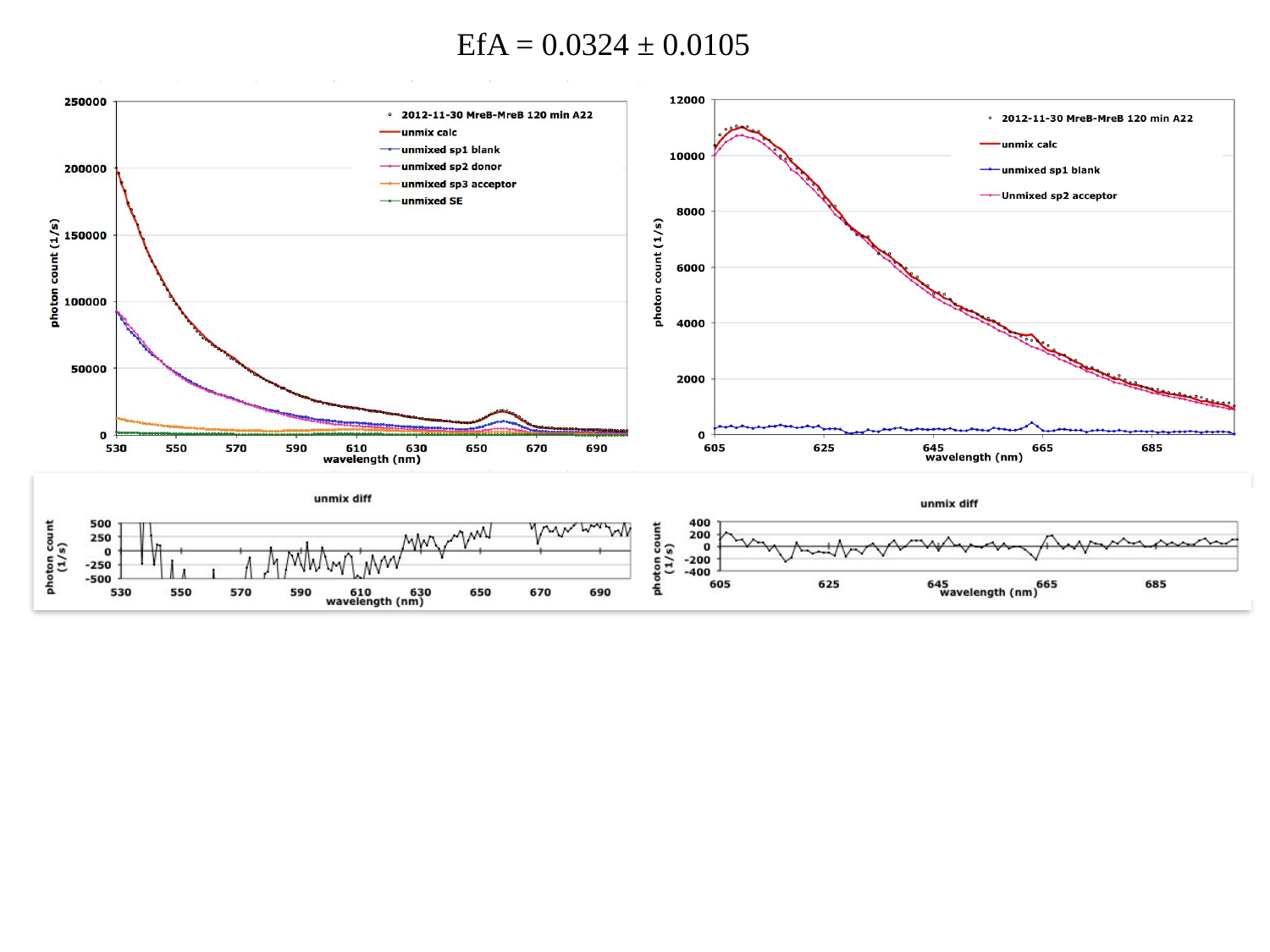

EfA = 0.0324 ± 0.0105

## Slide 36
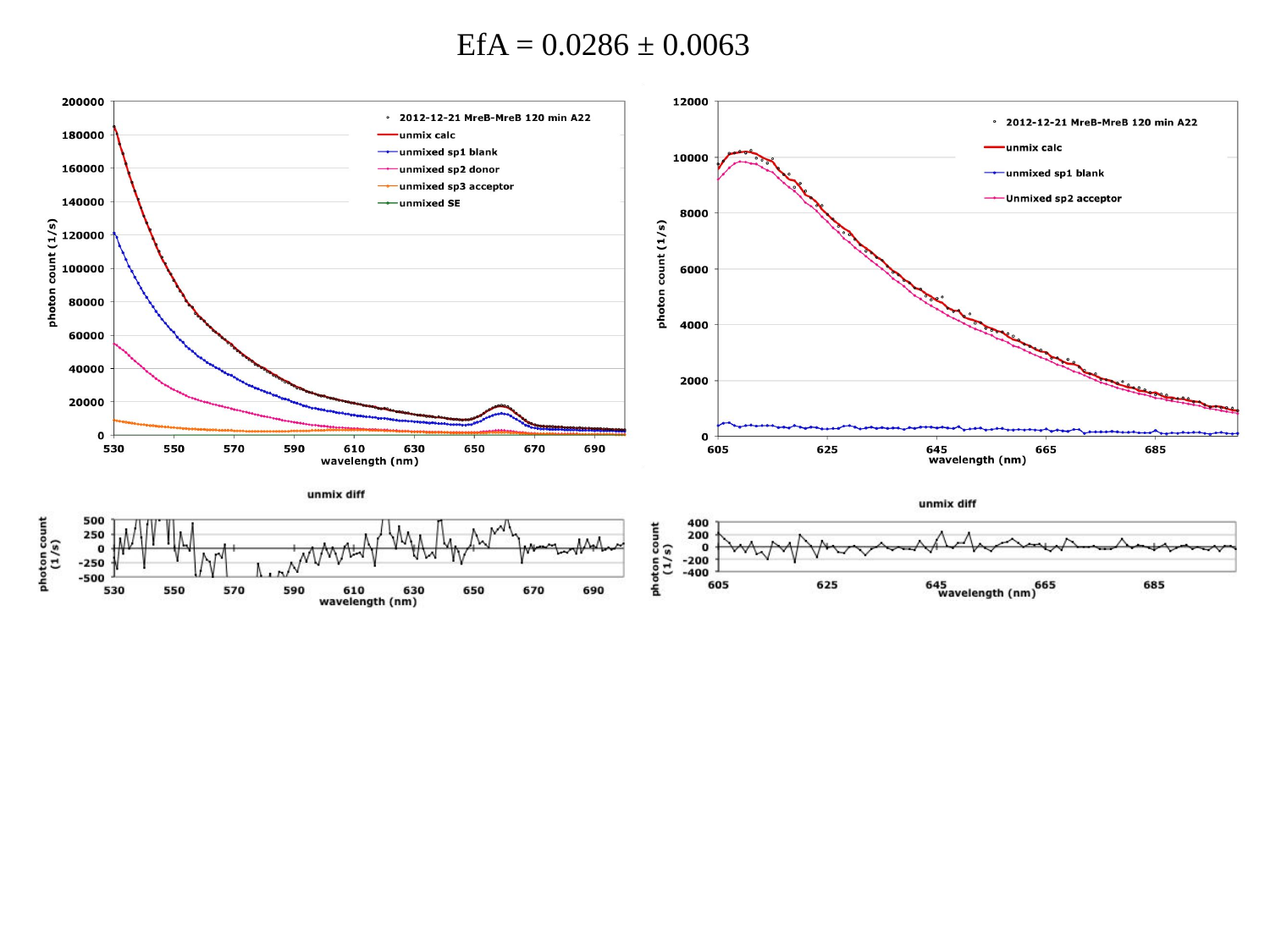

EfA = 0.0286 ± 0.0063

## Slide 37
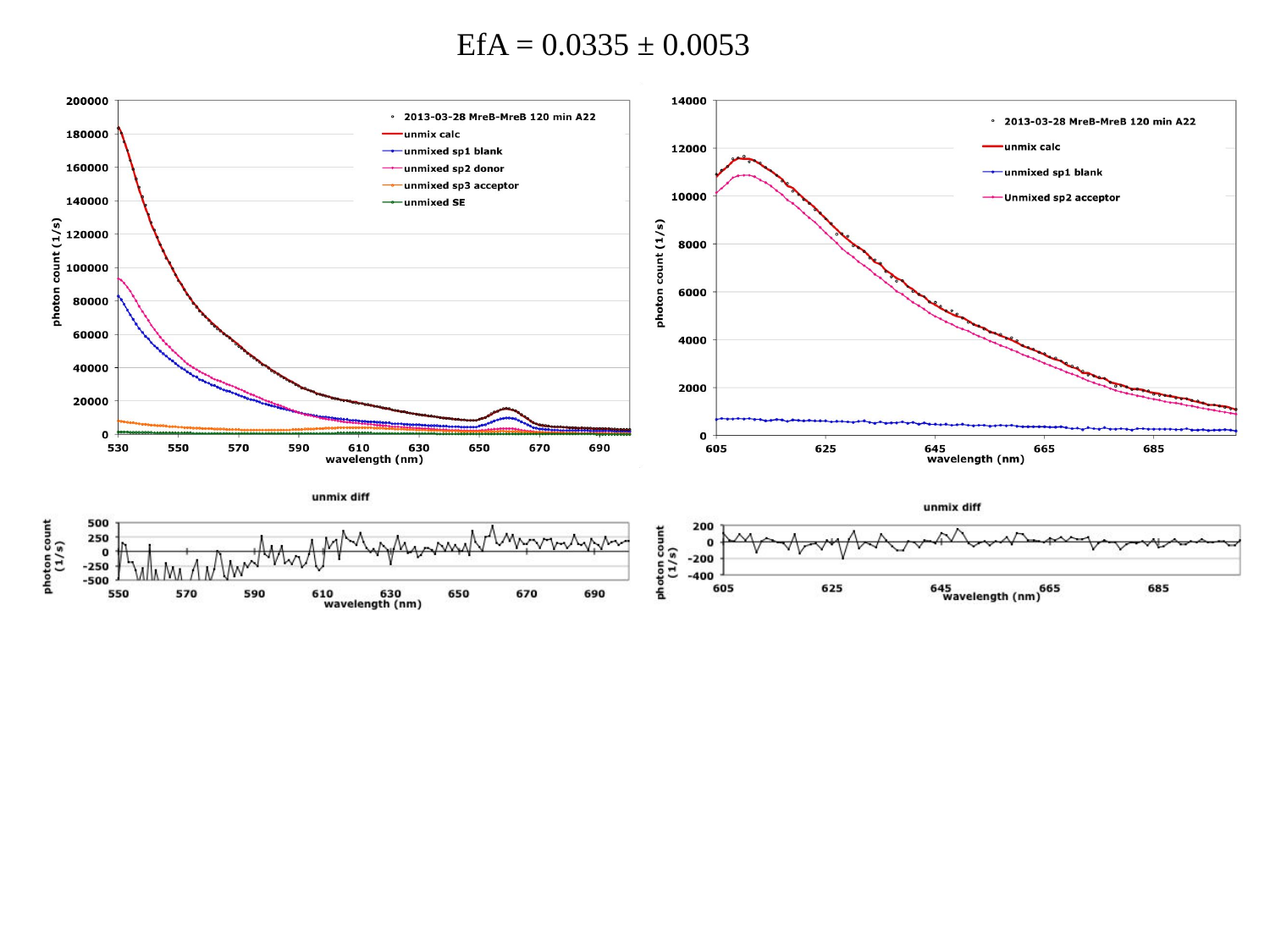

EfA = 0.0335 ± 0.0053

## Slide 38
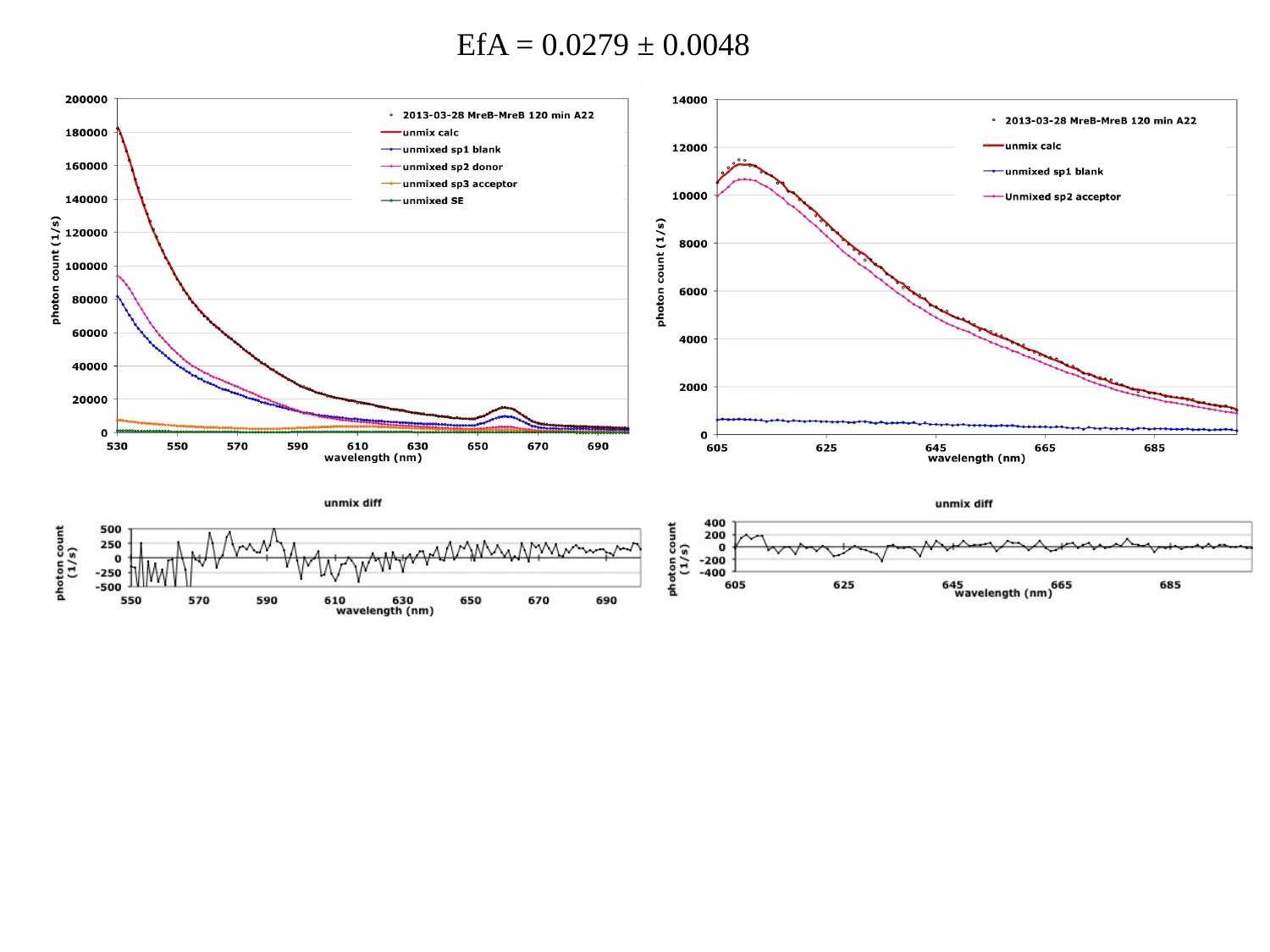

EfA = 0.0279 ± 0.0048

## Slide 39
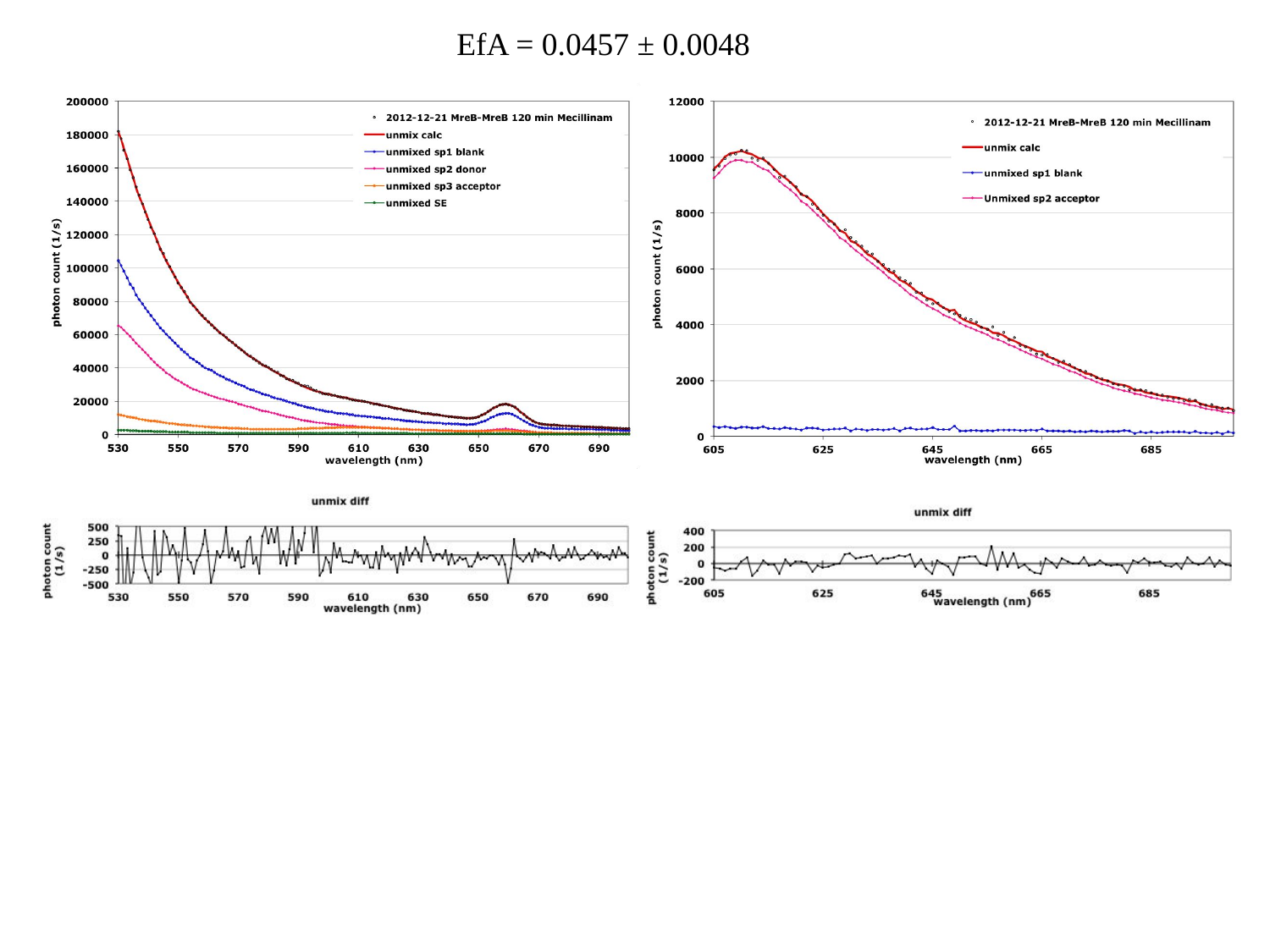

EfA = 0.0457 ± 0.0048

## Slide 40
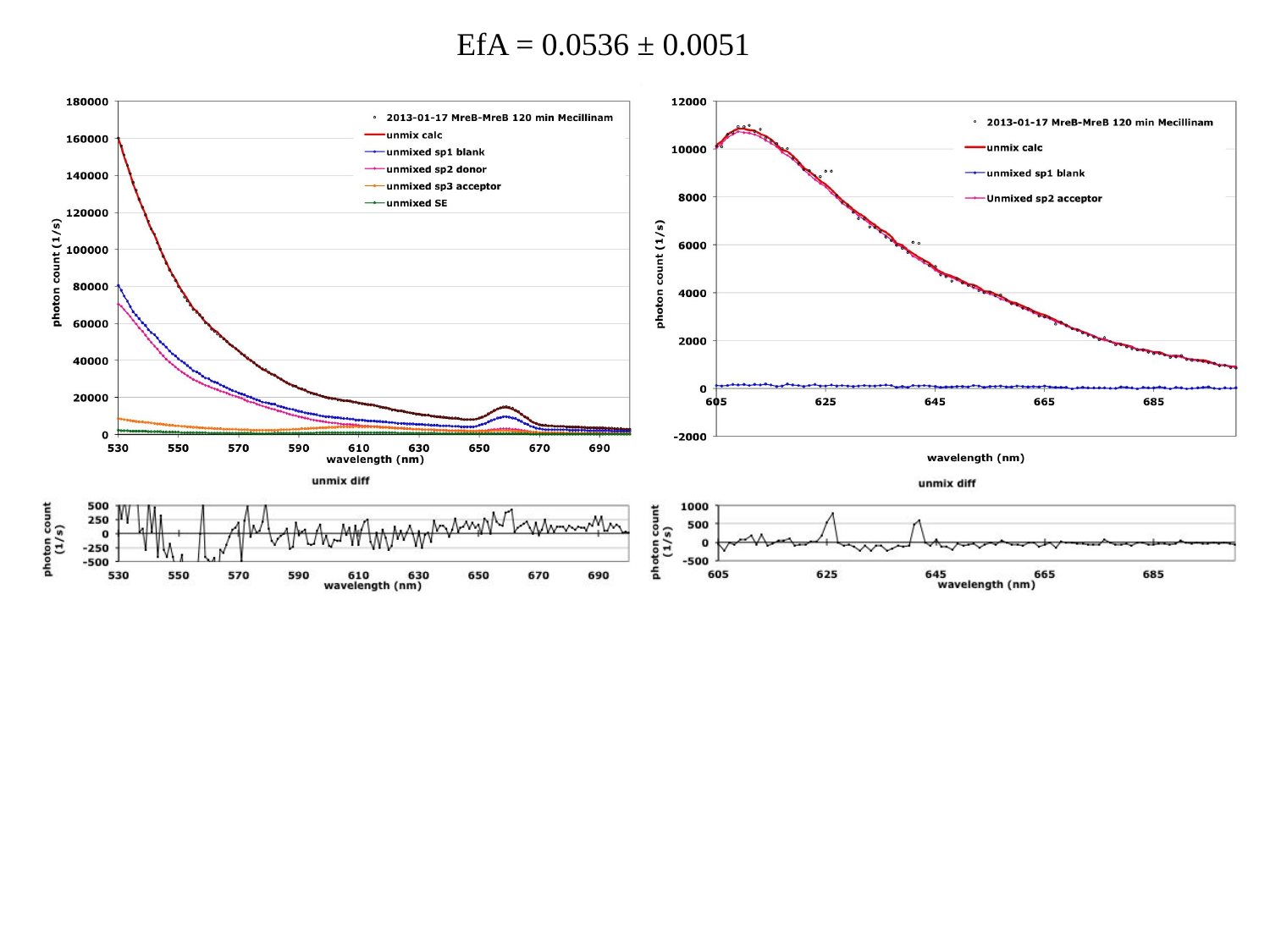

EfA = 0.0536 ± 0.0051

## Slide 41
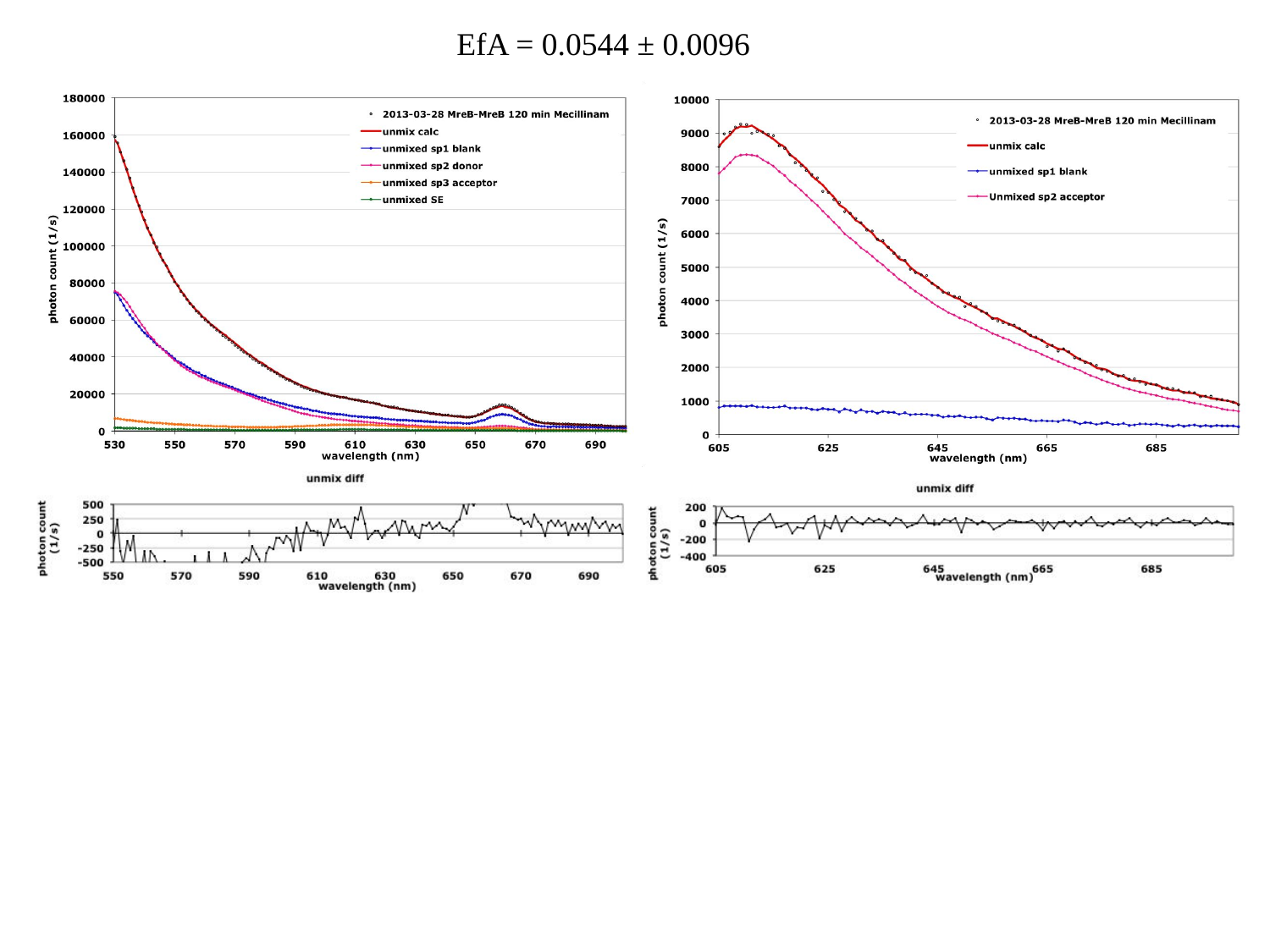

EfA = 0.0544 ± 0.0096

## Slide 42
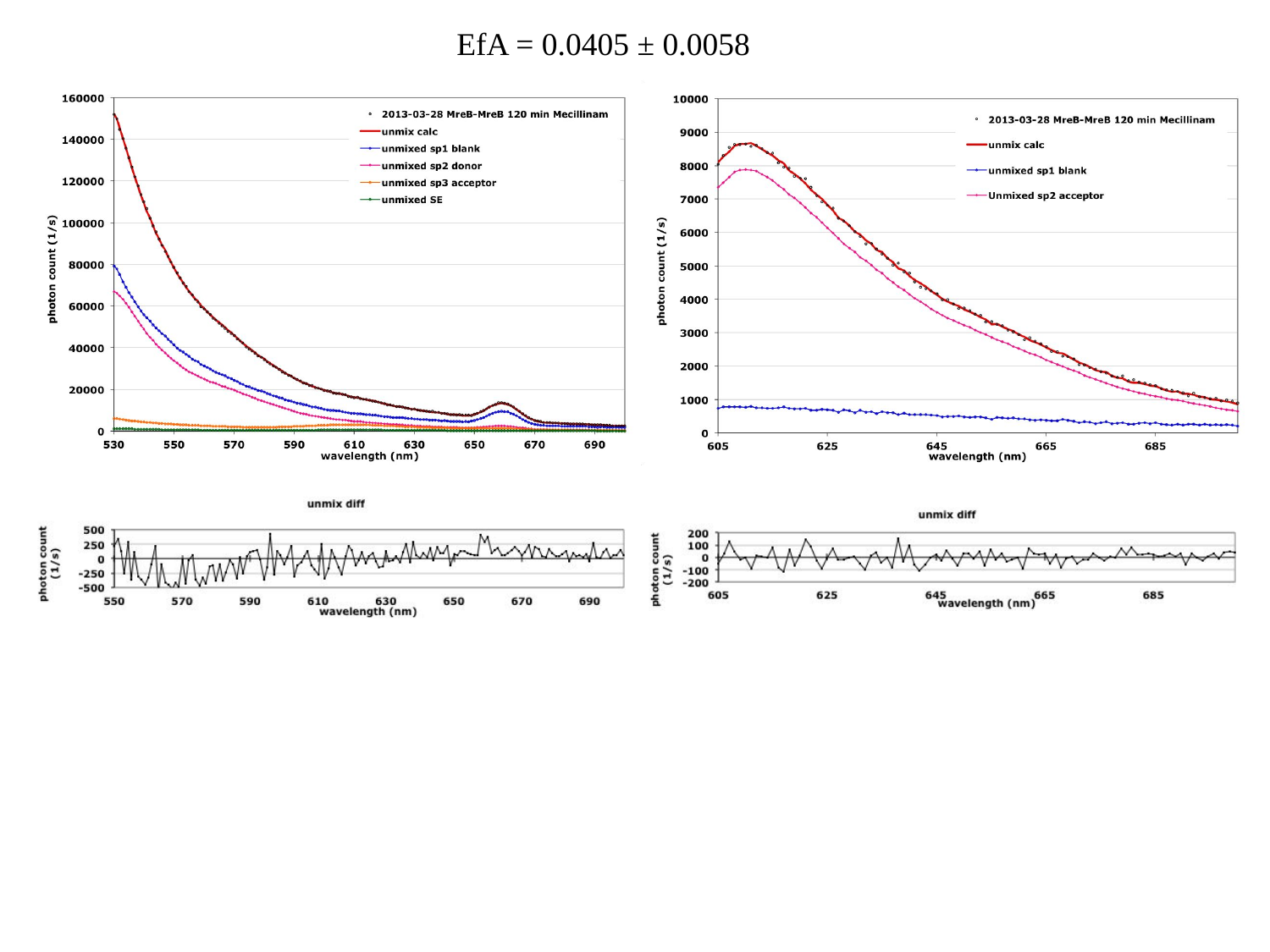

EfA = 0.0405 ± 0.0058

## Slide 43
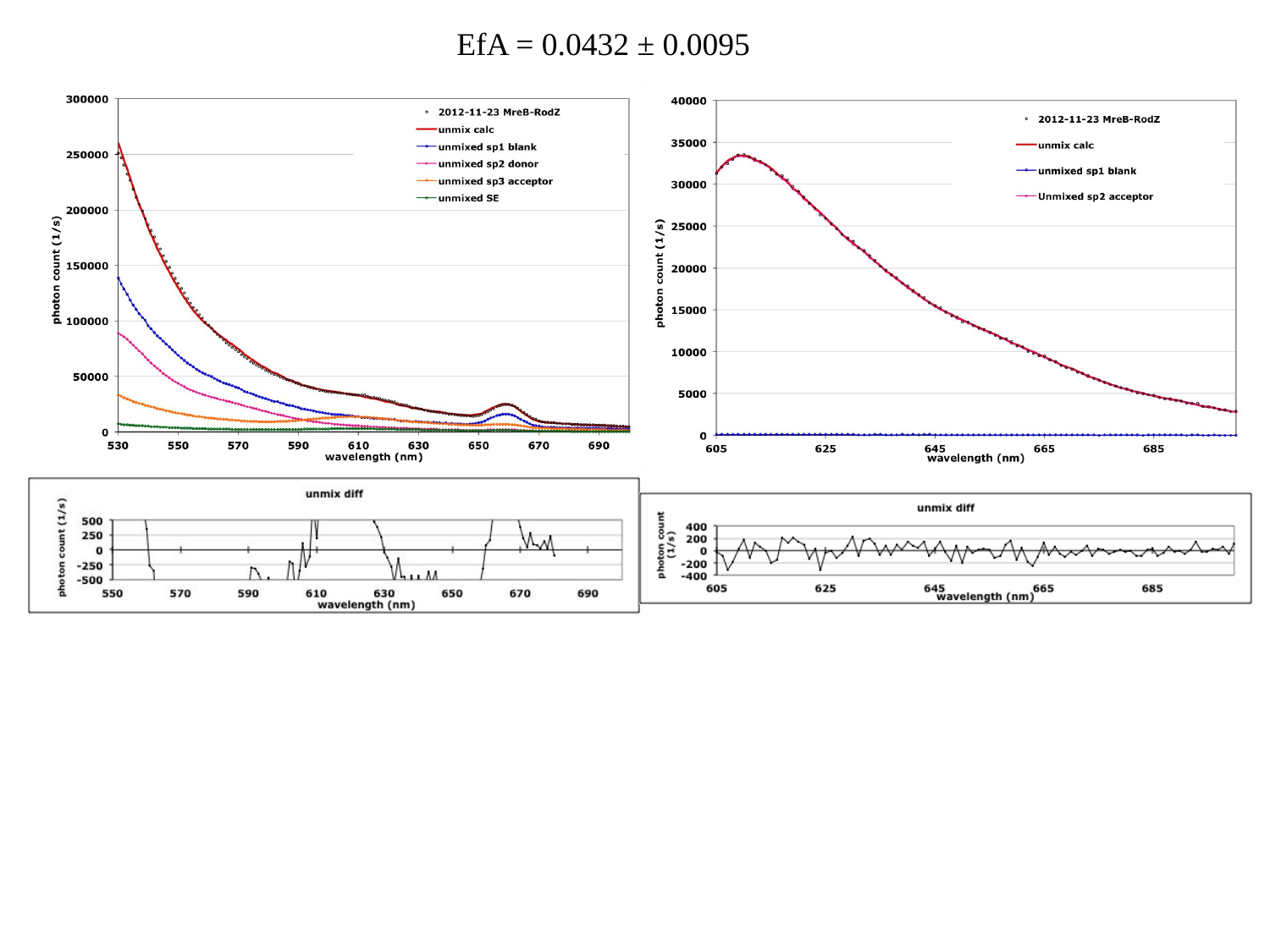

EfA = 0.0432 ± 0.0095

## Slide 44
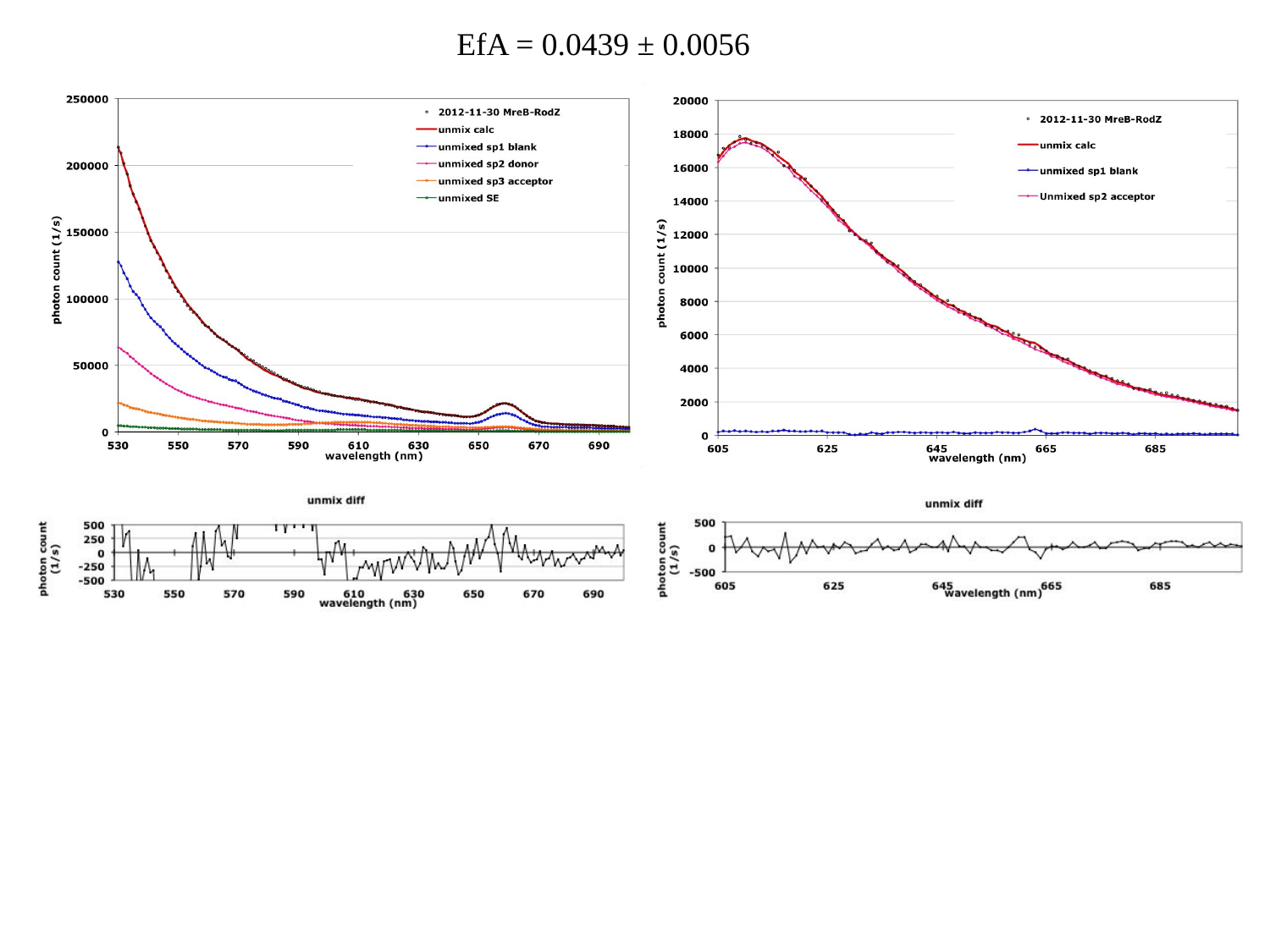

EfA = 0.0439 ± 0.0056

## Slide 45
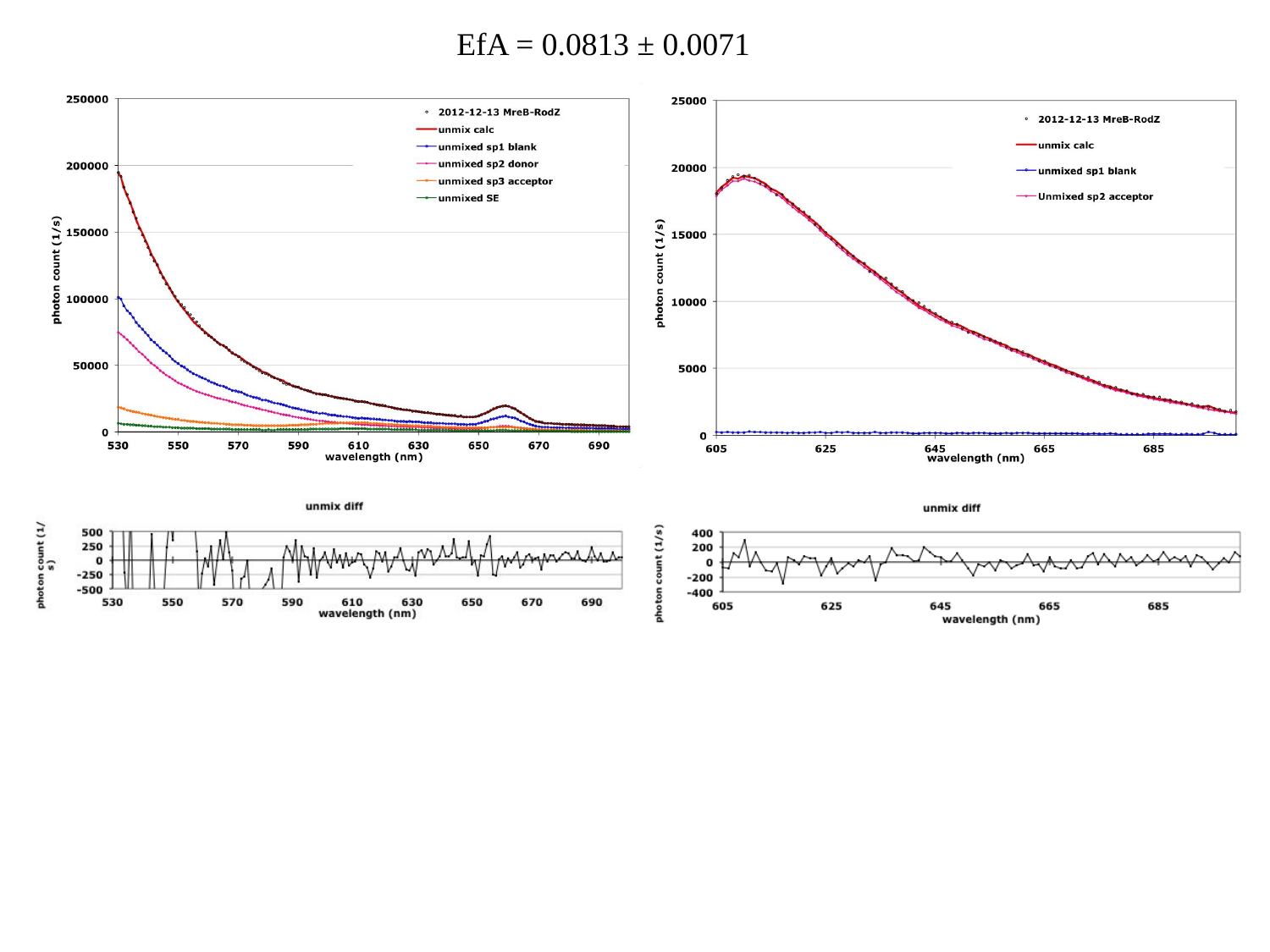

EfA = 0.0813 ± 0.0071

## Slide 46
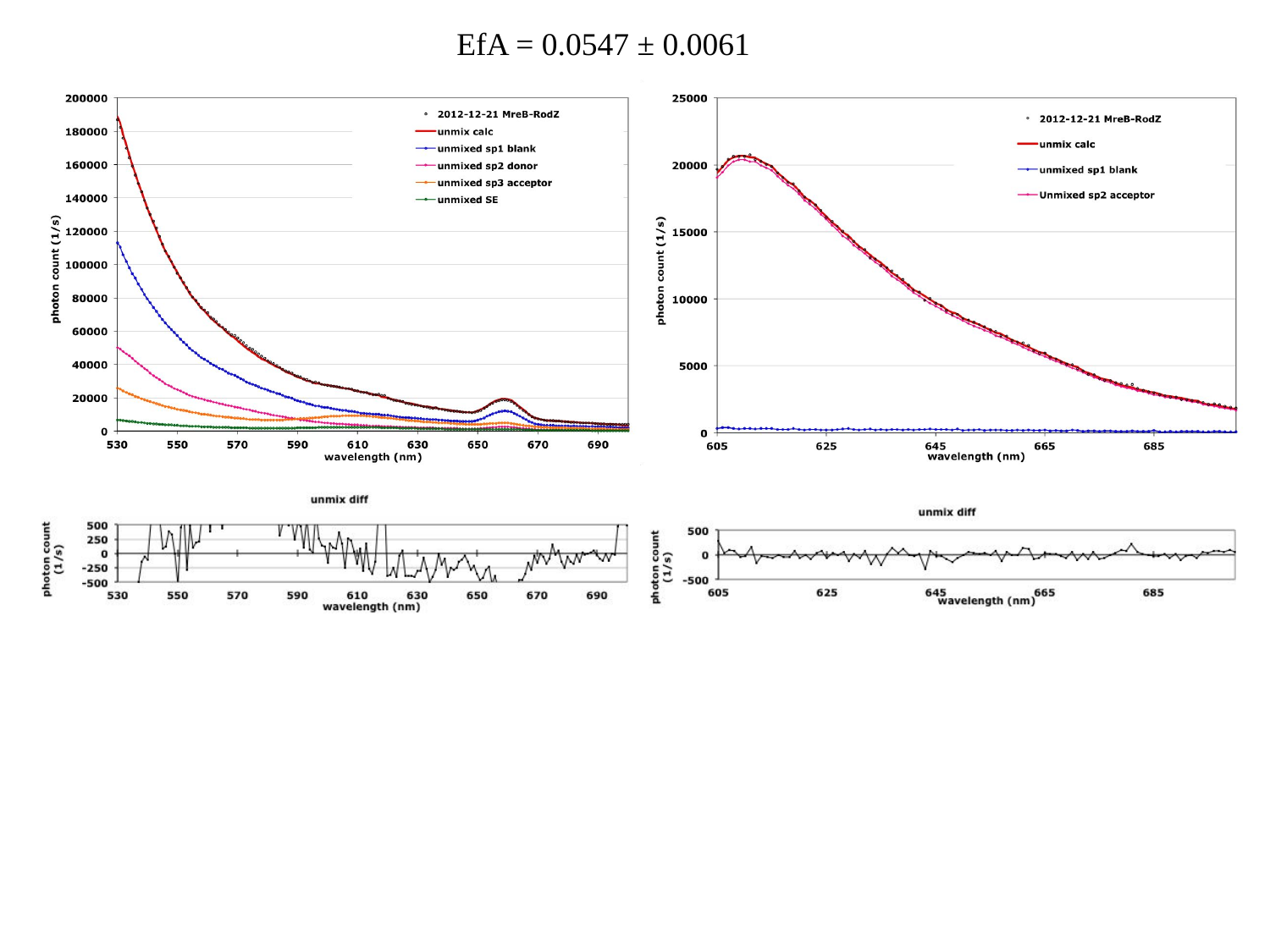

EfA = 0.0547 ± 0.0061

## Slide 47
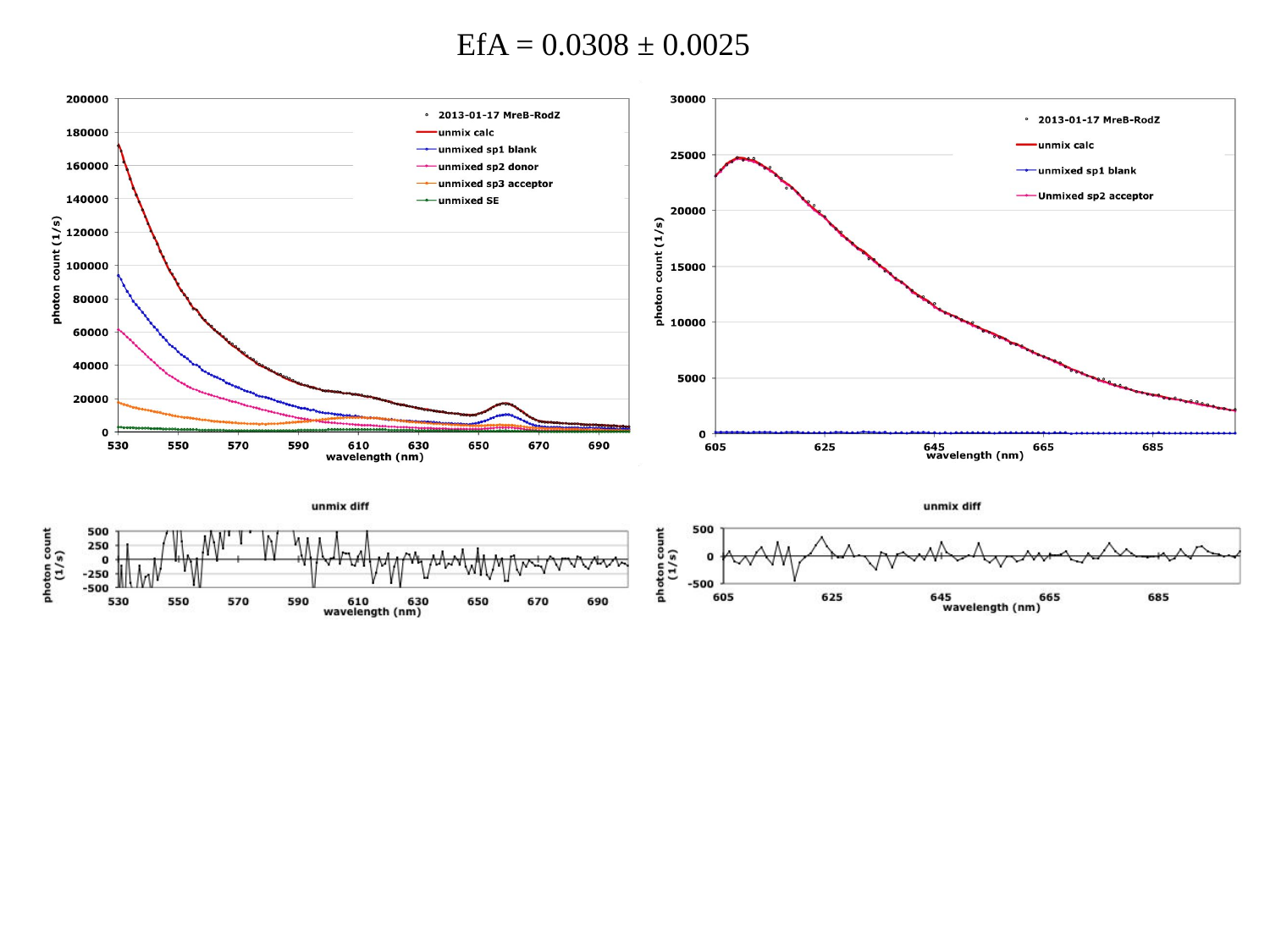

EfA = 0.0308 ± 0.0025

## Slide 48
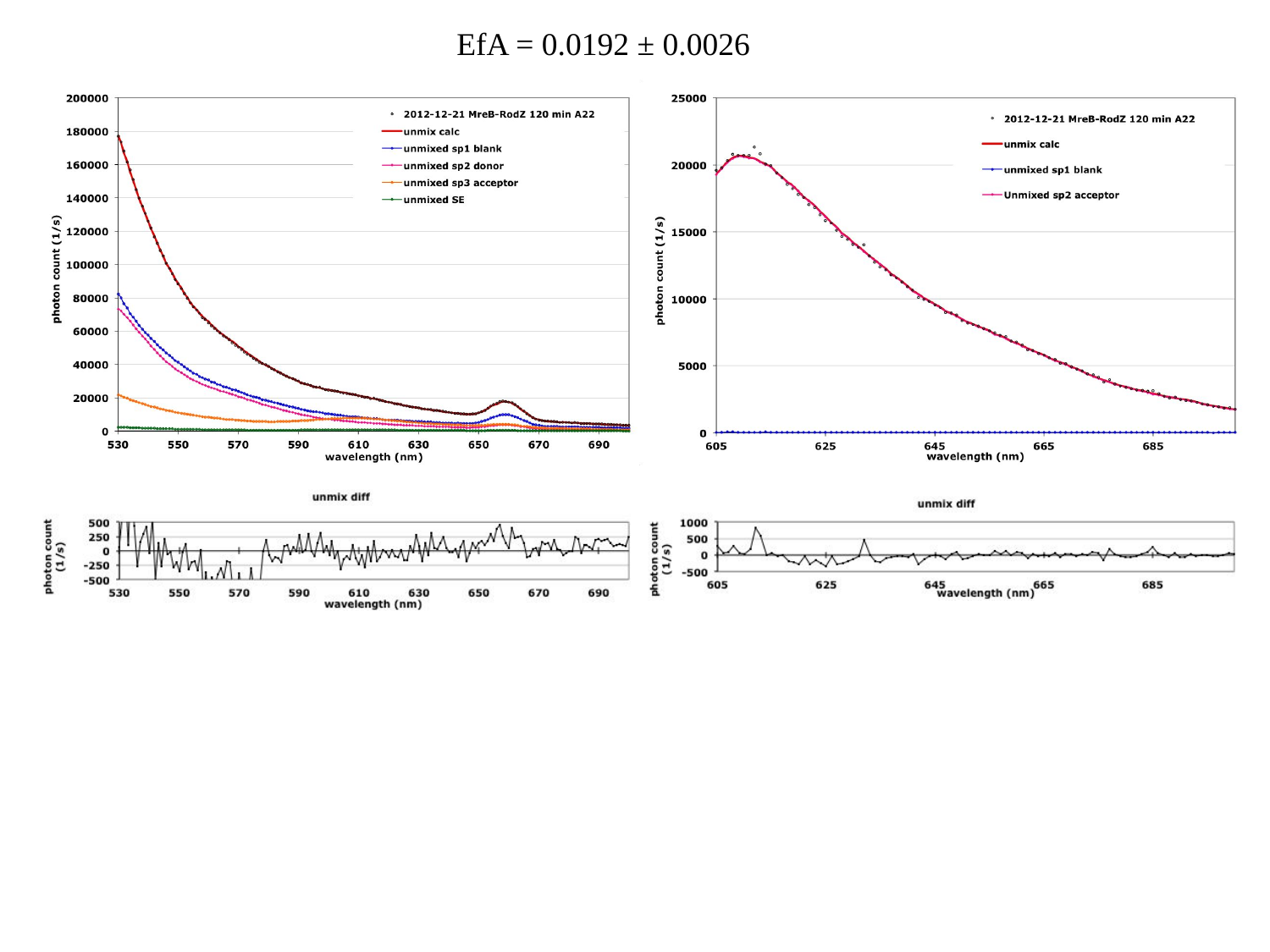

EfA = 0.0192 ± 0.0026

## Slide 49
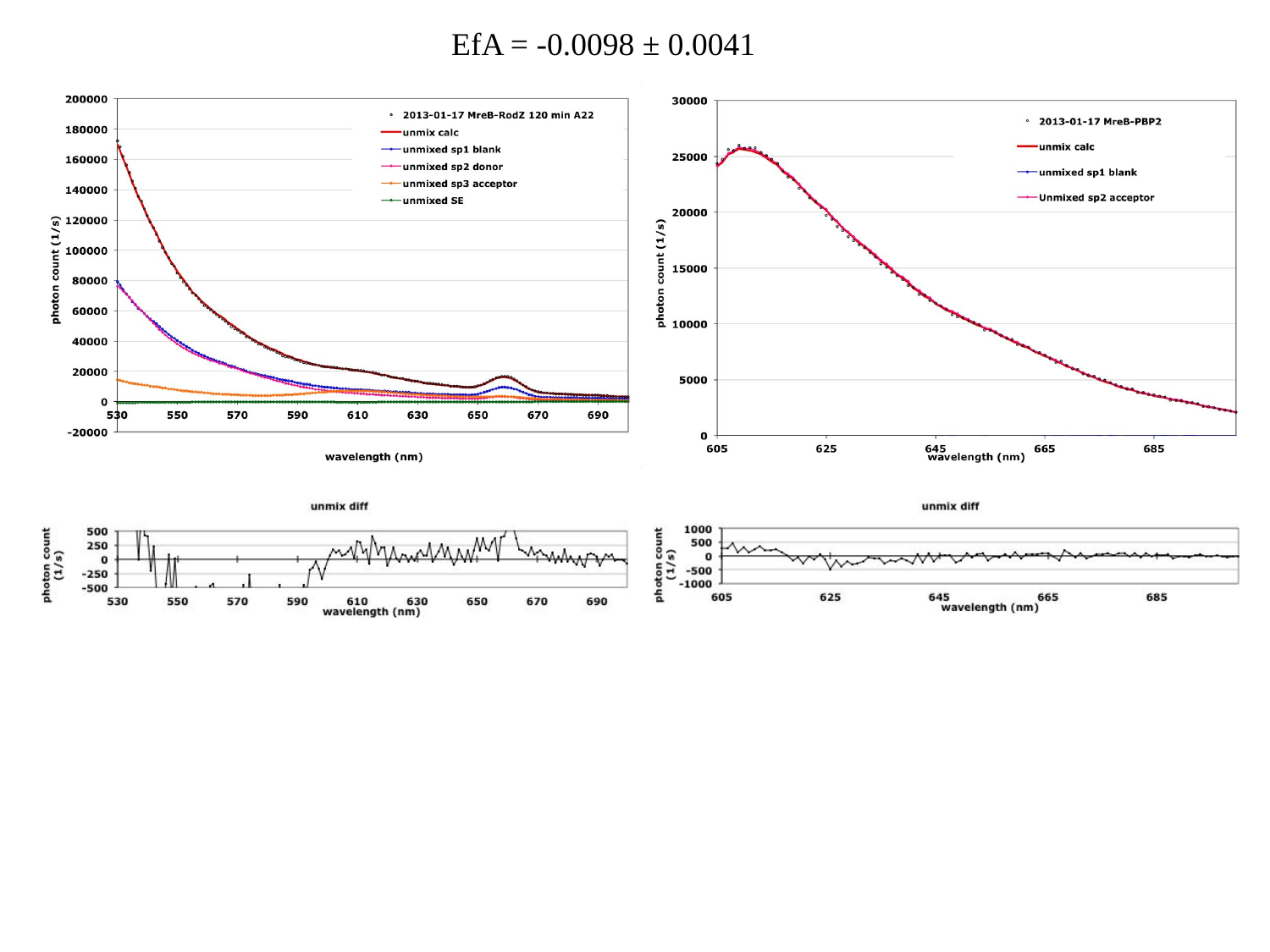

EfA = -0.0098 ± 0.0041

## Slide 50
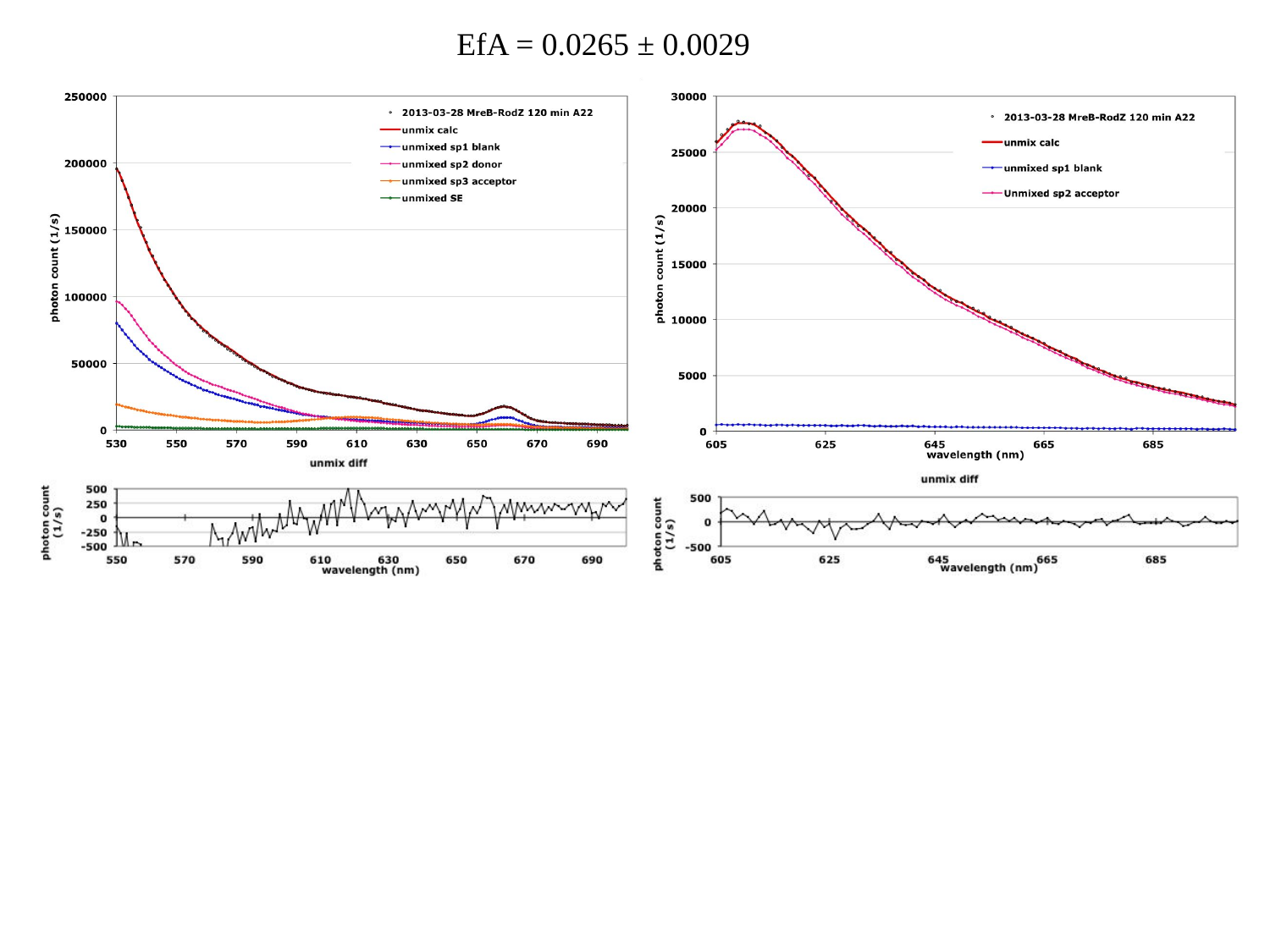

EfA = 0.0265 ± 0.0029

## Slide 51
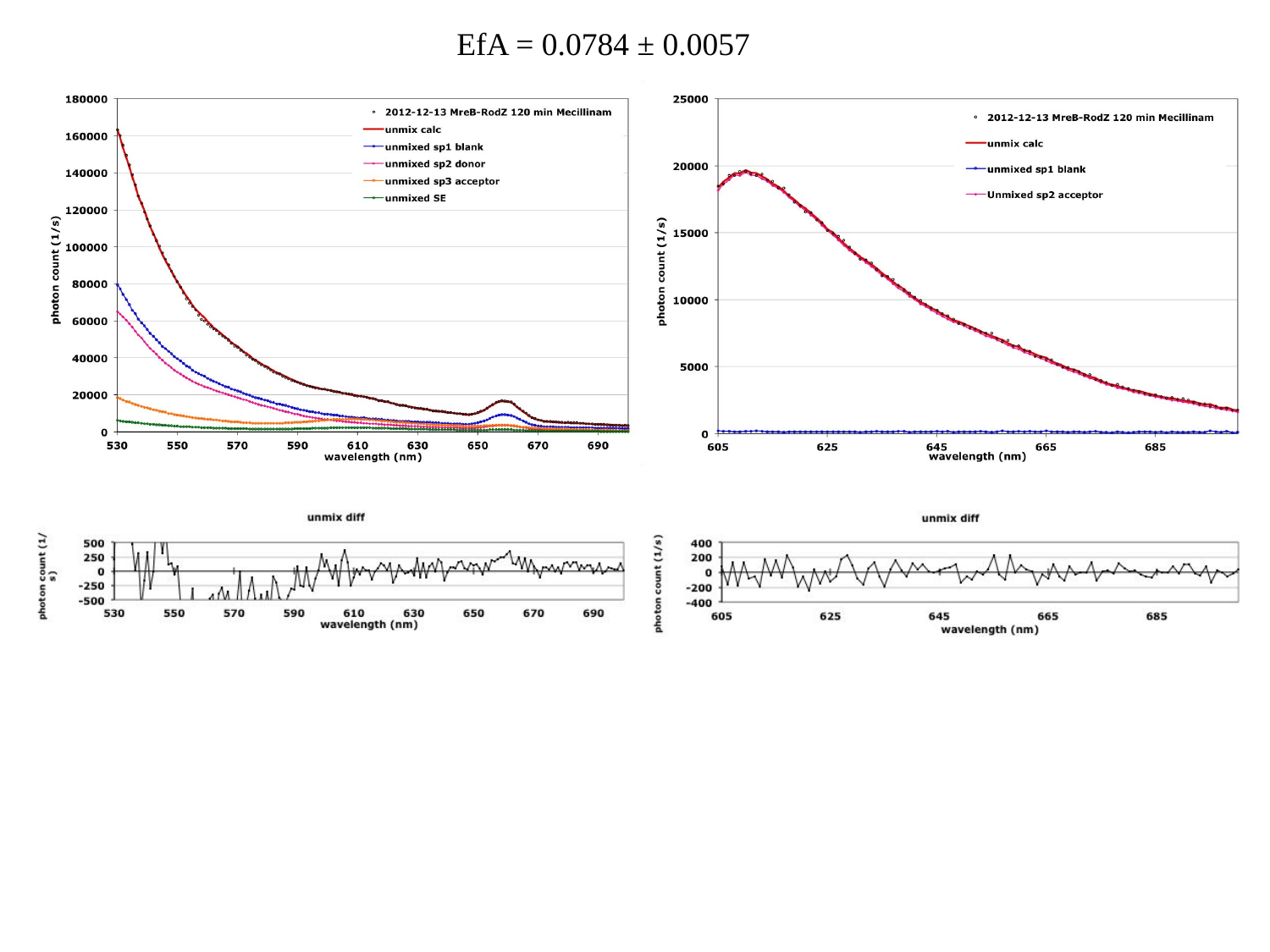

EfA = 0.0784 ± 0.0057

## Slide 52
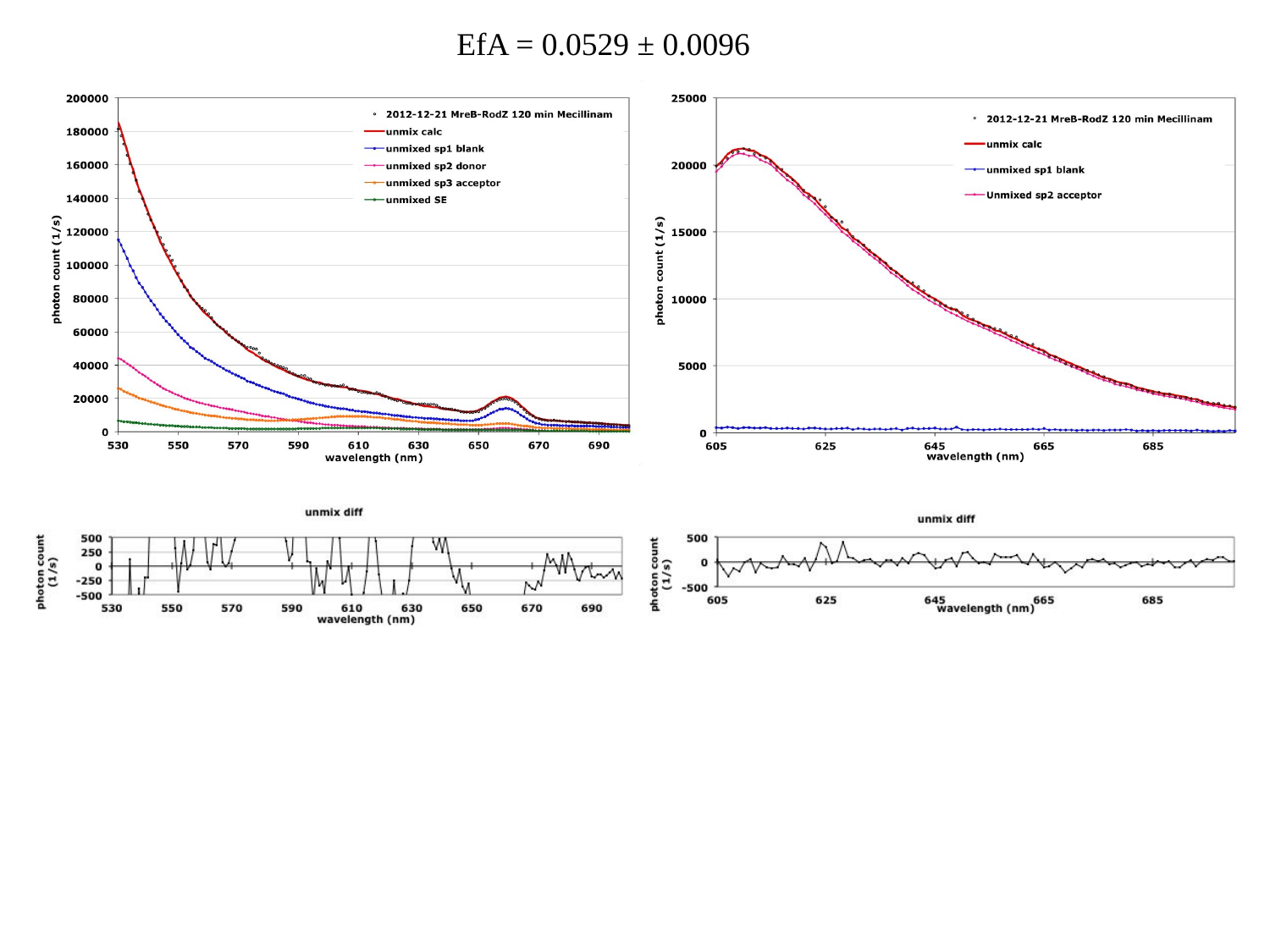

EfA = 0.0529 ± 0.0096

## Slide 53
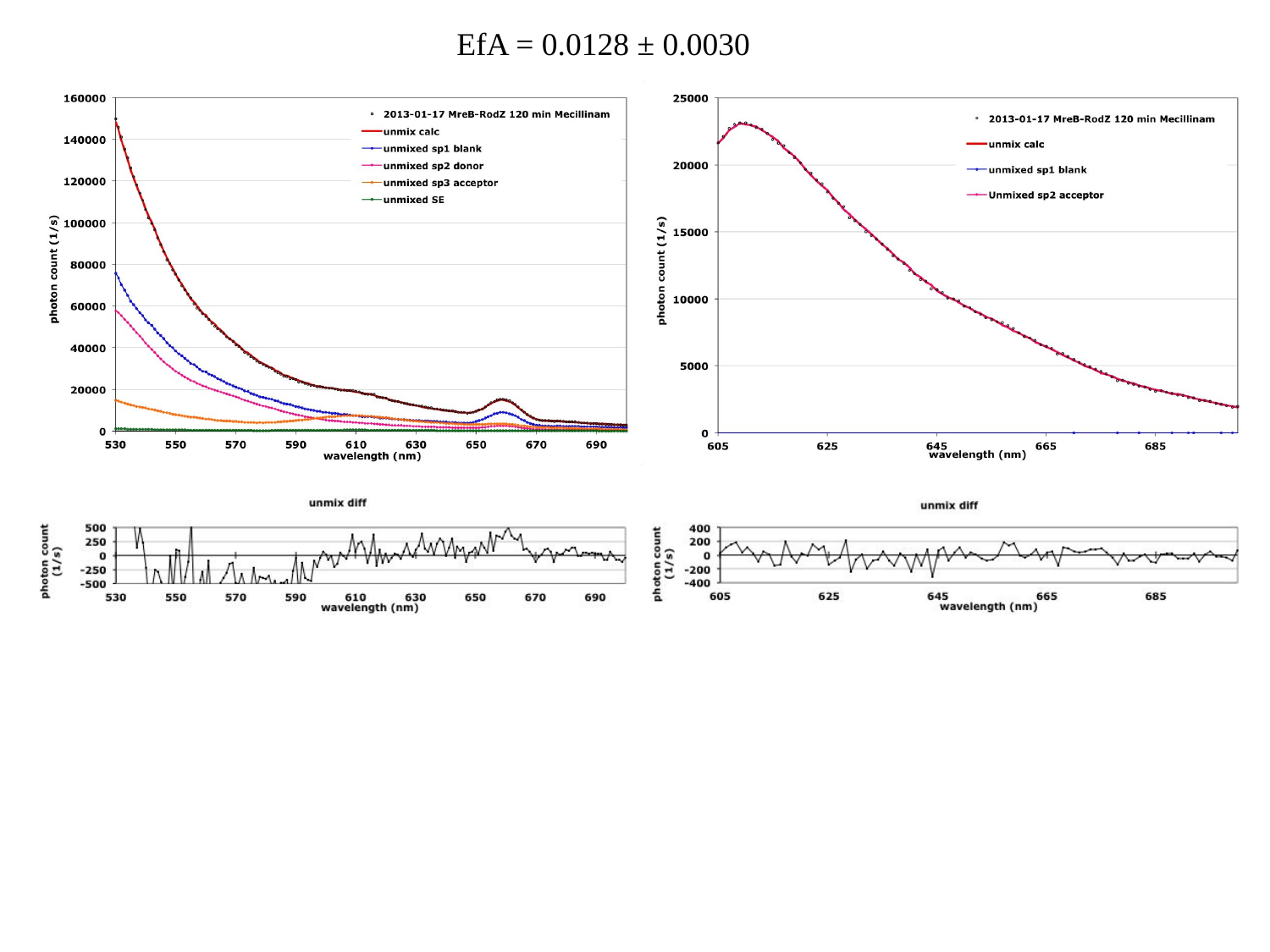

EfA = 0.0128 ± 0.0030

## Slide 54
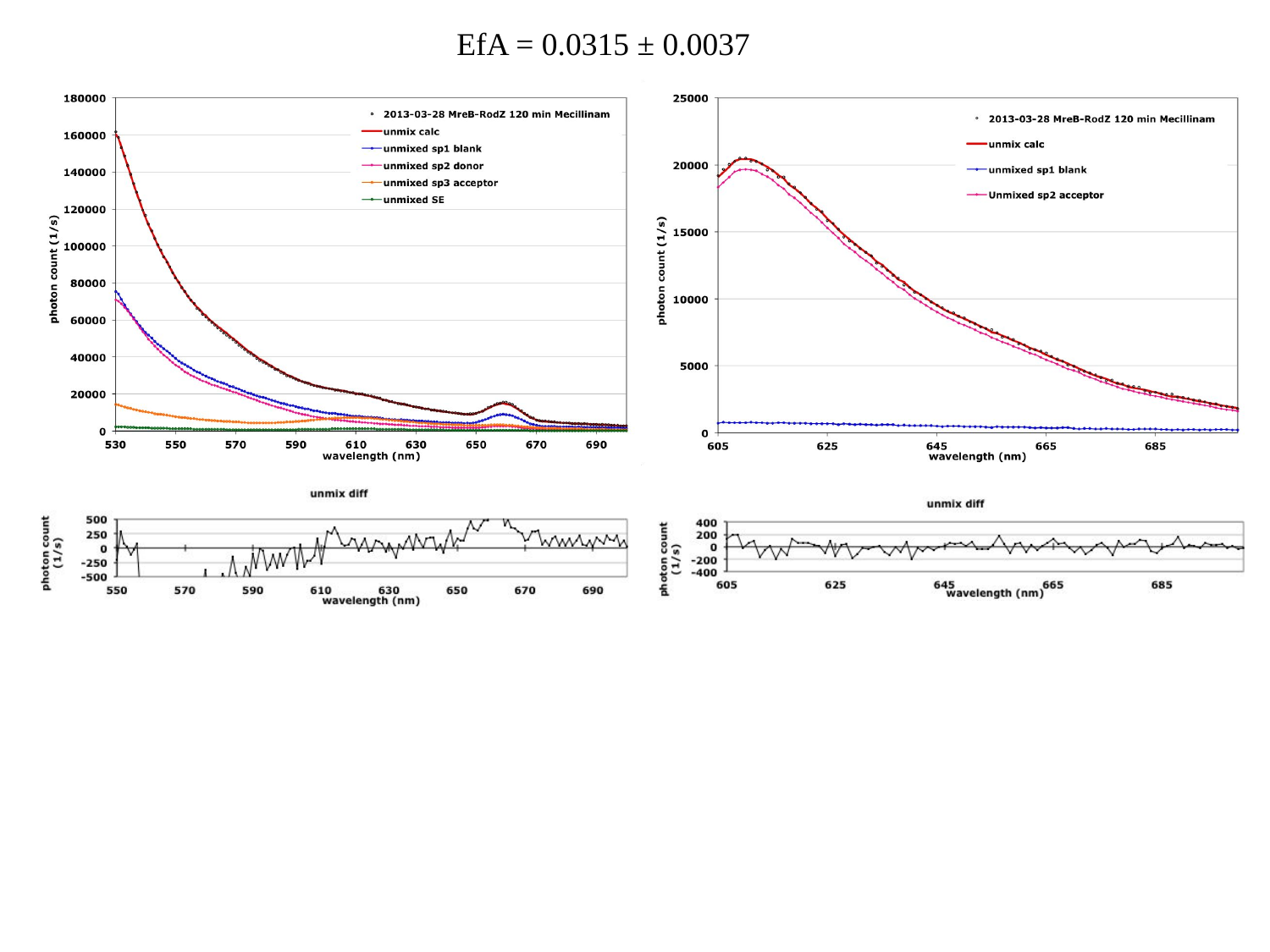

EfA = 0.0315 ± 0.0037

## Slide 55
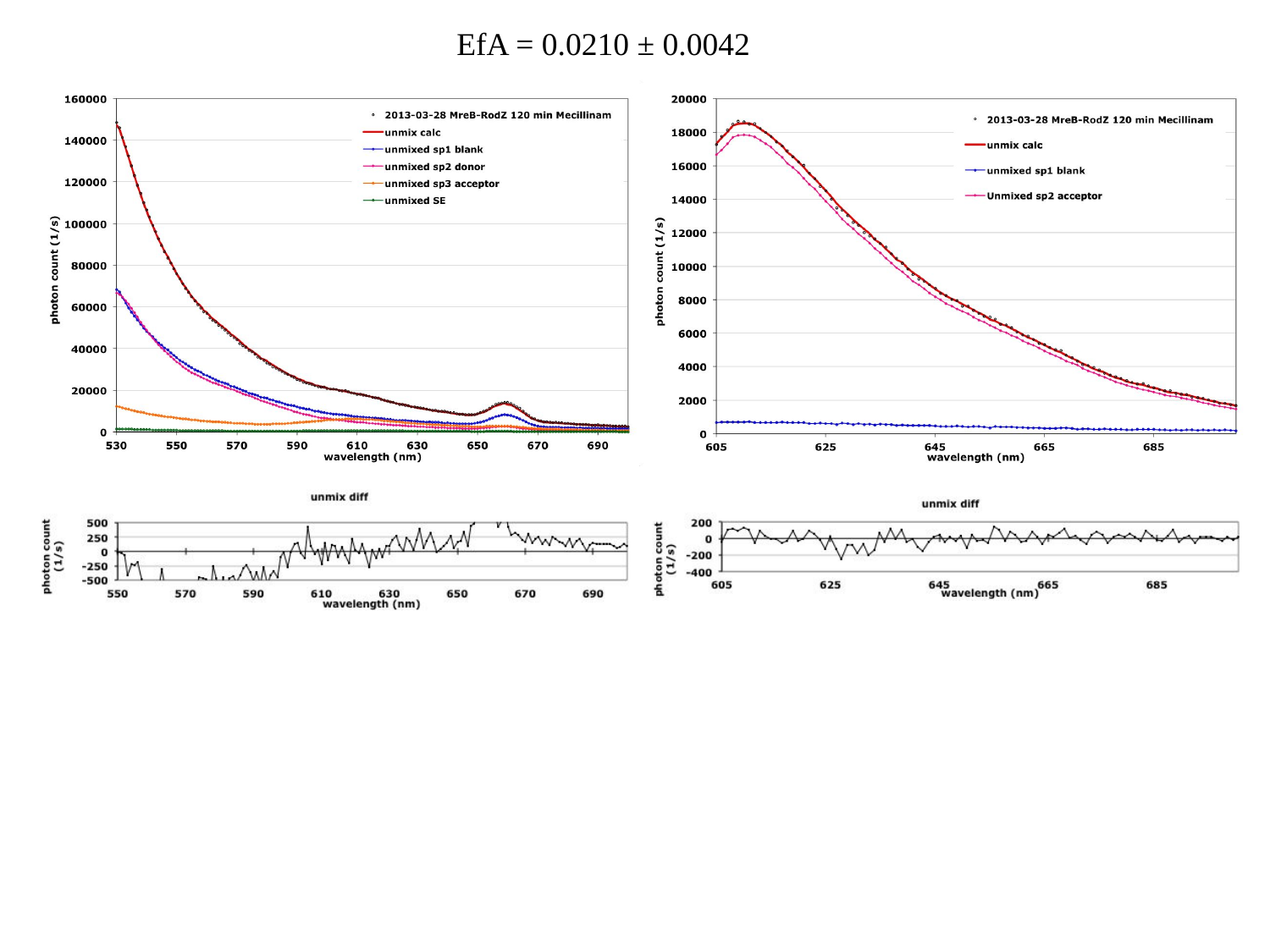

EfA = 0.0210 ± 0.0042

## Slide 56
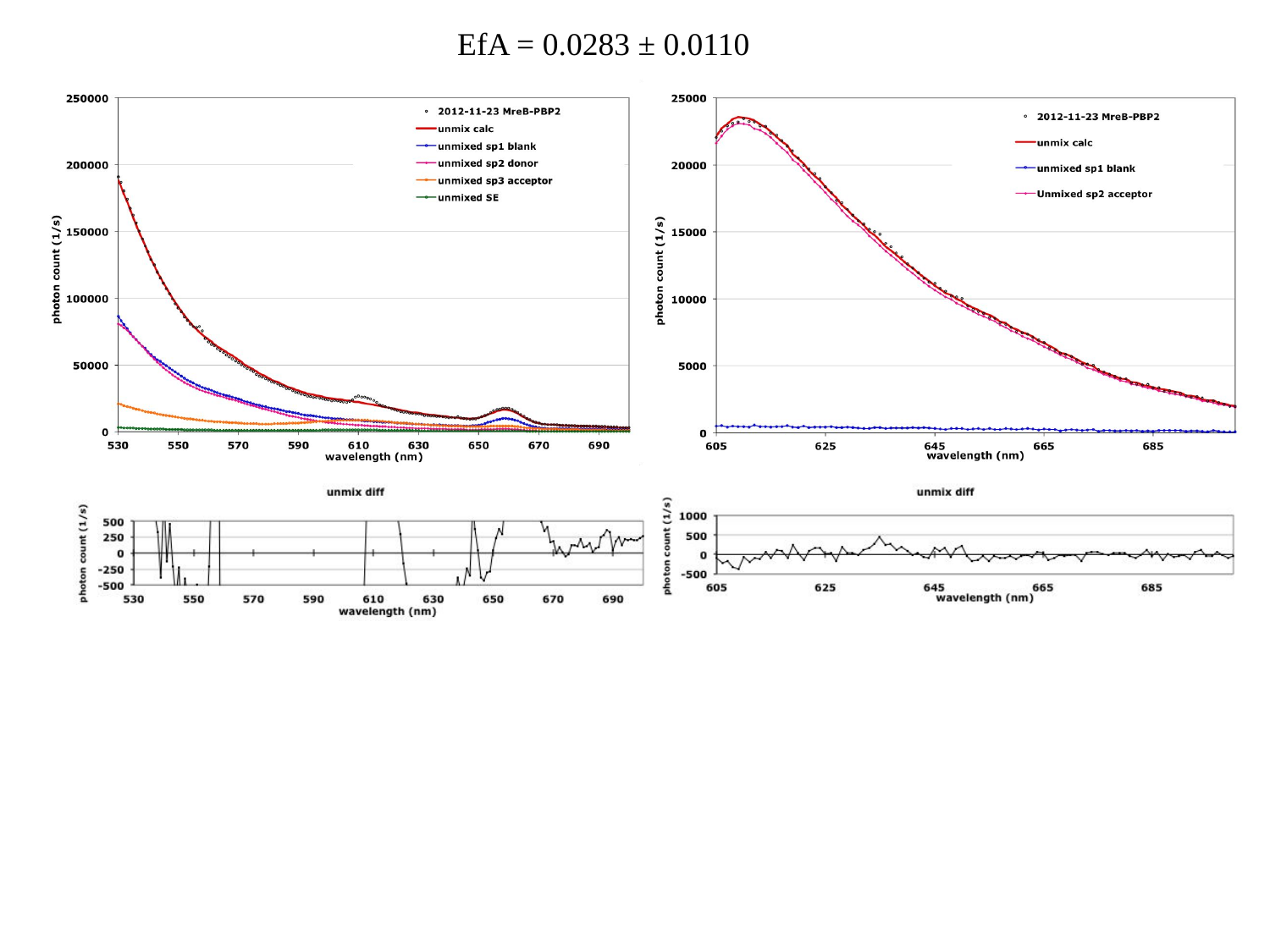

EfA = 0.0283 ± 0.0110

## Slide 57
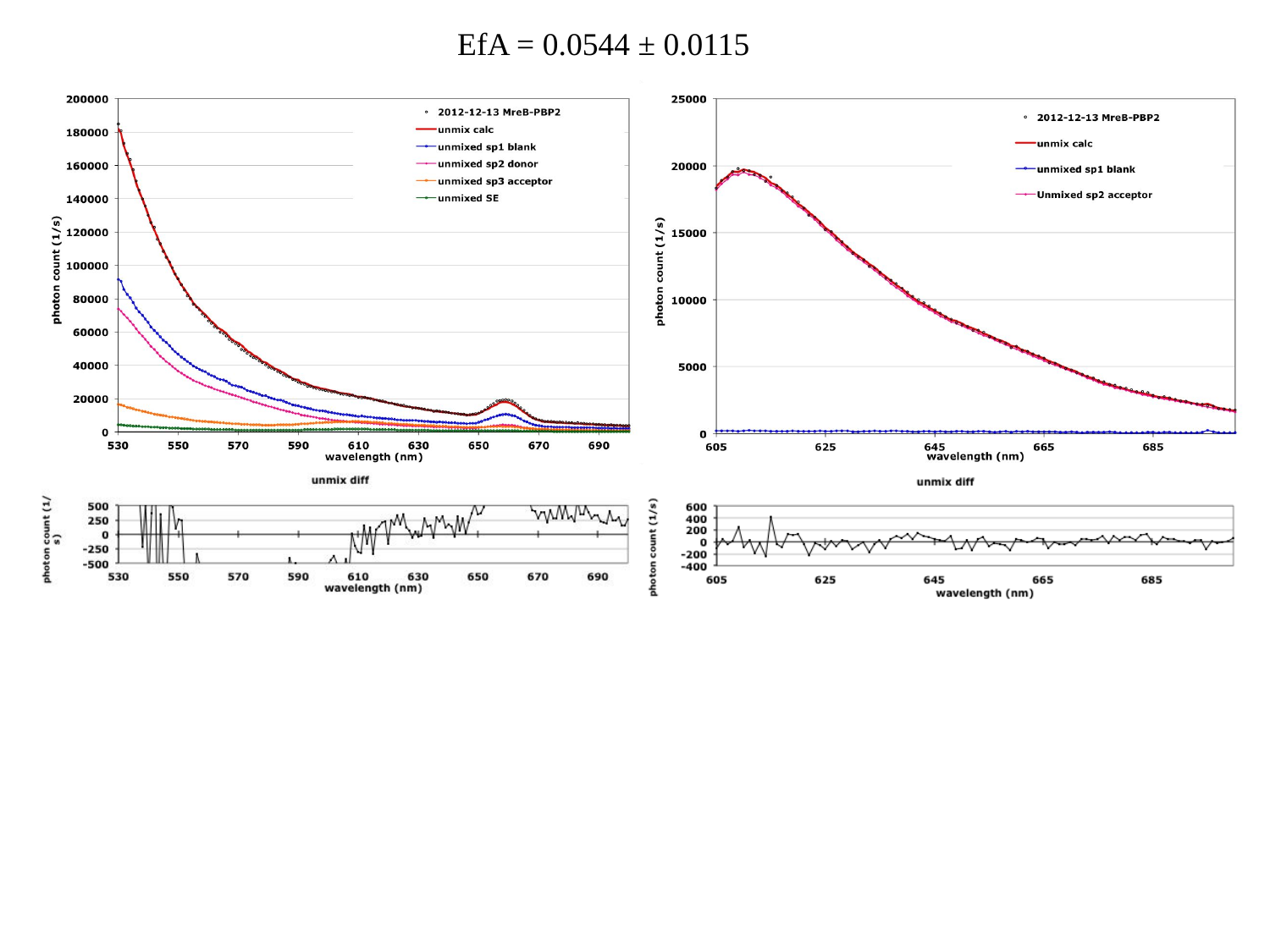

EfA = 0.0544 ± 0.0115

## Slide 58
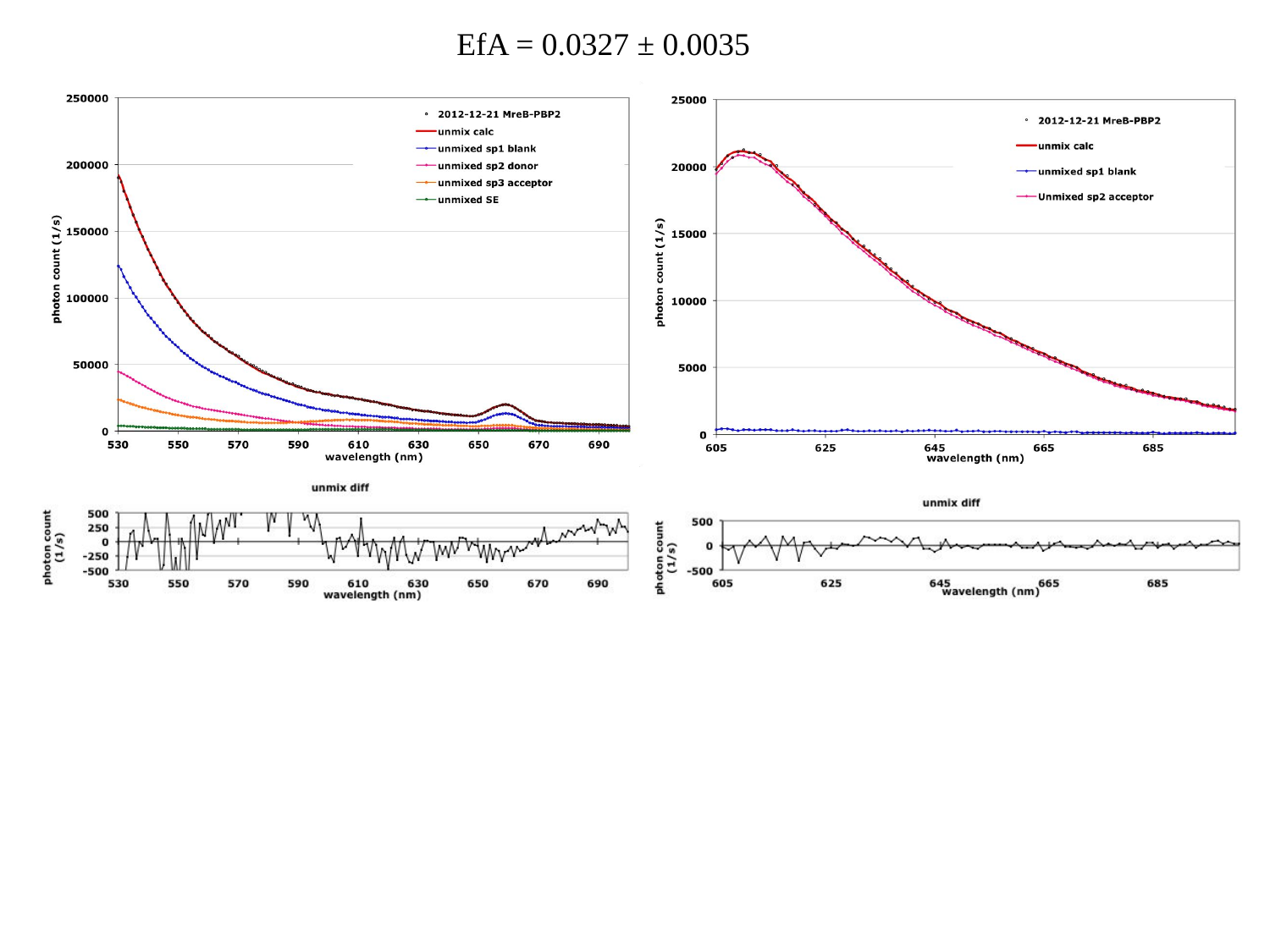

EfA = 0.0327 ± 0.0035

## Slide 59
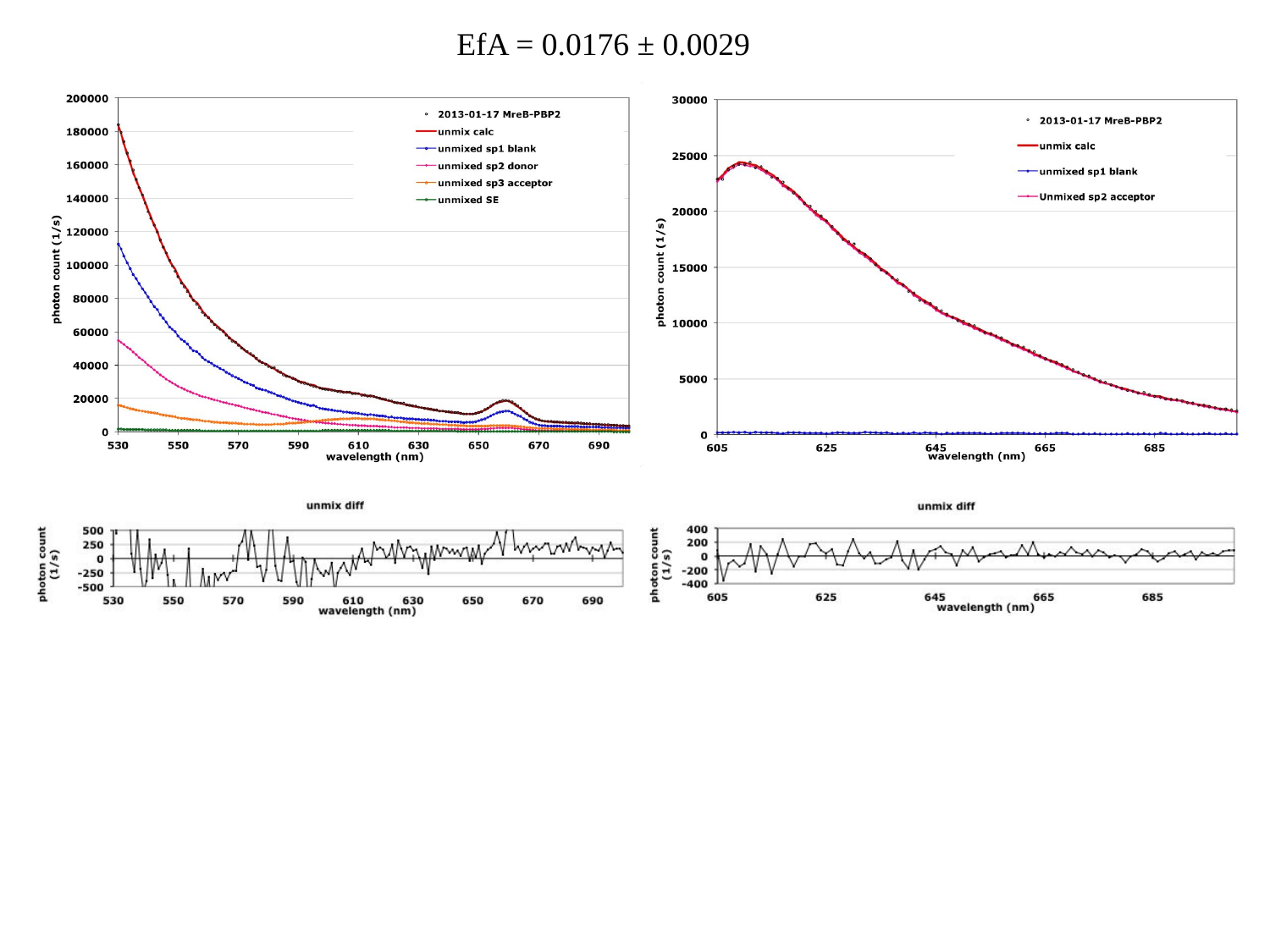

EfA = 0.0176 ± 0.0029

## Slide 60
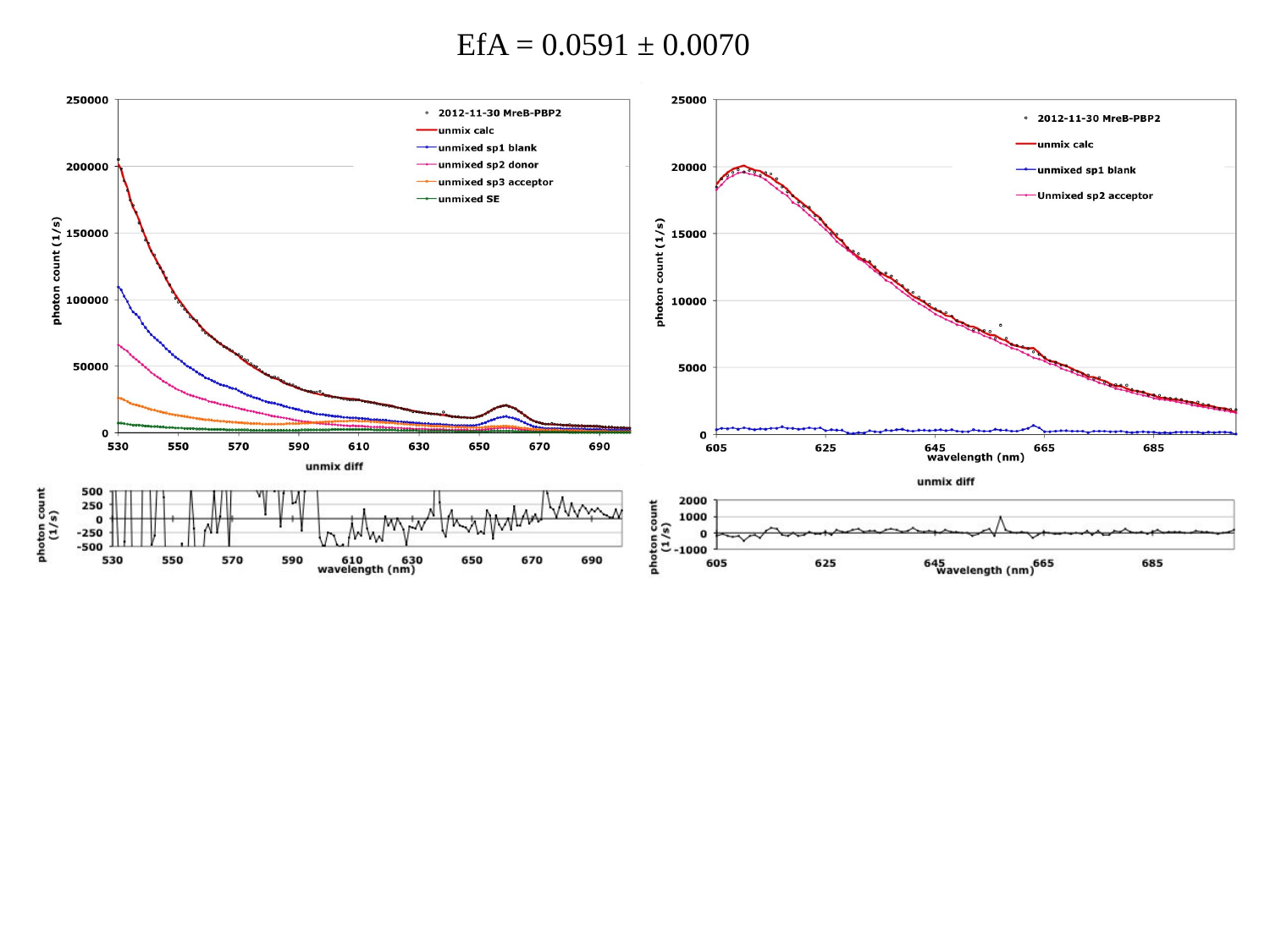

EfA = 0.0591 ± 0.0070

## Slide 61
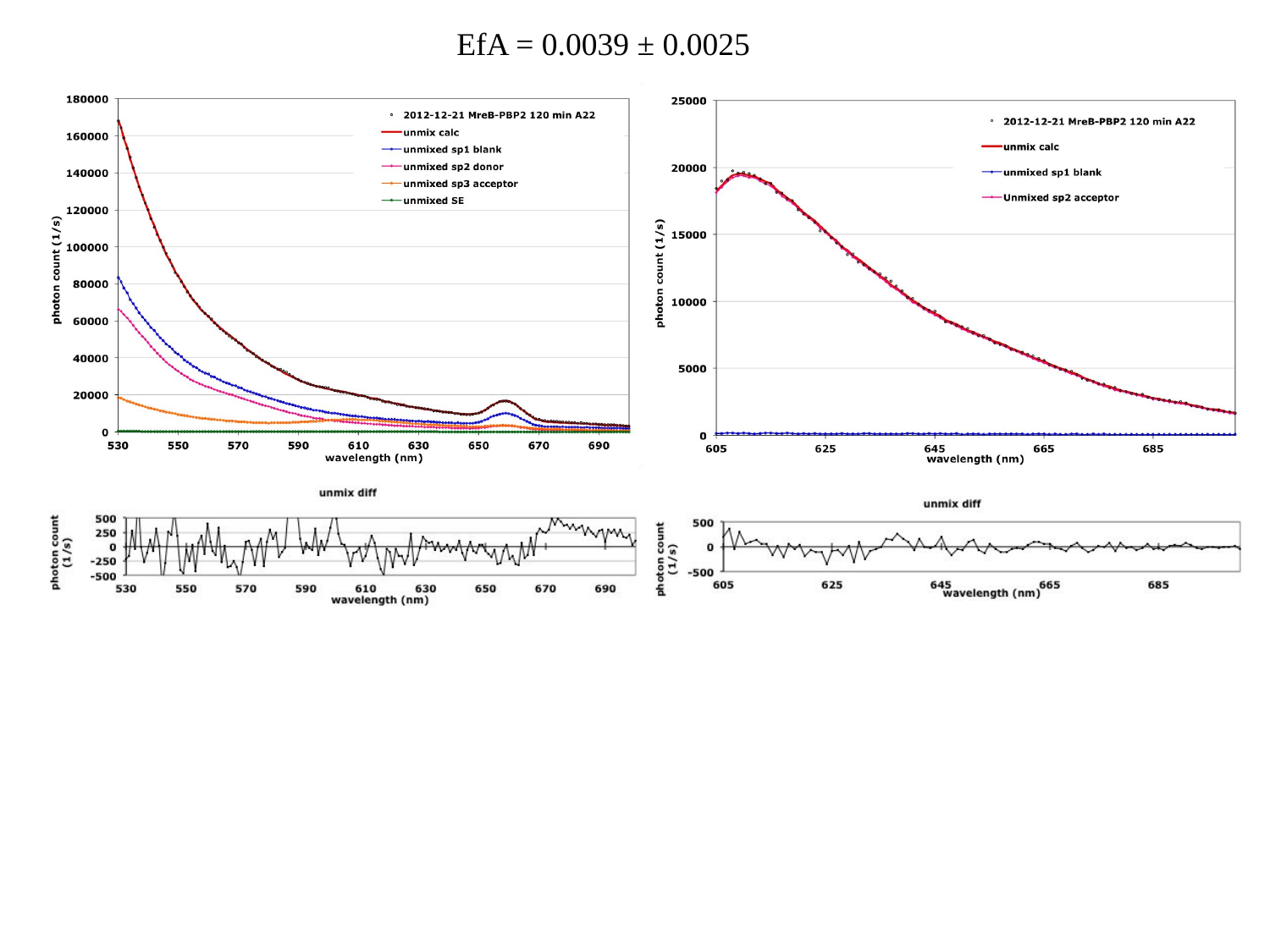

EfA = 0.0039 ± 0.0025

## Slide 62
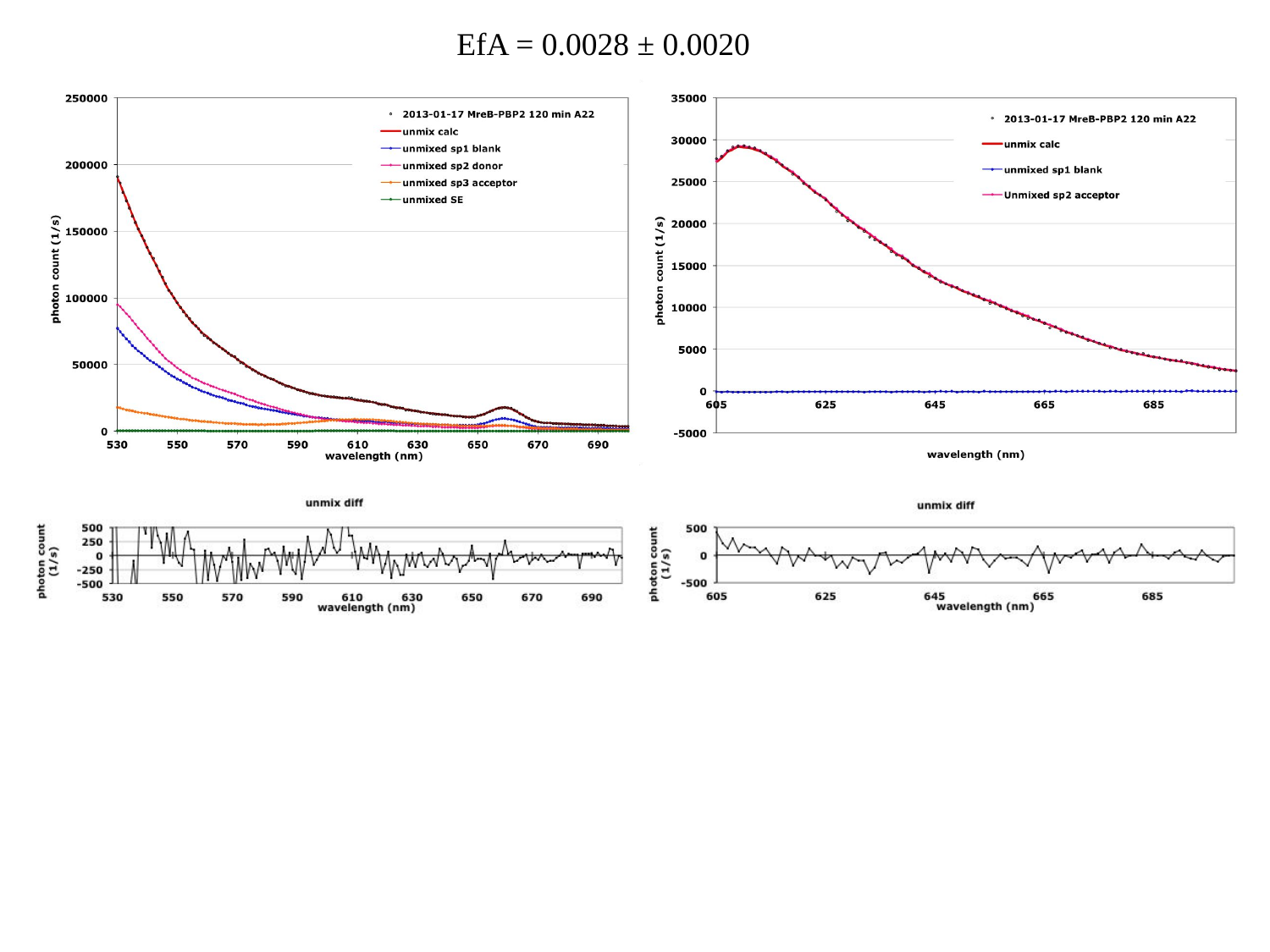

EfA = 0.0028 ± 0.0020

## Slide 63
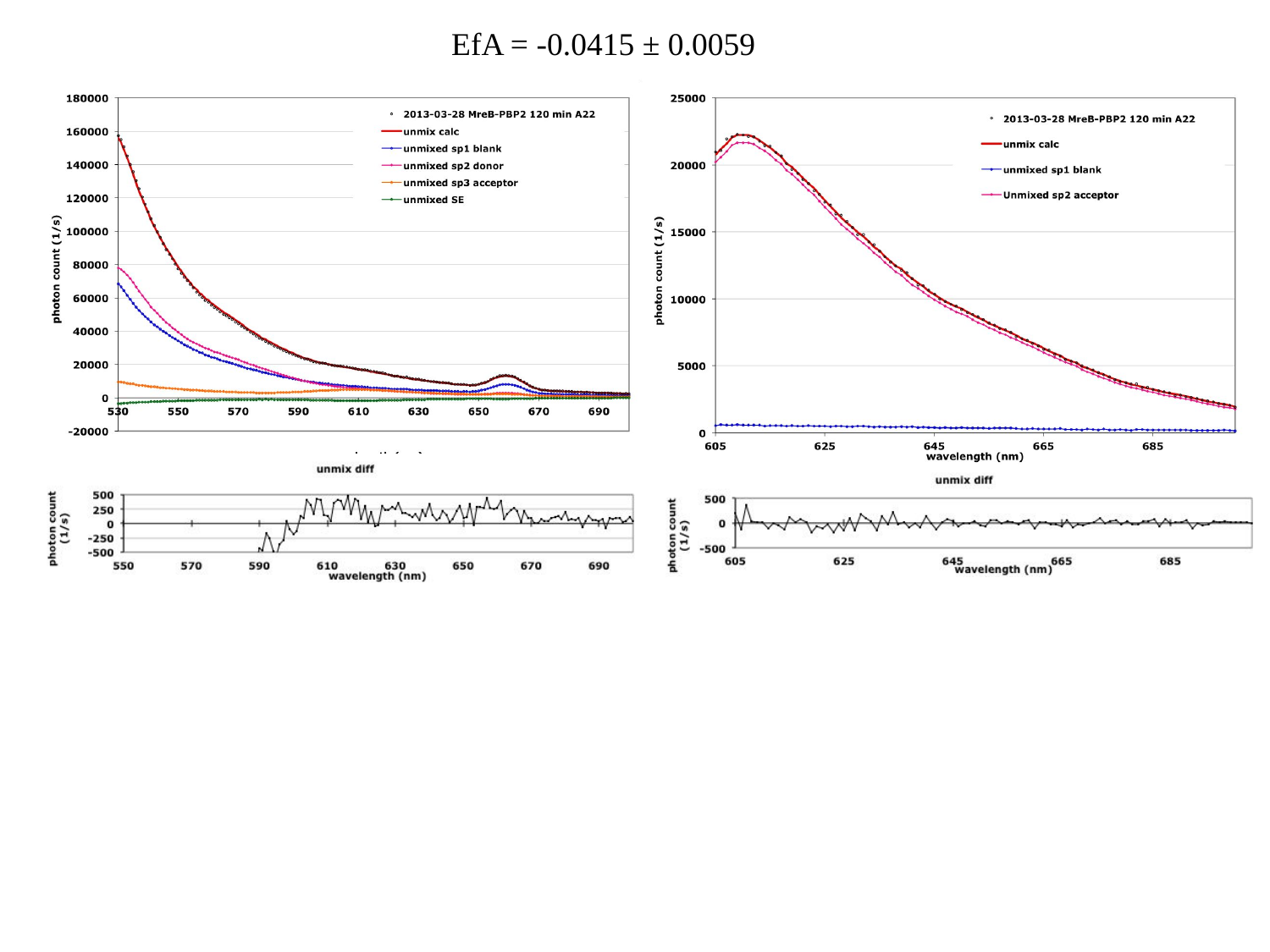

EfA = -0.0415 ± 0.0059

## Slide 64
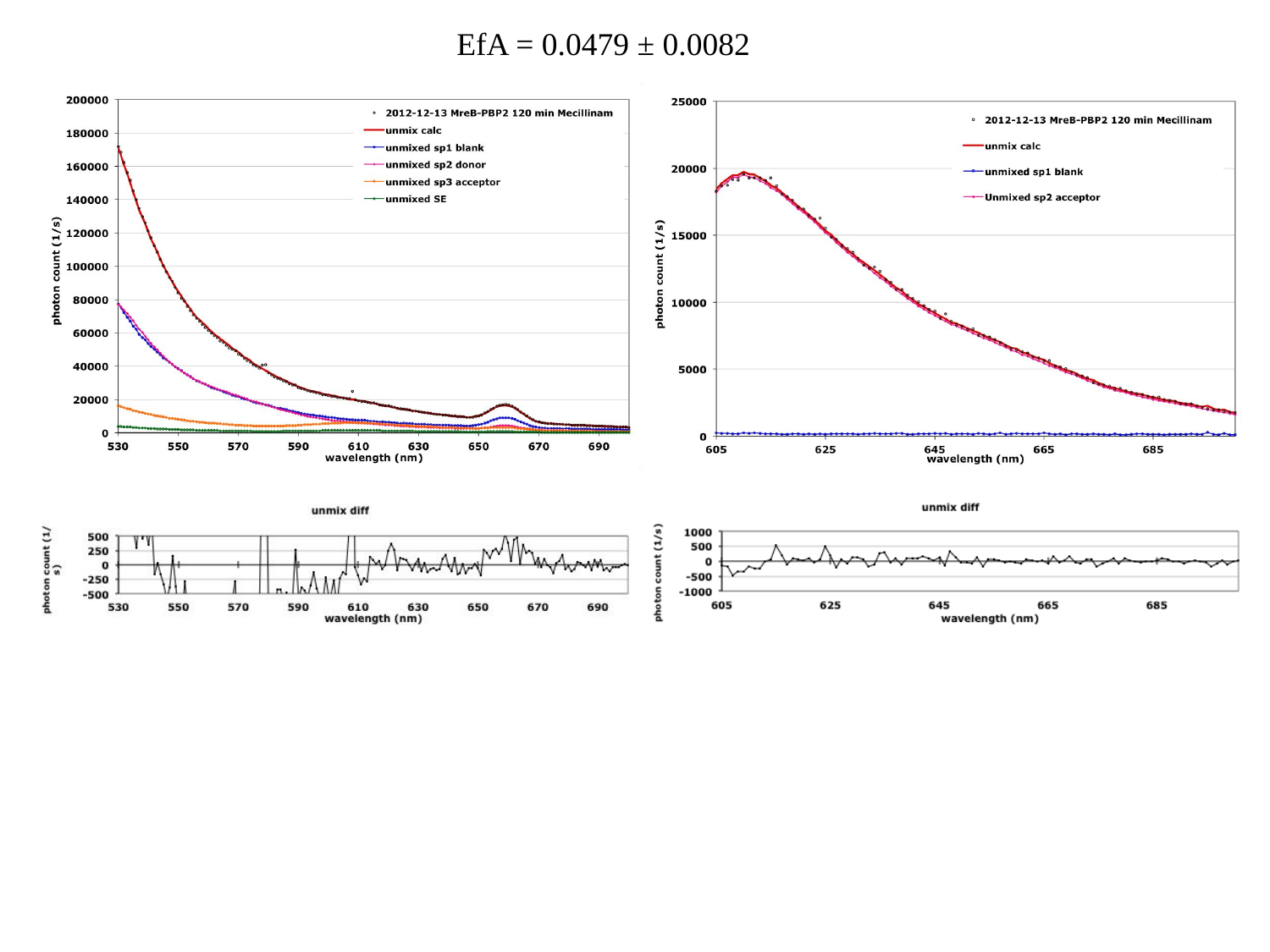

EfA = 0.0479 ± 0.0082

## Slide 65
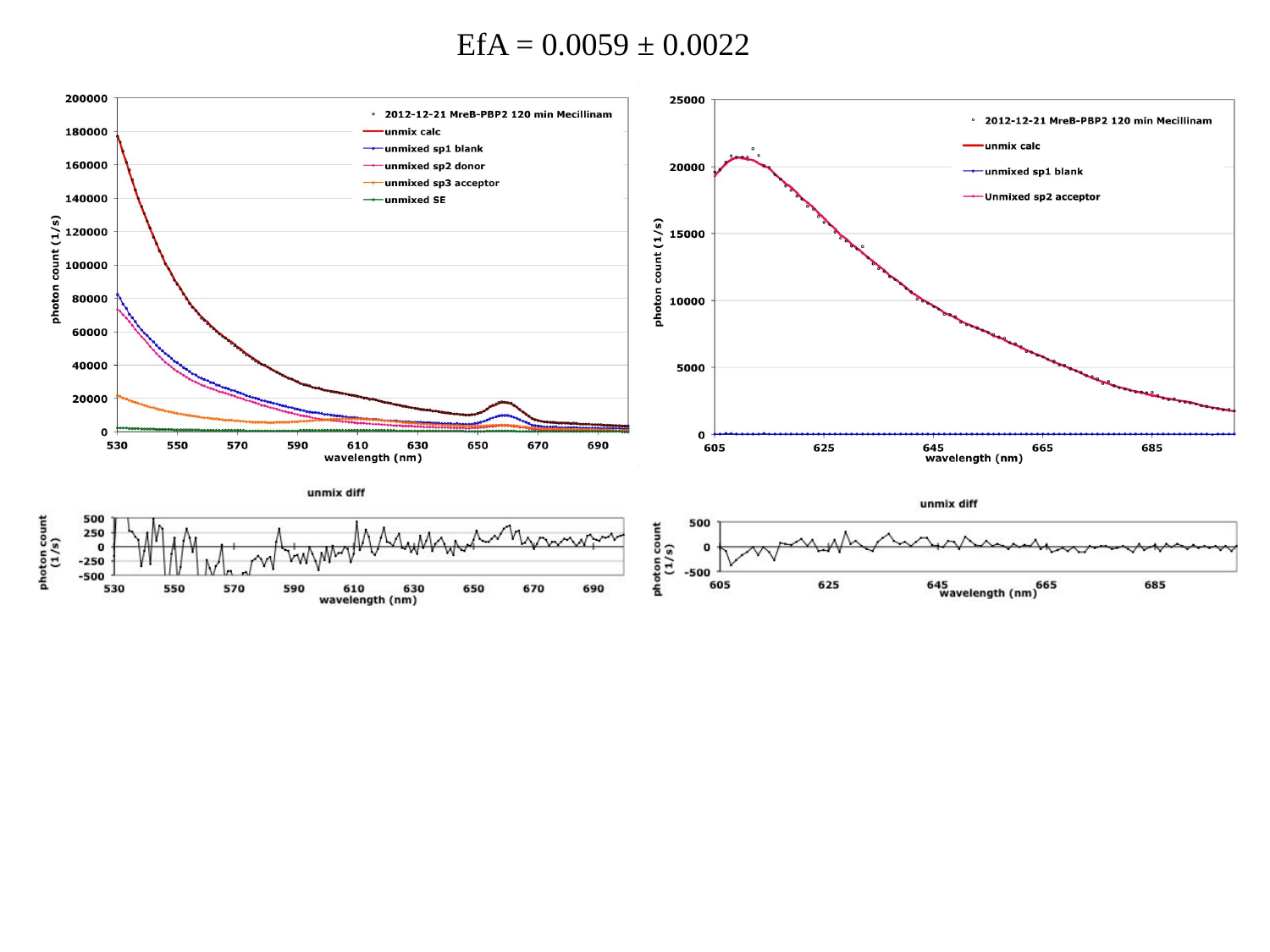

EfA = 0.0059 ± 0.0022

## Slide 66
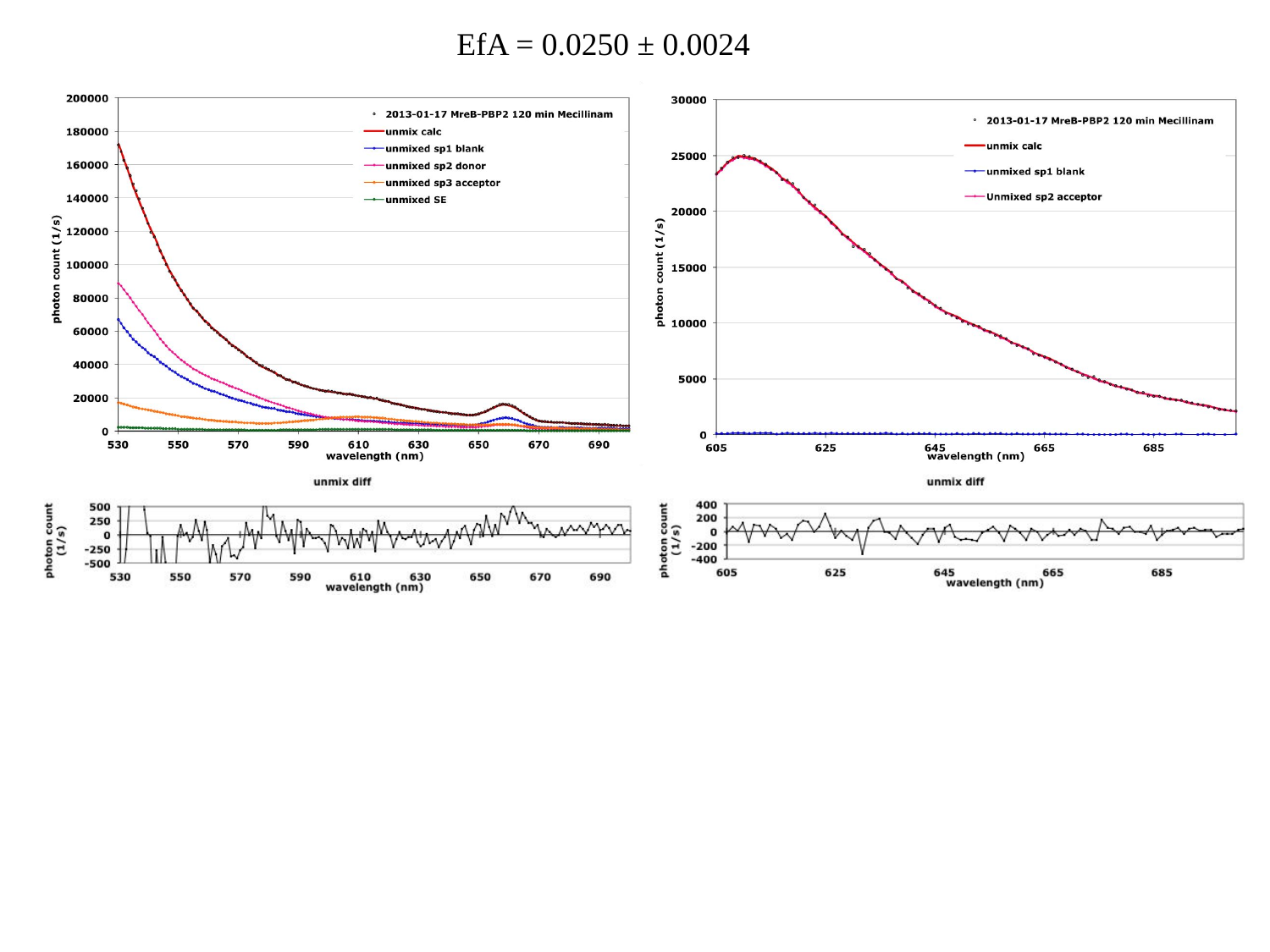

EfA = 0.0250 ± 0.0024

## Slide 67
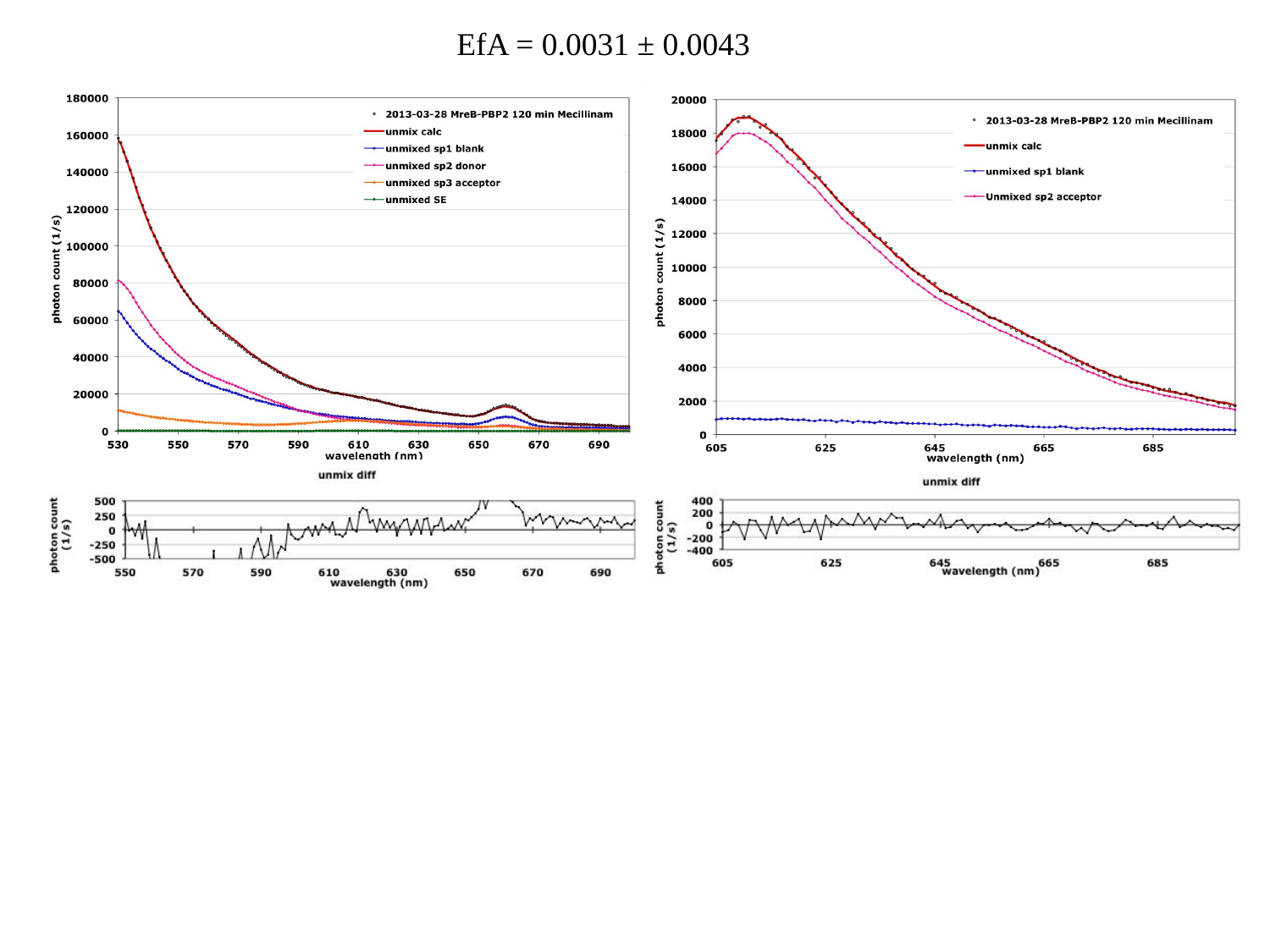

EfA = 0.0031 ± 0.0043

## Slide 68
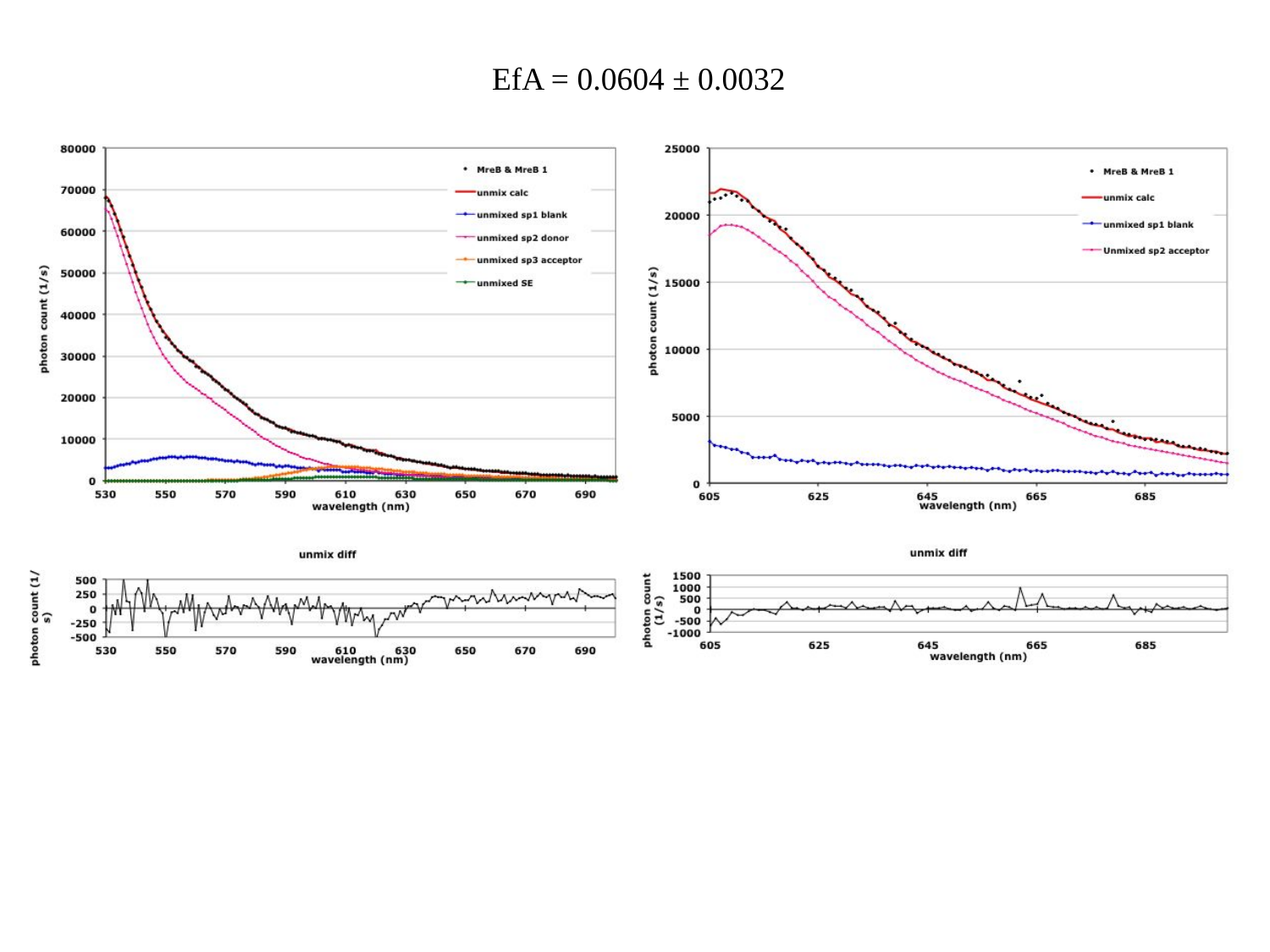

EfA = 0.0604 ± 0.0032

## Slide 69
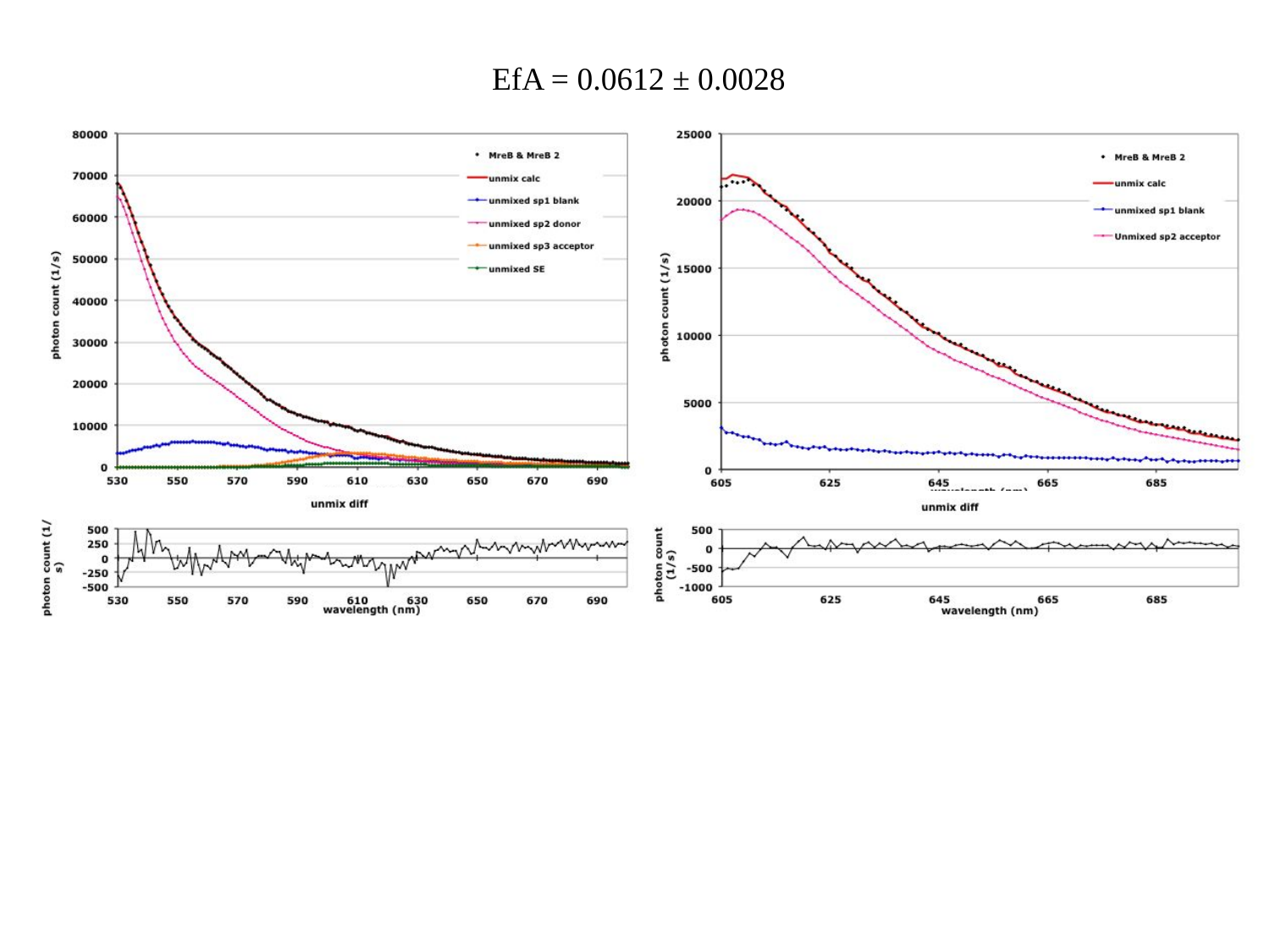

EfA = 0.0612 ± 0.0028

## Slide 70
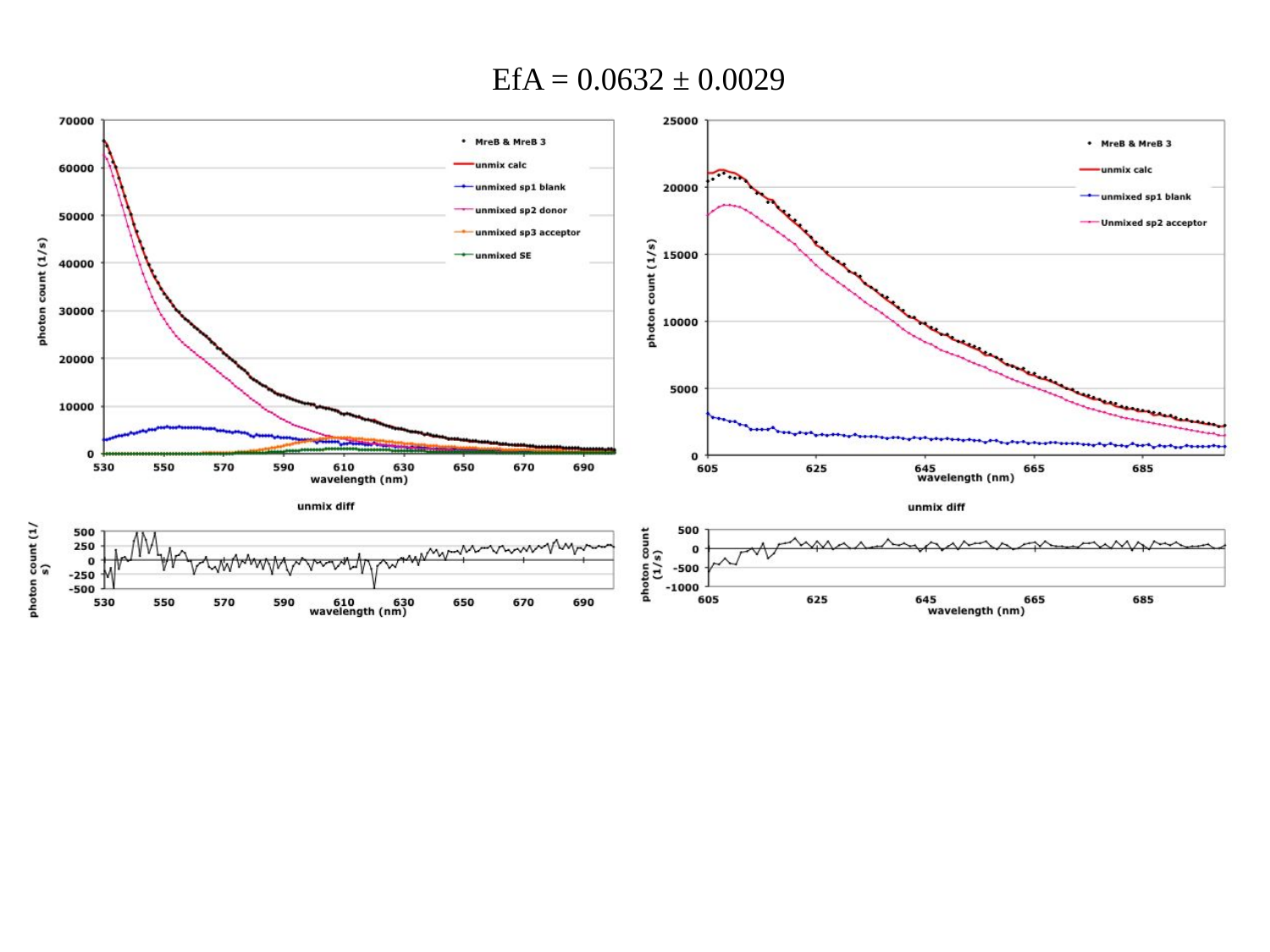

EfA = 0.0632 ± 0.0029

## Slide 71
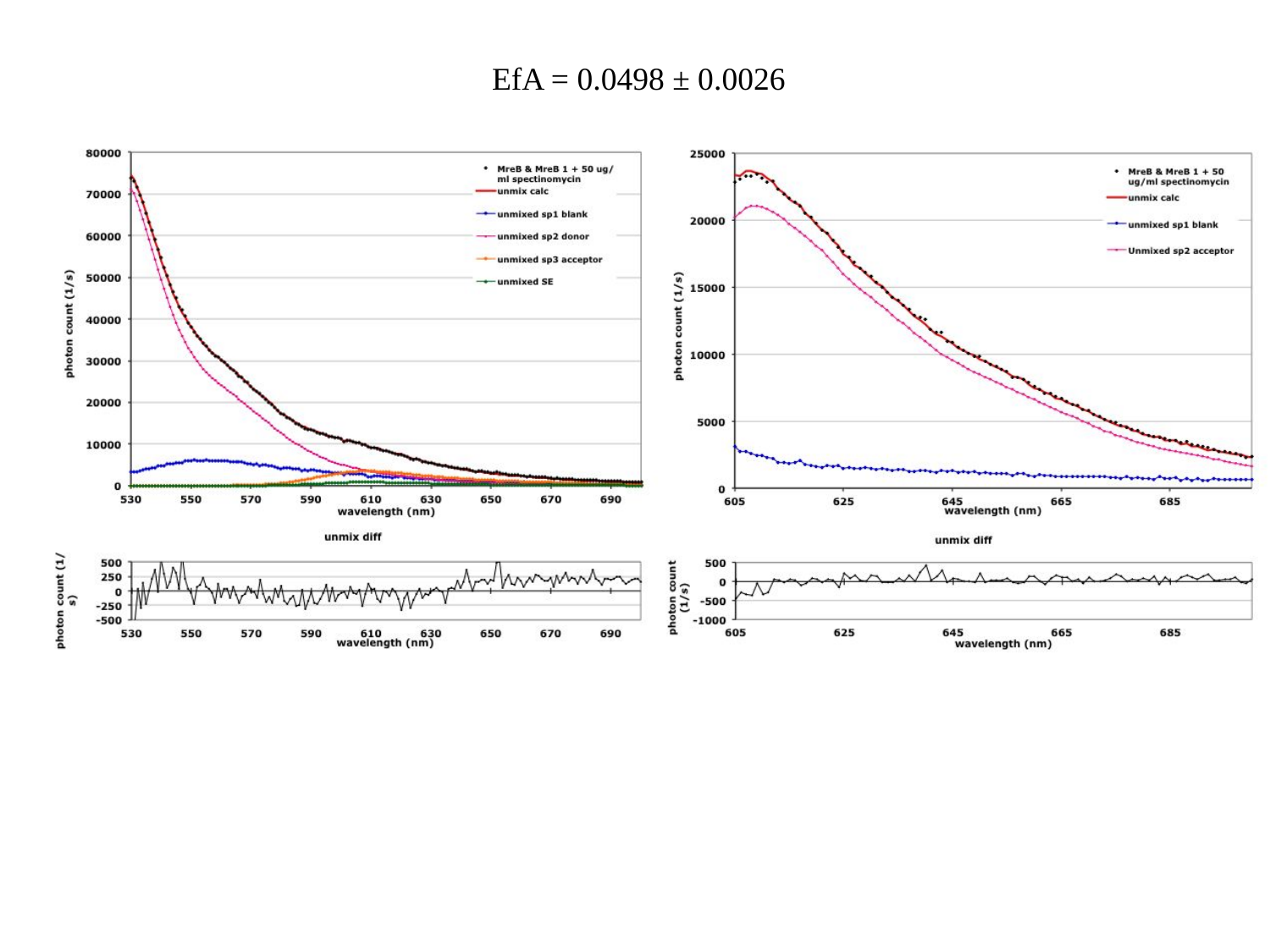

EfA = 0.0498 ± 0.0026

## Slide 72
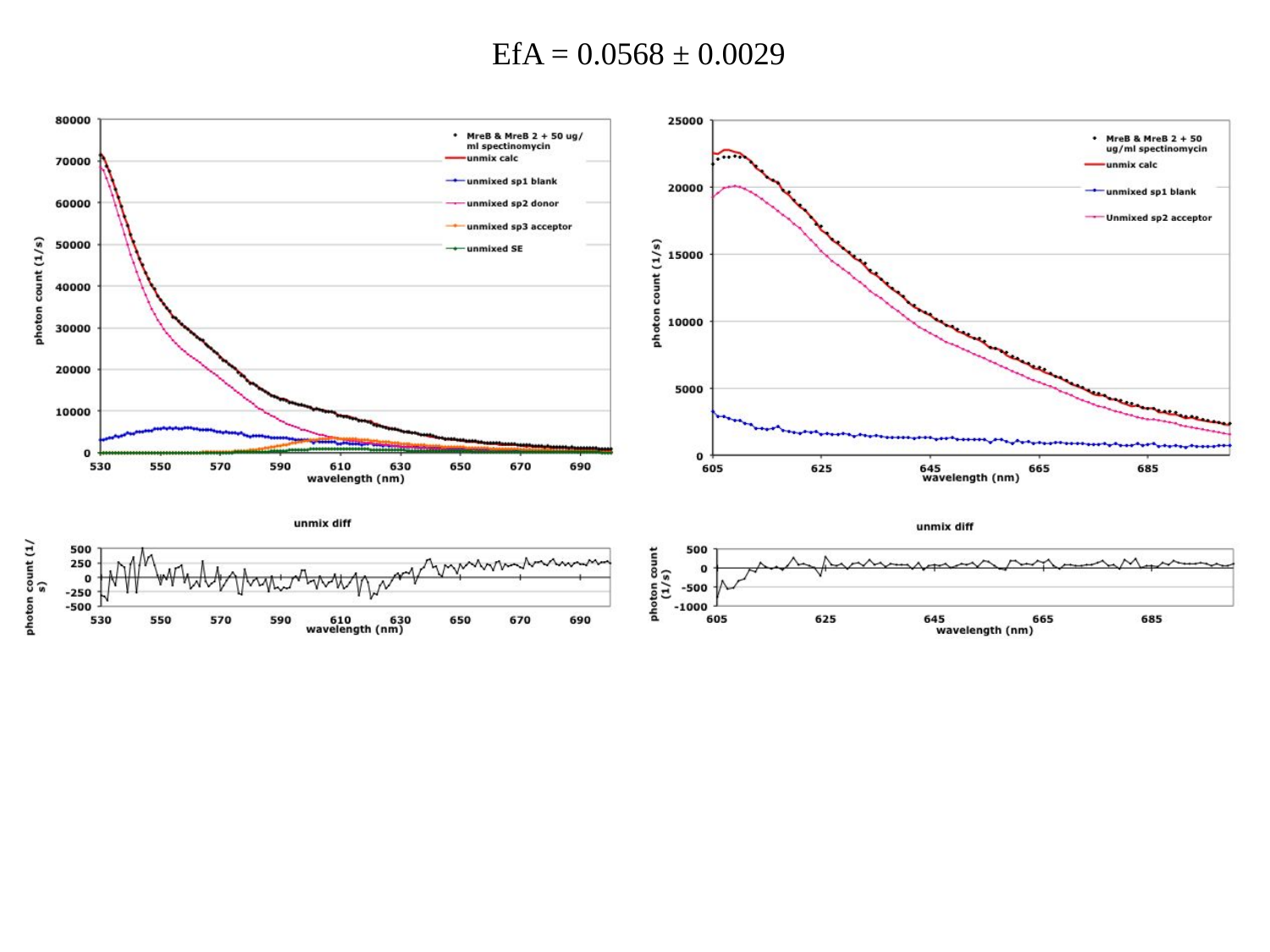

EfA = 0.0568 ± 0.0029

## Slide 73
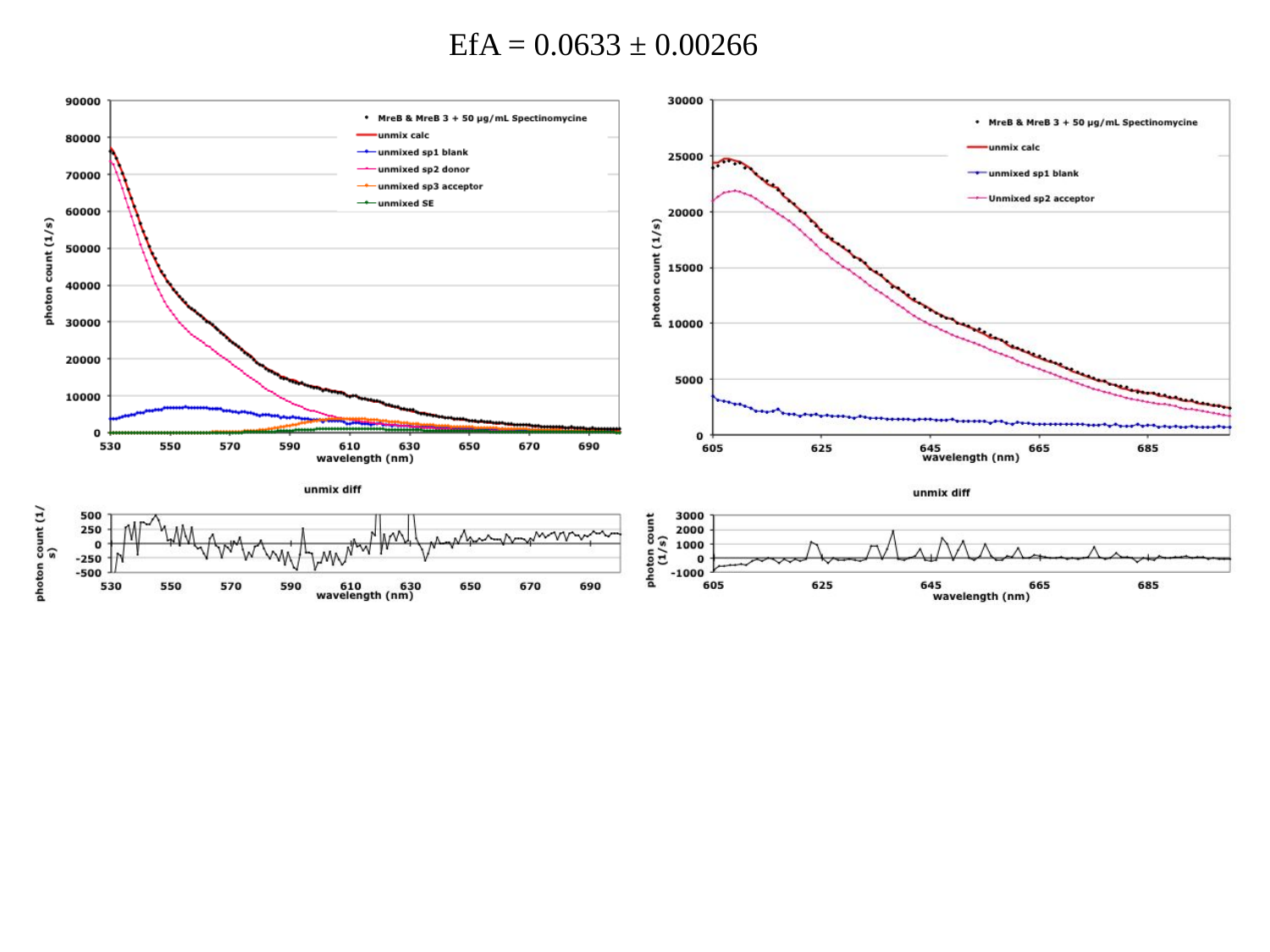

EfA = 0.0633 ± 0.00266

## Slide 74
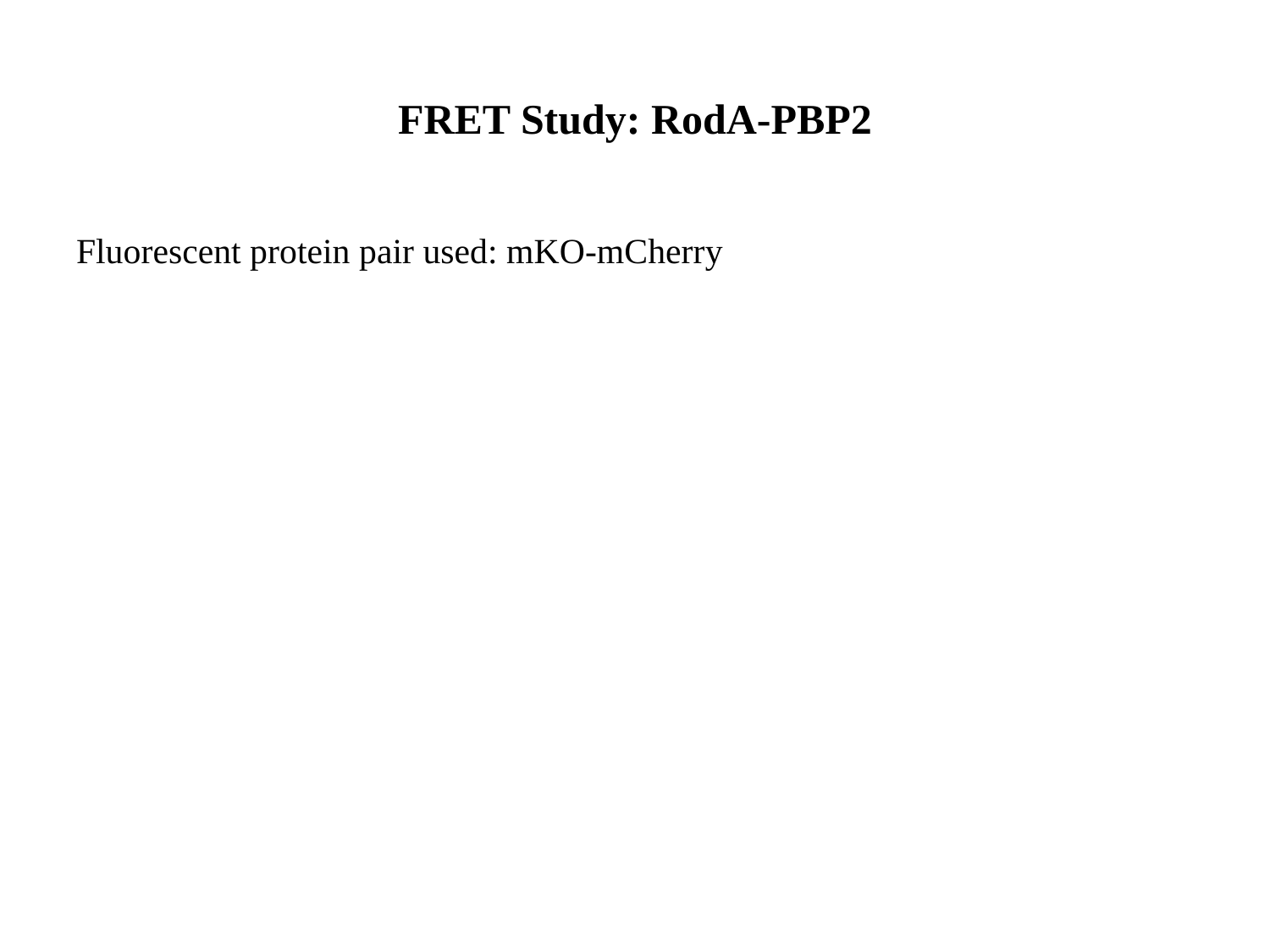

# FRET Study: RodA-PBP2
Fluorescent protein pair used: mKO-mCherry

## Slide 75
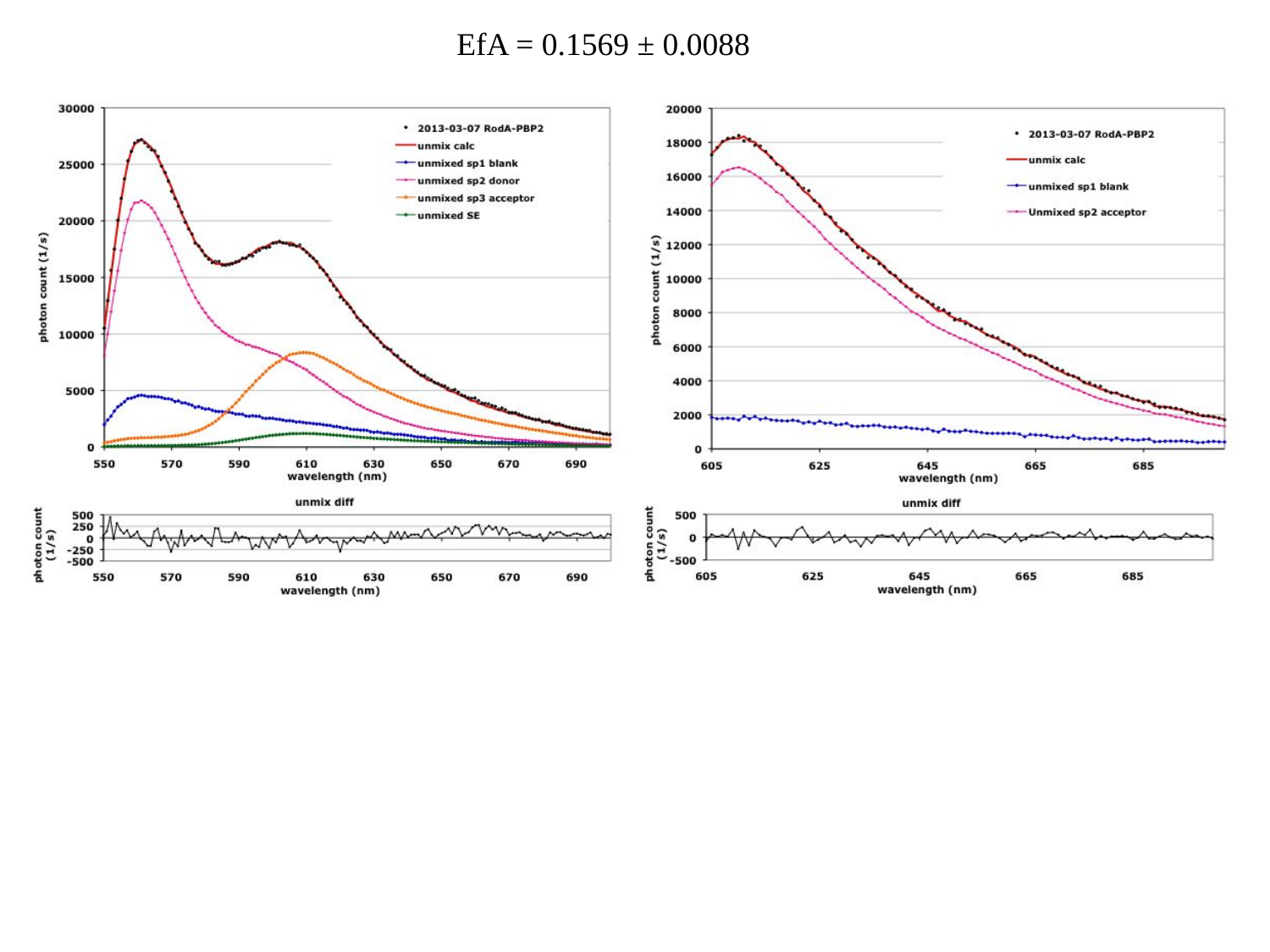

EfA = 0.1569 ± 0.0088

## Slide 76
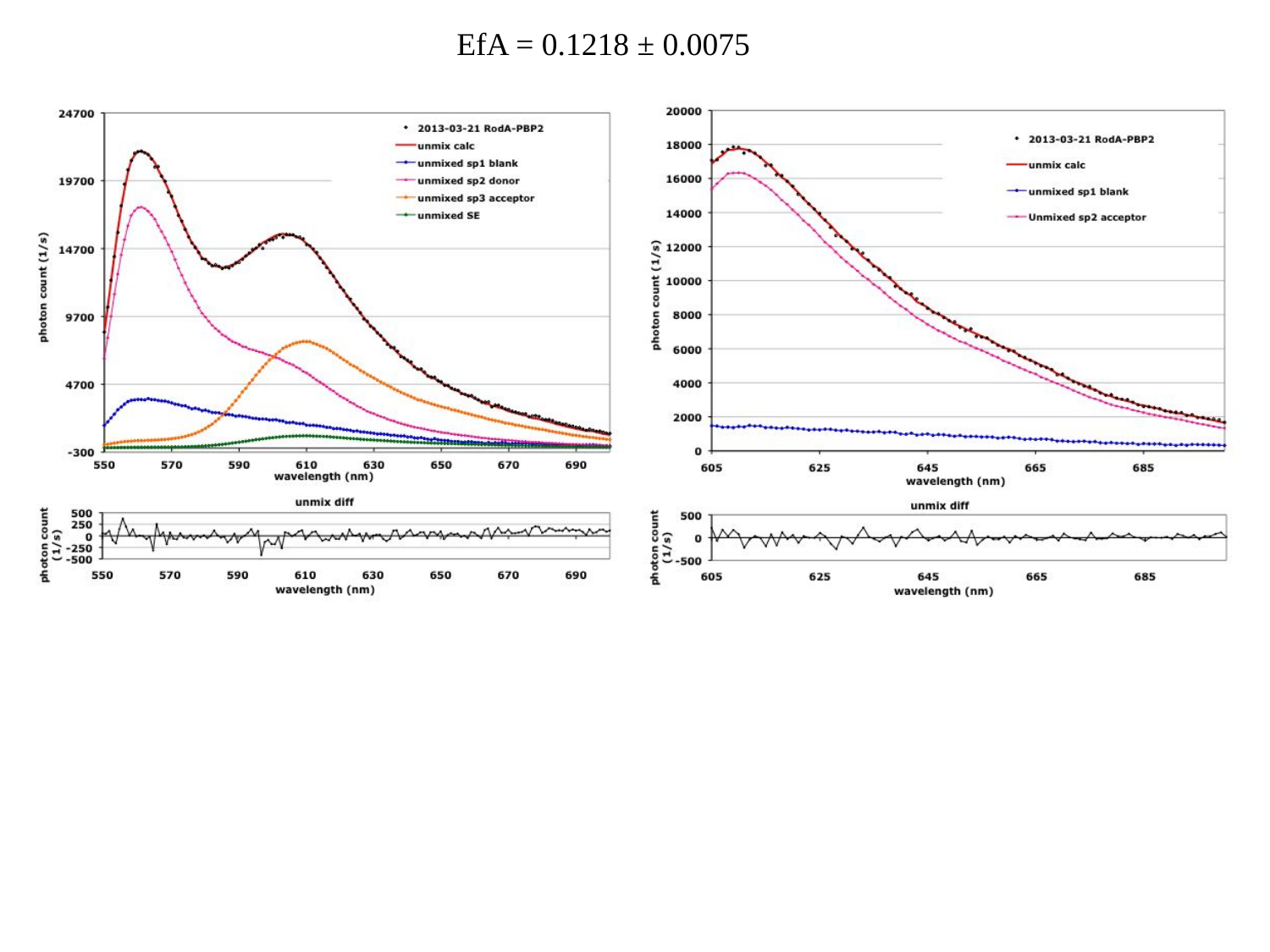

EfA = 0.1218 ± 0.0075

## Slide 77
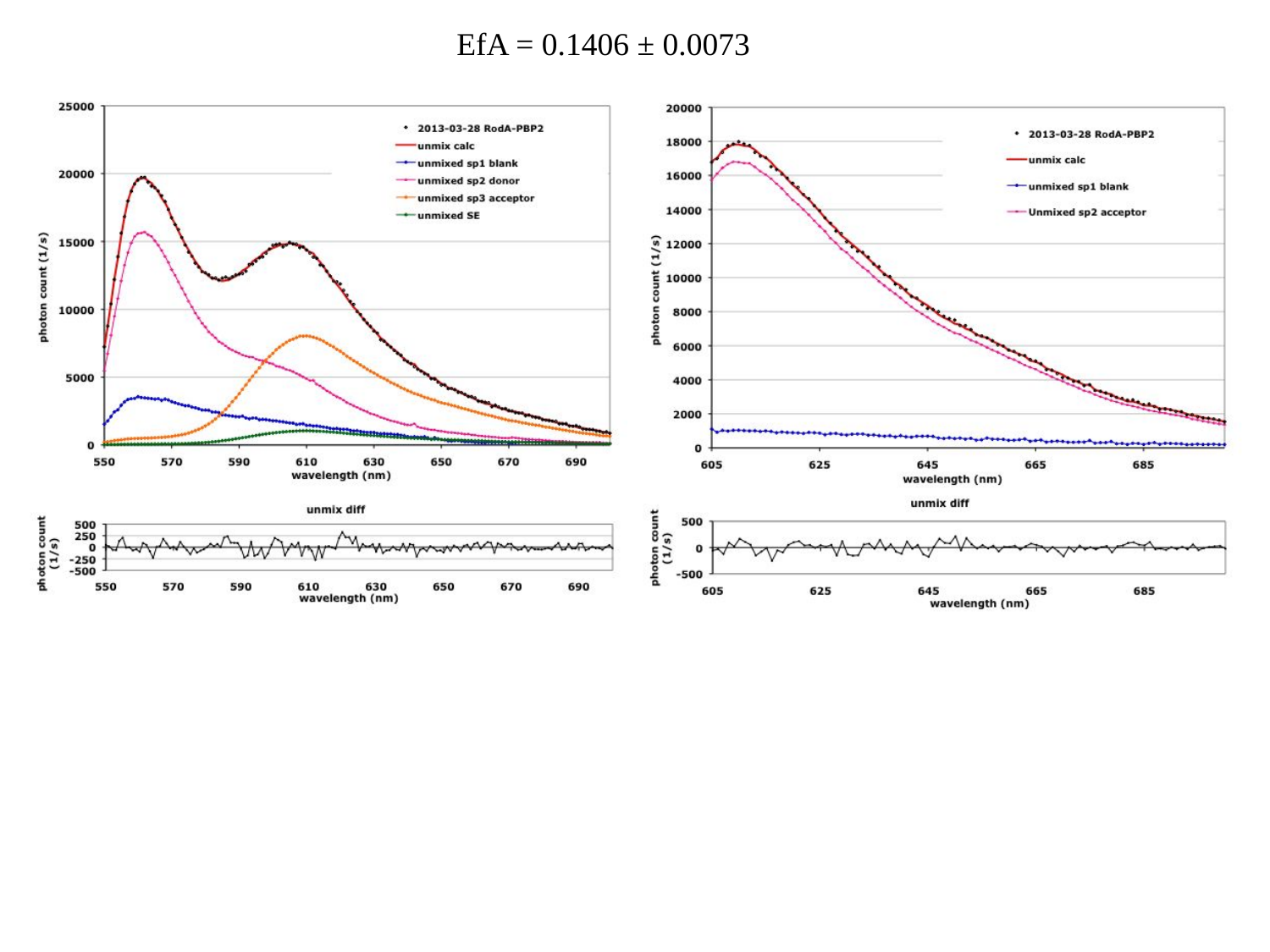

EfA = 0.1406 ± 0.0073

## Slide 78
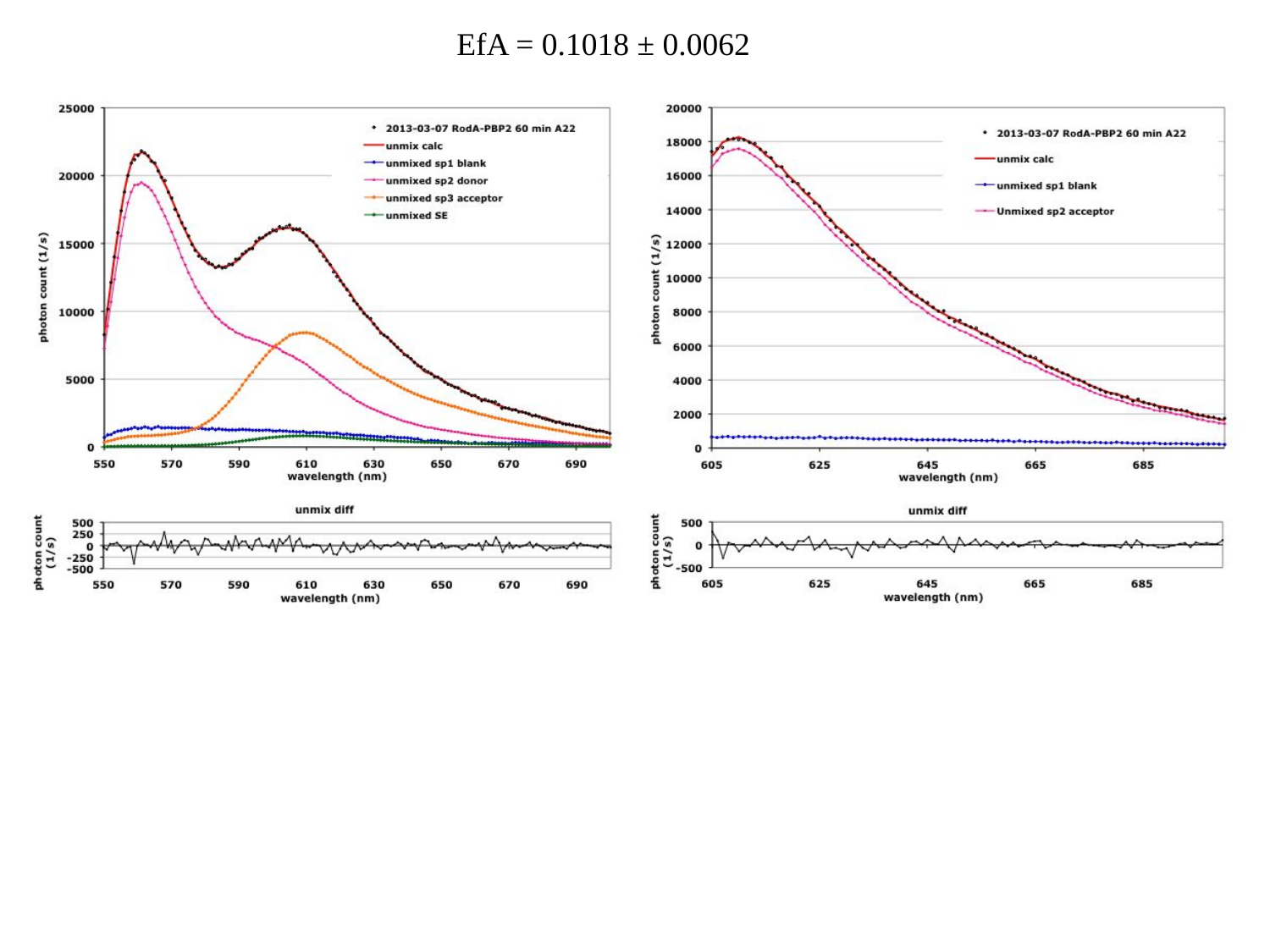

EfA = 0.1018 ± 0.0062

## Slide 79
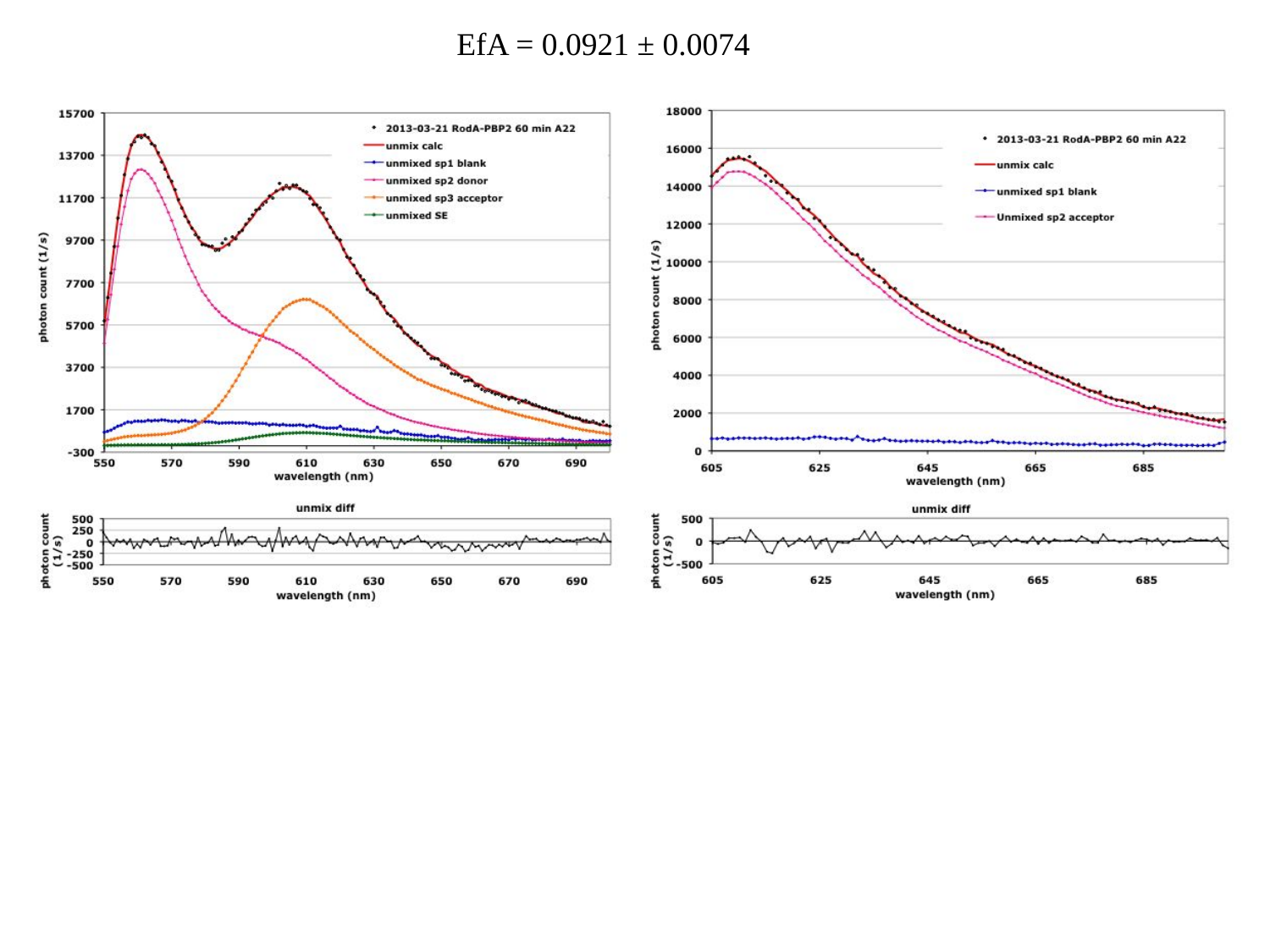

EfA = 0.0921 ± 0.0074

## Slide 80
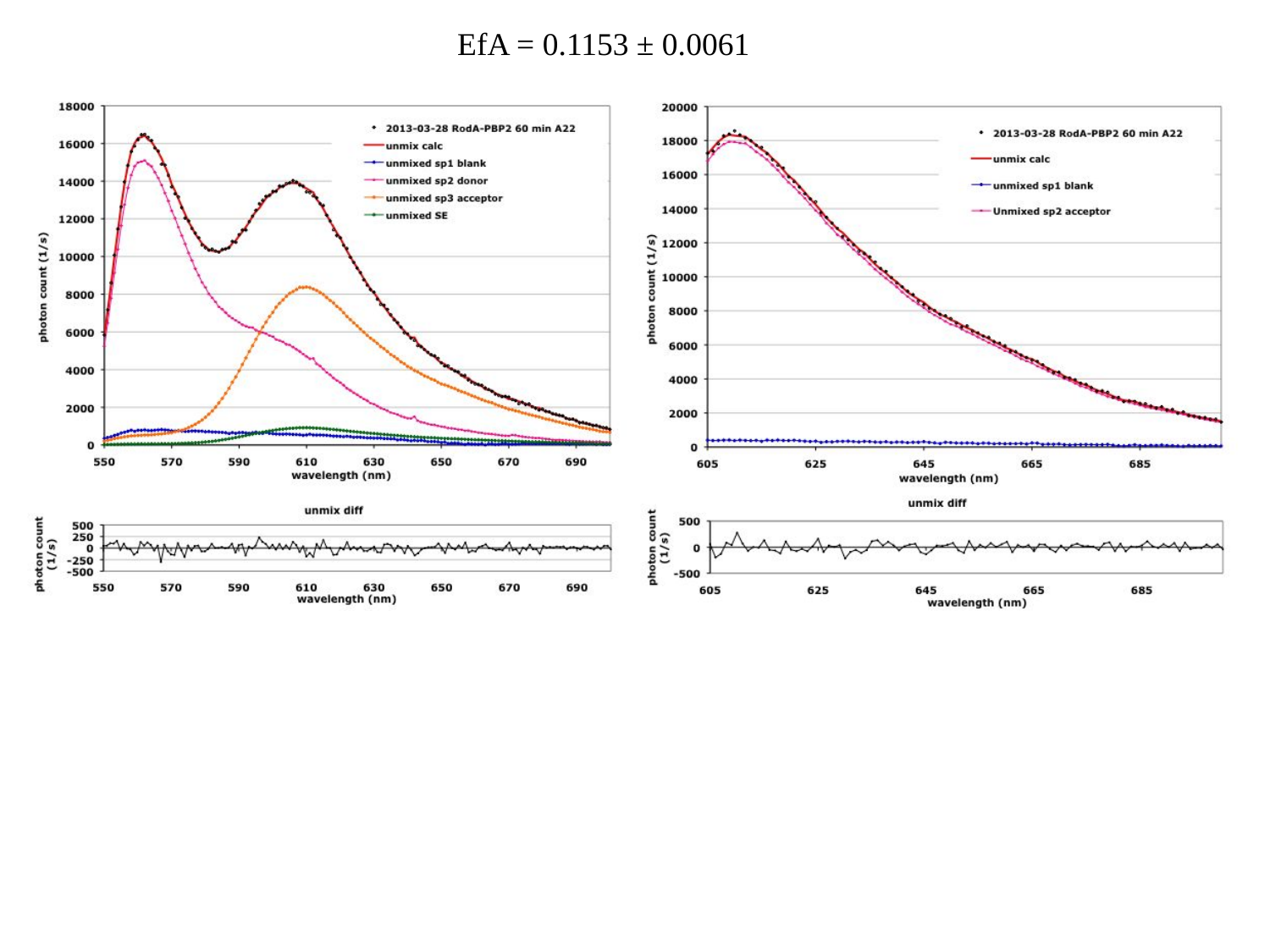

EfA = 0.1153 ± 0.0061

## Slide 81
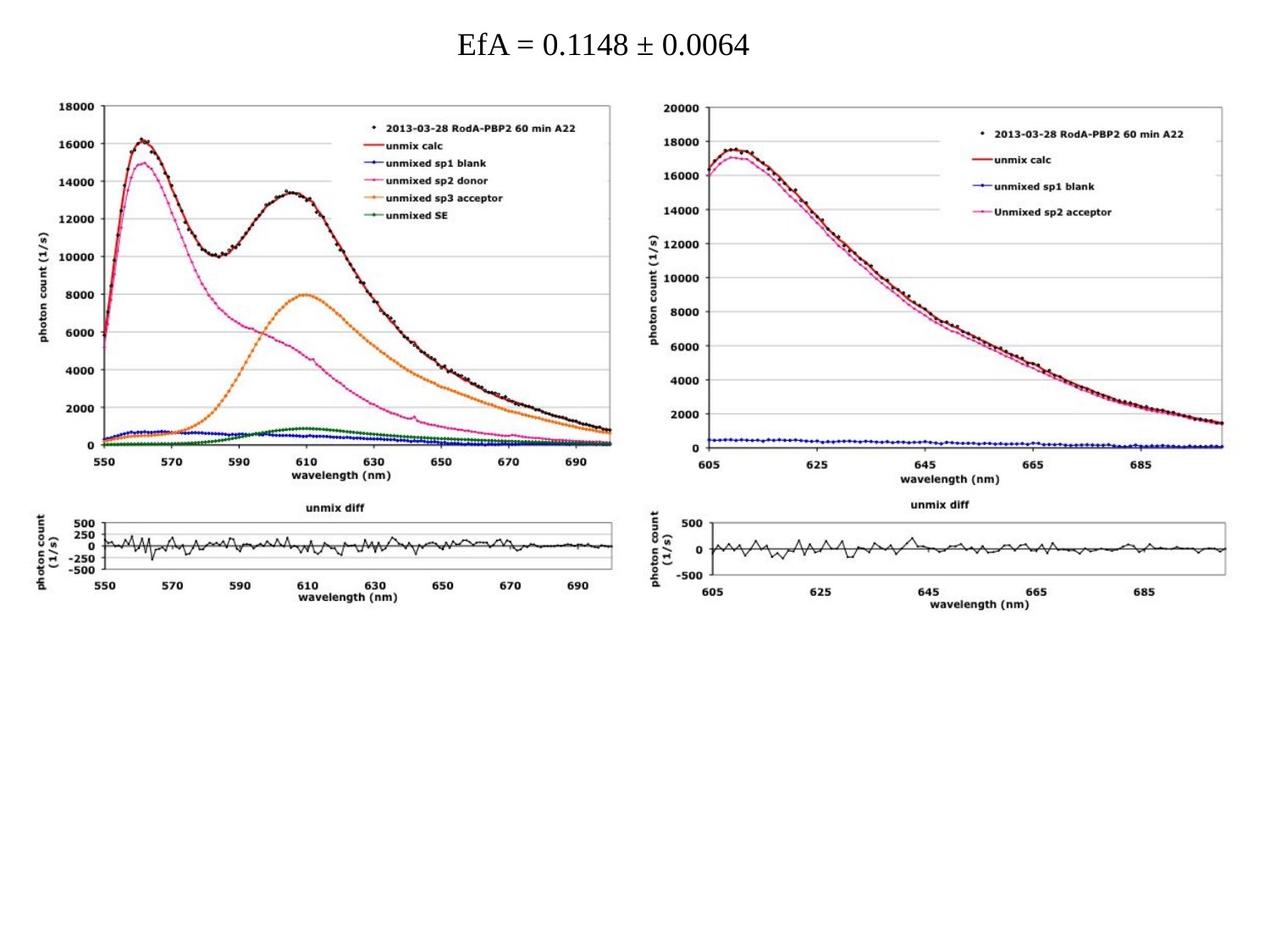

EfA = 0.1148 ± 0.0064

## Slide 82
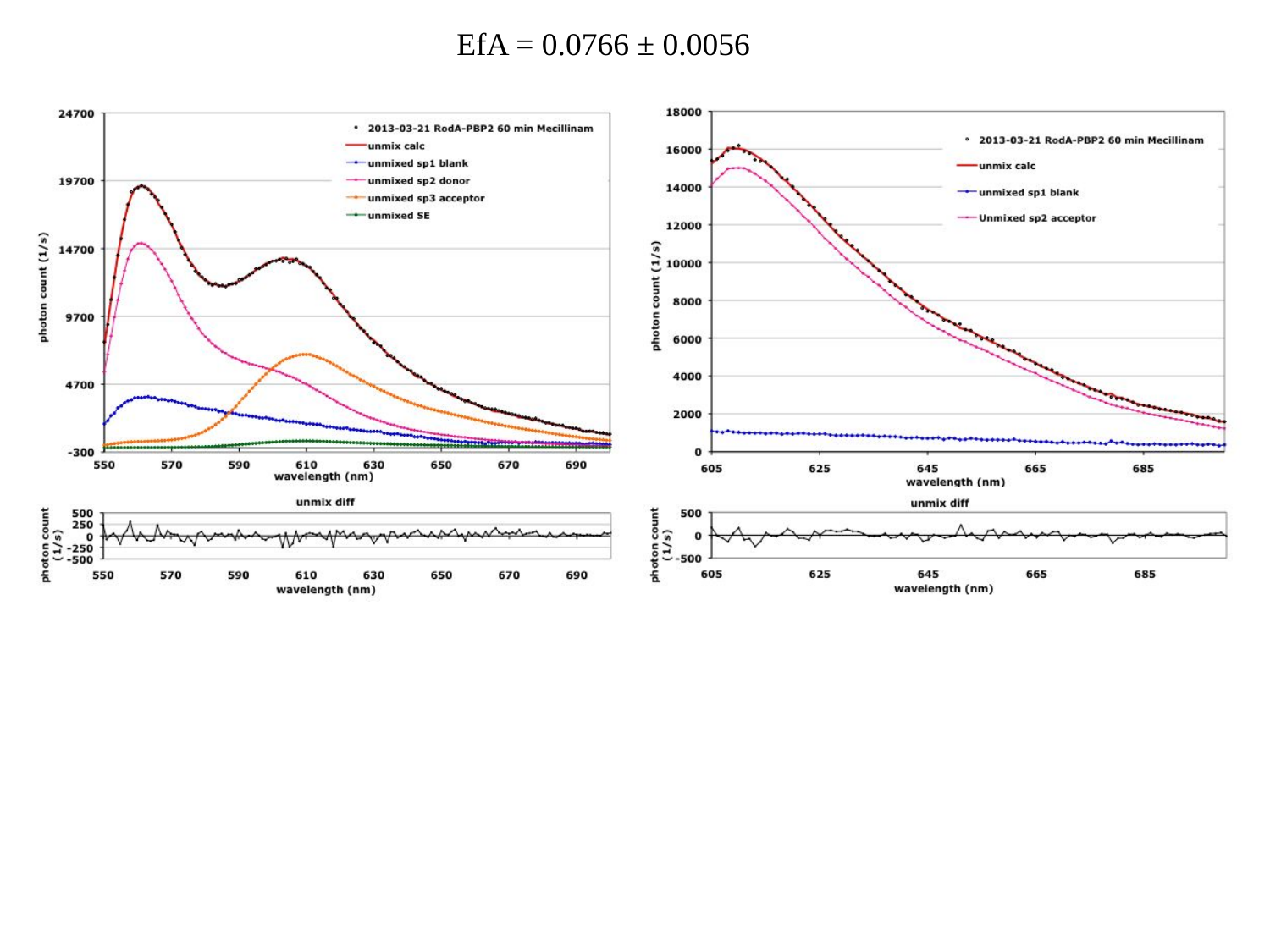

EfA = 0.0766 ± 0.0056

## Slide 83
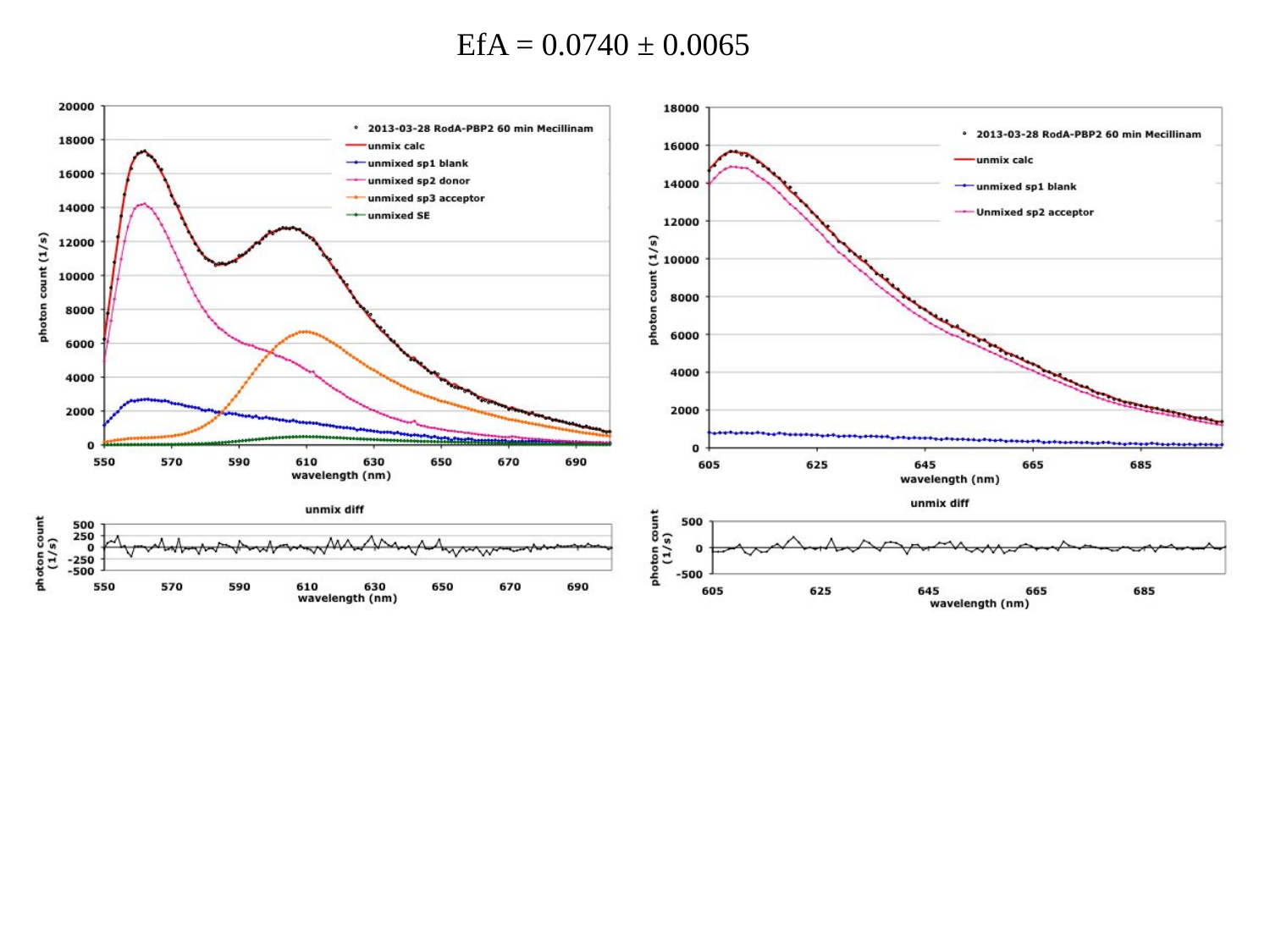

EfA = 0.0740 ± 0.0065

## Slide 84
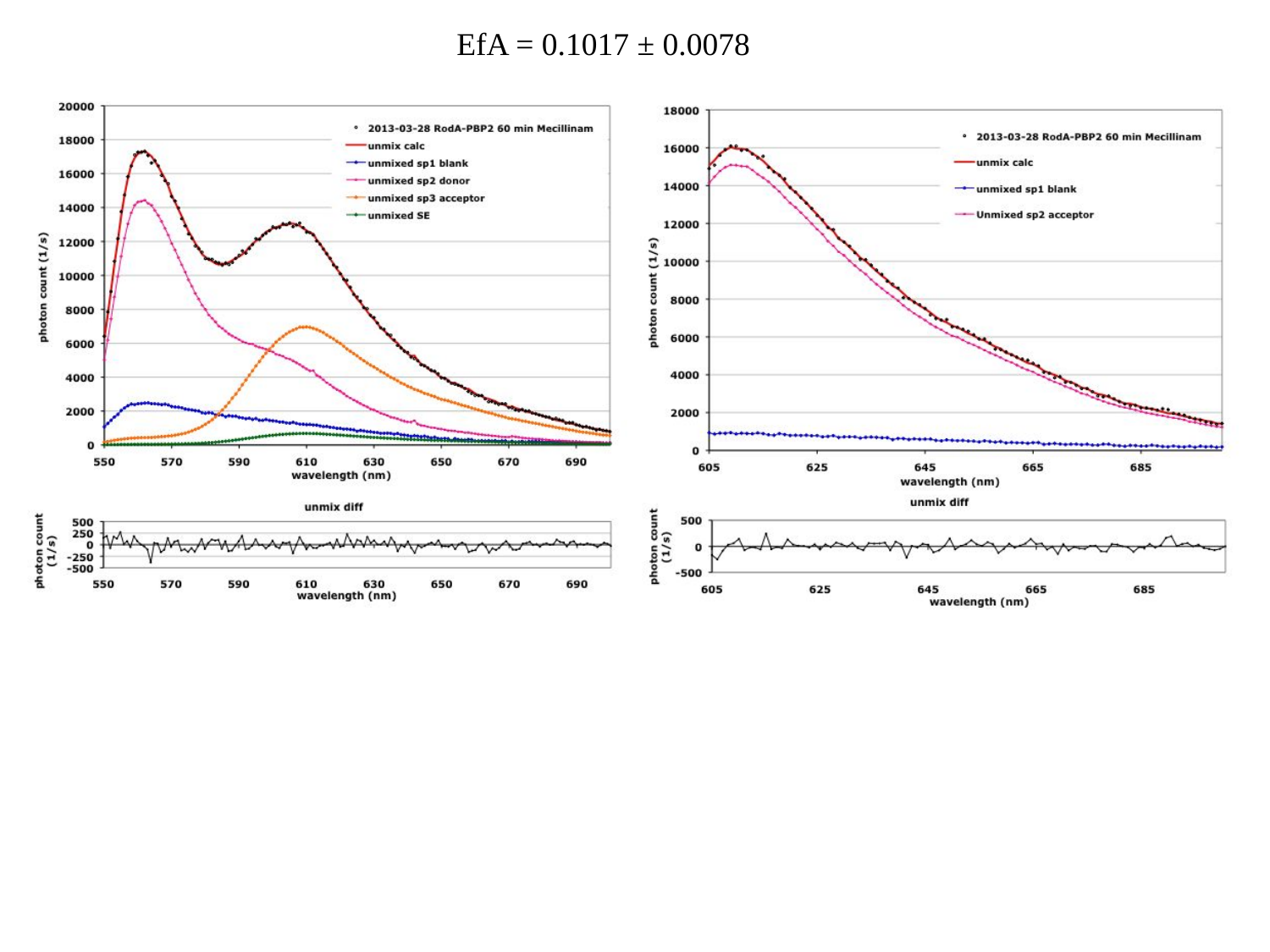

EfA = 0.1017 ± 0.0078
